# Recurrent Coding Sequence Variation Explains Only A Small Fraction of the Genetic Architecture of Colorectal Cancer

**DOI:** 10.1038/srep16286

**Published:** 2015-11-10

**Authors:** Maria N. Timofeeva, Ben Kinnersley, Susan M. Farrington, Nicola Whiffin, Claire Palles, Victoria Svinti, Amy Lloyd, Maggie Gorman, Li-Yin Ooi, Fay Hosking, Ella Barclay, Lina Zgaga, Sara Dobbins, Lynn Martin, Evropi Theodoratou, Peter Broderick, Albert Tenesa, Claire Smillie, Graeme Grimes, Caroline Hayward, Archie Campbell, David Porteous, Ian J. Deary, Sarah E. Harris, Emma L. Northwood, Jennifer H. Barrett, Gillian Smith, Roland Wolf, David Forman, Hans Morreau, Dina Ruano, Carli Tops, Juul Wijnen, Melanie Schrumpf, Arnoud Boot, Hans F A Vasen, Frederik J. Hes, Tom van Wezel, Andre Franke, Wolgang Lieb, Clemens Schafmayer, Jochen Hampe, Stephan Buch, Peter Propping, Kari Hemminki, Asta Försti, Helga Westers, Robert Hofstra, Manuela Pinheiro, Carla Pinto, Manuel Teixeira, Clara Ruiz-Ponte, Ceres Fernández-Rozadilla, Angel Carracedo, Antoni Castells, Sergi Castellví-Bel, Harry Campbell, D. Timothy Bishop, Ian P M Tomlinson, Malcolm G. Dunlop, Richard S. Houlston

**Affiliations:** 1 Colon Cancer Genetics Group, Institute of Genetics and Molecular Medicine, University of Edinburgh and MRC Human Genetics Unit, Western General Hospital Edinburgh, Crewe Road, Edinburgh, EH4 2XU, United Kingdom; 2 Division of Genetics and Epidemiology, The Institute of Cancer Research, Sutton, Surrey SM2 5NG, United Kingdom; 3 Wellcome Trust Centre for Human Genetics, University of Oxford, Oxford OX3 7BN, United Kingdom; 4 Centre for Population Health Sciences, University of Edinburgh, Teviot Place, Edinburgh EH8 9AG, United Kingdom; 5 Roslin Institute, University of Edinburgh, Easter Bush, Roslin EH25 9RG, United Kingdom; 6 Institute of Genetics and Molecular Medicine, University of Edinburgh and MRC Human Genetics Unit, Western General Hospital Edinburgh, Crewe Road, Edinburgh, EH4 2XU, United Kingdom; 7 Generation Scotland, Institute of Genetics and Molecular Medicine, University of Edinburgh, Western General Hospital Edinburgh, Crewe Road, Edinburgh, EH4 2XU, United Kingdom; 8 University of Edinburgh Centre for Cognitive Ageing and Cognitive Epidemiology, Department of Psychology, University of Edinburgh, Edinburgh EH8 9JZ, United Kingdom; 9 Section of Epidemiology & Biostatistics, Leeds Institute of Cancer and Pathology, University of Leeds, St James’s University Hospital, Leeds, UK; 10 Medical Research Institute, University of Dundee, Dundee, UK; 11 IARC, Cancer Surveillance Unit, Lyon, France; 12 Department of Pathology, Leiden University Medical Center, The Netherlands; 13 Department of Clinical Genetics, Leiden University Medical Center, The Netherlands; 14 Department of Human Genetics, Leiden University Medical Center, The Netherlands; 15 Department of Gastroenterology, Leiden University Medical Center, The Netherlands; 16 Institute of Clinical Molecular Biology, University Hospital Schleswig-Holstein, Campus Kiel, Kiel, Germany; 17 Institute of Epidemiology, Christian-Albrechts-University Kiel, Kiel; 18 Department of General and Thoracic Surgery, University Hospital Schleswig-Holstein, Campus Kiel, Kiel, Germany; 19 Medical Department 1, University Hospital Dresden, TU Dresden, Dresden, Germany; 20 Institute of Human Genetics, University Hospital Bonn, Bonn, Germany; 21 Division of Molecular Genetic Epidemiology, German Cancer Research Center (DKFZ), Im Neuenheimer Feld 580, D-69120 Heidelberg, Germany; 22 Center for Primary Health Care Research, Lund University, 205 02 Malmö, Sweden; 23 University of Groningen, University Medical Centre Groningen, Department of Genetics, PO Box 30001, 9700 RB Groningen, the Netherlands; 24 Department of Clinical Genetics, Erasmus Medical Center, Dr. Molewaterplein 50, 3015 GE Rotterdam, the Netherlands; 25 Department of Genetics, Portuguese Oncology Institute and Biomedical Sciences Institute (ICBAS), University of Porto, Porto, Portugal.; 26 Fundación Pública Galega de Medicina Xenómica (FPGMX), Centro de Investigación Biomédica en Red de Enfermedades Raras (CIBERER), Genomics Medicine Group, Hospital Clínico, 15706 Santiago de Compostela, University of Santiago de Compostela, Galicia, Spain; 27 Servei de Gastroenterologia, Hospital Clínic, Institut d’Investigacions Biomèdiques August Pi i Sunyer (IDIBAPS), Centro de Investigación Biomédica en Red de Enfermedades Hepáticas y Digestivas (CIBEREHD), University of Barcelona, 08036 Barcelona, Catalonia, Spain

## Abstract

Whilst common genetic variation in many non-coding genomic regulatory regions are known to impart risk of colorectal cancer (CRC), much of the heritability of CRC remains unexplained. To examine the role of recurrent coding sequence variation in CRC aetiology, we genotyped 12,638 CRCs cases and 29,045 controls from six European populations. Single-variant analysis identified a coding variant (rs3184504) in *SH2B3* (12q24) associated with CRC risk (OR = 1.08, P = 3.9 × 10^−7^), and novel damaging coding variants in 3 genes previously tagged by GWAS efforts; rs16888728 (8q24) in *UTP23* (OR = 1.15, P = 1.4 × 10^−7^); rs6580742 and rs12303082 (12q13) in *FAM186A* (OR = 1.11, P = 1.2 × 10^−7^ and OR = 1.09, P = 7.4 × 10^−8^); rs1129406 (12q13) in *ATF1* (OR = 1.11, P = 8.3 × 10^−9^), all reaching exome-wide significance levels. Gene based tests identified associations between CRC and *PCDHGA* genes (P < 2.90 × 10^−6^). We found an excess of rare, damaging variants in base-excision (P = 2.4 × 10^−4^) and DNA mismatch repair genes (P = 6.1 × 10^−4^) consistent with a *recessive* mode of inheritance. This study comprehensively explores the contribution of coding sequence variation to CRC risk, identifying associations with coding variation in 4 genes and *PCDHG* gene cluster and several candidate recessive alleles. However, these findings suggest that recurrent, low-frequency coding variants account for a minority of the unexplained heritability of CRC.

Heritable factors are thought to contribute to around 35% of the variation in risk of developing colorectal Cancer (CRC)^1^,^2^,^3^. High-penetrance mutations responsible for Mendelian disorders such as Lynch Syndrome, familial adenomatous polyposis and MUTYH associated polyposis have been shown to account for around 5% of all CRC. Genome-wide association studies (GWAS) have vindicated the notion that common genetic variants also contribute to CRC risk. Over 25 risk SNPs identified through GWAS^4^,^5^,^6^,^7^,^8^,^9^,^10^,^11^,^12^,^13^,^14^,^15^ are collectively responsible for only around 1% of CRC heritability^3^ and so much of the genetic contribution to CRC risk currently remains enigmatic. It has been proposed that low frequency variants in coding regions, may have substantial effects on risk and so may explain an appreciable proportion of the heritability of complex disease^16^. Conventional GWAS arrays have been sub-optimally configured to genotype such low frequency recurrent variation, whilst large-scale sequencing has been constrained by cost and data analysis bottlenecks.

Exome sequencing studies in multiple populations have enabled the assembly of catalogues of well-characterised single nucleotide variants within the coding sequence of genes. Genotyping arrays have been formatted into “exon” arrays specifically designed to interrogate recurrent genetic variation with putative impact on gene function. We set out to test the hypothesis that variation within gene coding sequences is associated with CRC risk, by making use of the recently introduced Illumina Exon array.

## Results

Post QC exome-wide analysis was based on 8,100 CRC cases and 21,820 controls from the six case-control series (Supplementary Tables 1 and 2). We also made use of genotypes for ~10,000 SNPs (~54% variants are non-synonymous) that were included in our previously published GWASs^8^,^10^, thus increasing power and providing additional exome array variant data on 4538 cases and 7225 controls (Supplementary Methods, Supplementary Table 3). Prior to the meta-analysis, we assessed the adequacy of the case-control matching and possibility of differential genotyping of cases and controls in individual studies using Quantile-Quantile (Q-Q) plots of test statistics (Supplementary Figure 6). Using data from the above 9 case-control series, we derived for each SNP joint odds ratios (ORs) and confidence intervals (CIs) in a meta-analysis under a fixed-effects model and determined the associated *P* values. Overall 72,162 non-monomorphic post-QC variants observed in at least 2 studies contributed to the combined meta-analysis totalling 12,638 cases and 29,046 controls (Supplementary Table 1). Of these variants, 29,117 variants were rare (MAF < 1%) and 32,809 variants exhibited MAF < 5%. We found no appreciable inflation of test statistics for the meta-analysis as a whole, λ_90%bottom_ = 0.98, thereby excluding significant differential genotyping or cryptic population substructure (See Q-Q plot in Supplementary Figure 7)^8^,^10^,^13^.

### Single variant analysis

17 variants showed evidence for an association with CRC which exceeded Bonferroni-corrected exome-wide threshold of statistical significance (Table 1, Supplementary Table 4, Supplementary Figure 7), 4 of these 17 variants were non-synonymous missense variants: (rs3184504 (p.Trp263Arg) in *SH2B3* (12q24; OR = 1.08, P = 3.9 × 10^−7^, effect allele frequency (EAF) = 0.52); rs16888728 (p.Pro215Gln) in *UTP23* (8q24; OR = 1.15, P = 1.4 × 10^−7^, EAF = 0.10); two variants in *FAM186A* (12q13) - rs6580742 (p.Met2193Ile, OR = 1.11, P = 1.2 × 10^−7^, EAF = 0.19) and rs12303082 (p.Lys187Gln, OR = 1.09, P = 7.4 × 10^−8^, EAF = 0.36)). Another variant within 12q13 loci rs1129406 (12q13; OR = 1.11 P = 8.3 × 10^−9^, EAF = 0.41) is located within a splice region of *ATF1*. The rs3184504 association highlights a novel CRC risk locus (Table 1, Supplementary Figure 8). The p.Trp263Arg amino acid change resides in exon 3 of the SH2B adaptor protein and is predicted to be benign and tolerated by PolyPhen^17^ and SIFT^18^. Though predicted to be located within a transcription factor binding site (*POLR2A*) in lymphoblastoid, leukaemia and glioblastoma cell lines, it seems unlikely affect binding according to RegulomeDB (score 3a)^19^ or influence expression of *SH2B3* in lymphoblastoid cell lines^20^,^21^ and other tissues^22^,^23^. Conditional analysis showed that rs3184504 genotype was sufficient to explain all of the effect at the 12q24 risk locus (Supplementary Table 5).

The 4 other novel SNPs rs16888728, rs6580742, rs12303082 and rs1129406 map to the previously described 8q24.11^12^,^24^ and 12q13.12 loci^10^ (Table 1). rs16888728 is located within exon 3 of *UTP23* (8q23.3, 117783975, p.Pro215Gln) and is in moderate linkage disequilibrium (LD) with rs16892766 (8q23.3, 117630683)^24^ (D’ = 0.63, r^2^ = 0.30). Mutual adjustment was unable to distinguish the effects of rs16888728 on CRC risk from the previously described GWAS association, suggesting rs16892766 to be a primary signal (rs16888728, OR_cond_ = 0.99, P_cond_ = 0.83; rs16892766, OR_cond_ = 1.27, P_cond_ = 5.3 × 10^−10^) (Supplementary Table 6).

Detailed analysis of the 12q13 locus encompassing coding variants in *ATF1* and *FAM186A* showed that three new variants are within a region of fairly extensive linkage disequilibrium (LD) (r^2^ = 0.31–0.68, D’ = 0.92–1) and in moderate LD with rs11169552, a previously identified through GWAS^10^ CRC risk locus (r^2^ = 0.08 − 0.24, D’ = 0.95–0.99). Both rs6580742 and rs12303082 are missense variants located within the exon 1 (rs6580742, chr12:50727811, p.Met2193Ile) and exon 3 (rs12303082, chr12:50754563, p.Lys187Gln) of *FAM186A*. Strongest signal at the locus (rs1129406) is a synonymous coding variant in *ATF1* located within the splice region of gene, though it is unclear if the normal splicing of the gene is affected by the variant. rs6580742 is located within DNaseI hypersensitivity cluster and in eQTL with DIP2B and KIAA1463 expression in lymphoblastoid cell lines^19^,^25^,^26^ and cis-eQTL with ATF1 expression in esophagus mucosa, subcutaneous adipose tissue, tibial artery^22^,^23^. It is likely to affect binding according to RegulomeDB (score 1f)^19^,^27^. Conditional analyses indicate that all the association signals, including previously identified rs11169552^10^ (OR = 1.08, P = 2.55 × 10^−5^, OR_cond_ = 1.02, P_cond_ = 0.35,EAF = 0.73), are explained by rs1129406, the splice region variant in *ATF1* (Supplementary Table 7).

The remaining 10 SNPs in non-coding regions had been identified through our previous GWAS studies of CRC^10^,^11^,^13^,^28^,^29^,^30^. We subsequently applied conditional analysis to interrogate all CRC risk loci highlighted by the current study but found no evidence of multiple signals at 1q41, 8q24.21, 15q13.3, 18q21.1, 19q13.11, 20p12.3 and 20q13.33 (Supplementary Tables 8–14).

We further explored if rs1129406 (*ATF1*, 12q13), rs12303082 (*FAM186A*, 12q13), rs6580742 (*FAM186A*, 12q13), rs16888728 (*UTP23*, 8q24) and rs3184504 (*SH2B3*, 12q24) genotypes affect the CRC risk differentially by sex, age at diagnosis, tumor site, stage and MSI status (Supplementary Table 15). Intriguingly, we found that rs16888728 is significantly associated with gender in case-only analysis (OR = 1.21, P = 5.6 × ^−4^) with no effect on CRC risk in males in case-control analysis (OR = 1.28, P = 5 × 10^−8^ in women and OR = 1.06 and P = 0.14 in men).

### Gene-based analysis

Following on from these single variant analyses we conducted a gene-based analysis for rare (MAF < 1%) and low-frequency (MAF < 5%) variants observed in at least two cohorts (Supplementary Figure 9, Table 2). Meta-analysis of SKAT-O results showed some evidence of inflation (λ = 1.45 in analysis for low –frequency variants). Among the genes showing evidence of association in low-frequency variants analysis were tandemly located genes from protocadherin gamma gene cluster (*PCDHGA3, PCDHGA2, PCDHGA1, PCDHGA4, PCDHGB1*, 5q31.3, P < 2.9 × 10^−6^). The details of the SNPs contributing to *PCDHG* associations are given in Supplementary Table 16. None of the genes reached significance in rare – variant analysis.

Gene-ontology (GO) enrichment analysis implicated homophilic cell adhesion genes in CRC development (Supplementary Table 17).

### Search for candidate high-penetrance CRC alleles

Next, we searched for rare high penetrance CRC variants by analysis of rare damaging variants present in more than 3 CRC cases, but absent from controls. In the analysis of dominant alleles, we observed truncating variants in *NWD1*, *CD1A*, *ZNF594*, *DNAH9*, *ZNF418*, *ABTB1* and *HIST1H3A* and two missense variants in *GCN1L1* (Supplementary Table 18). We also assessed the contribution of rare recessive alleles present in >3 cases, but absent in controls (Supplementary Table 18). Notable among these homozygotes were stop codon (p.Tyr90*) in the base excision repair gene, *NTHL1*, as well as homozygous missense variants in the DNA mismatch-excision repair gene, *PMS1* (p.Thr75Ile) (Supplementary Figure 10). Overall we saw an excess of rare homozygous variants in base excision repair (16/8100 cases vs. 10/21820 controls, OR = 4.31; P = 2.4 × 10^−4^) and mismatch repair genes (11/8100 cases vs. 5/21820 controls, OR = 5.93, P = 6.1 × 10^−4^) in cases (Supplementary Table 19).

We also sought evidence of compound heterozygosity in cases and identified two damaging *NOTCH2* variants and three damaging variants in *DNAJC17* (DnaJ (Hsp40) homolog, subfamily C, member 17) that were observed to be present in heterozygous state at least twice in 2 and more cases, but absent in controls (Supplementary Table 20). *NOTCH2* is regulated by Wnt signalling and known to have lower expression in colorectal and ovarian cancer^31^.

## Discussion

We have identified coding variation in 4 genes (*SH2B3, UTP23, FAM186A*, *ATF1)* and *PCDHG* gene cluster that contribute to the risk of developing CRC. Three of the 4 genes with new coding variants influencing CRC risk had been identified by previous GWAS SNPs^10^,^12^,^24^. Novel association between the coding variant (rs3184504) in the *SH2B3* gene has been described during the process of preparation and review of this manuscript in an independent meta-analysis^32^. Perhaps the most interesting finding of this well-powered study is the observation that very few recurrent coding sequence variants contribute to CRC risk, and certainly not with major effect size (OR > 2.5).

The association between CRC risk and the adaptor protein, SH2B3, is interesting, since rs3184504 results in a predicted benign non-synonymous amino acid substitution (p.Trp263Arg) within the plekstrin homology domain of SH2B3. SH2B3 is induced upon JAK-STAT3 phosphorylation and is expressed at high levels in haematopoietic cells, but only at low levels in the normal colon. The protein is a regulator of cytokine signals at the cell surface through tyrosine kinase signalling cascades and is thought to act as a negative regulator of such signals at the cell surface to impart an anti-proliferative effect. A consanguineous family has been reported which segregates a germline frameshift mutation in the Plekstrin homology domain of SH2B3. Homozygous individuals developed various autoimmune phenotypes and one sibling developed acute lymphoblastic leukaemia (ALL) as an infant^33^. Somatic *SH2B3* mutations have also been identified in 3% of ALL, suggesting that *SH2B3* loss plays a role in initiation and progression of human leukaemia through dysregulated cytokine signalling. Interrogation of TCGA and Broad Institute sequence data from colorectal adenocarcinomas^34^,^35^,^36^ did not identify an excess of somatic mutations in SH2B3 (0.69% of samples carry deleterious mutations or copy number variations), suggesting that SH2B3 mutations are not drivers in CRC progression^33^. Genetic variation at the SH2B3 gene locus has been associated with various autoimmune related disorders including hepatitis^37^, rheumatoid arthritis^38^, hypothyroidism^39^, type 1 diabetes^40^, vitiligo^41^, rheumatoid arthritis and coeliac syndrome^42^, suggesting that SH2B3 dysfunction may be involved in mediating disordered immune function and thereby play a role in cancer susceptibility. Interestingly, SH2B3 is over-expressed in ovarian tumour cells with evidence for a role in activating signal transduction^43^. SH2B3 expression status may have paradoxical effects in cancer, dependent on cellular context.

The variant in *UTP23* (rs16888728) also exerts a modest effect on CRC risk. The UTP23 transcript is expressed at modest levels in many tissue types. It has sequence homology to a yeast protein involved in ribosomal RNA processing and ribosome biogenesis. As such, it may be involved in alternative splicing, although very little is known about the functional role of the human protein. The coding variant (rs16888728) is located within exon 3 of *UTP23* and results in a non-conserved amino acid substitution (p.Pro215Gln, GERP score = −0.543). Conditional analysis was unable to distinguish the effects of rs16888728 on CRC risk from that of the previously described^24^ GWAS association (rs16892766). Interrogation of tumour sequence databases reveals no significant excess of mutations in CRC (<1% prevalence)^34^,^35^,^36^. However, *UTP23* is amplified in ~5% of CRC tumours^35^,^36^ with significant correlation between UTP1 mRNA expression and copy number variation.

The SNP rs1129406, a splice site variant in *ATF1*, appears to explain the association signal at the 12q13 locus, including that of a previous signal identified by GWAS (rs11169552)^10^. ATF1 is a transcription factor that, when phosphorylated, induces transcriptional transactivation of target genes. Fusion of *ATF1* with the Ewing’s Sarcoma gene, or with FUS, results in continuous signaling and sarcomatous tumour formation. Common variation has not been associated with other cancers, however significant cis-eQTL with ATF1 was detected for this variant in esophagus mucosa, subcutaneous adipose tissue and tibial artery^22^,^23^. Whilst there are no excess of somatic mutations in CRC tissue in TCGA or Broad data, rs1129406 may be the causative variant that explains the previous GWAS signal. The relationship of *FAM186A* to CRC risk is somewhat opaque, as very little is known about this gene. *FAM186A* appears to be a protein coding gene, rather than a lncRNA. Hence we cannot exclude the possibility that the effect is mediated through regulatory effects.

The gene-based test, SKAT-O, highlighted several genes from protocadherin gamma (PCDHG) gene cluster on chromosome 5 exhibiting a composite excess of coding variants and thereby indicating the gene is associated with CRC risk. Somatic genomic missense and nonsense mutations in one of the identified genes are present in 11.8% of CRC cases and up to 31% of all skin cuteneous melanomas (according to The Cancer Genome Atlas data)^35^. PCDHG gene cluster encodes 22 genes divided into 3 subfamily (A,B and C) based on sequence similarities with multiple transcripts generated by alternative splicing^44^. PCDH expression is observed in colon and long range epigenetic silencing of PCDH cluster region has been described in Wilm’s tumours^45^, breast cancers^46^ and colorectal adenomas and carcinomas^47^. Hence, PCDH genes play role of tumour suppressor and silencing mutations might be expected to have tumour-promoting effects. Whilst PCDHG cluster genes are strong candidates based on the analysis presented in this study, further work is required to confirm the role of these genes in cancer predisposition.

The identification of damaging alleles acting as rare recessive traits in genes that participate in DNA repair, with known paradigms in CRC susceptibility, such as *NTHL1* (p.Tyr90*) and *PMS1* (p.Thr75Ile) clearly require further study as these represent strong candidate recessive alleles. Recently *NTHL1* loss-of-function germline mutation has been described in families with adenomatous polyposis and progression to CRC inherited in recessive mode^48^, thus suggesting that the observed association is real and our search for rare damaging alleles is a successful approach to identify candidate variants. The observed excess of rare damaging variants in base-excision and mismatch repair genes suggests that the clinical importance of moderately penetrant, disease-causing, variants in DNA repair genes may be underestimated. However, further studies will require even larger sample sizes, given the rarity of the alleles, unless sequencing can identify new alleles in addition to those catalogued here. Indeed, many of the genes with damaging variants represent strong candidates for validation in exome and whole genome sequencing efforts.

Given the expectation that uncommon functional variation might be associated with CRC risk, with larger effect size than common variation, it is surprising that we have identified so few new coding sequence variants, and that all of these exert modest effect sizes (OR 1.08–1.15). In a linear-mixed model analysis (Supplementary Material), we estimated that the genetic variants identified though previous GWAS and significant in our meta-analysis explain approximately 1.5 ± 0.7% of the total phenotypic variance on the liability scale, while the newly identified variants account for only 0.4% of the total variance.

The Infinium Human Exome BeadChip 12v1.0 or 12v1.1 (Illumina Inc.) array was configured to identify coding sequence variants most likely to have functional consequences. Despite of its attractiveness as a cheap alternative to exome sequencing, exome array has some limitations and is not able to offer complete whole exome coverage of all possible functional variants and indels. Importantly, exome array was designed based on exome sequencing of 12,000 samples and enriched for multiple outcomes such as cardiovascular disease, obesity, diabetes, autism and cancer^49^, which may not be representative of our cohorts. There were some differences in the genotyping quality between various versions of arrays used in the analyses and many variants did not pass stringent quality control criteria. Around 70,000 SNPs were non-monomorphic in European populations, present in at least two studies and passed our QC measures.

The focus on genetic variants with potential detrimental functional consequences should also enhance the *a priori* likelihood of pathogenicity. Though limited in detection of indels with only 136 present on the chip, the study was well powered to detect plausible effect sizes and allele frequencies (Supplementary Figure 11). Indeed, the study size had 80% power to detect an OR > 3 provided the MAF was >0.001 and an OR odds >1.8 if the MAF was 0.005. Whilst larger studies and/or meta-analysis might identify further coding variants with functional effects, the paucity of findings of recurrent low frequency coding variation impacting on CRC risk is intriguing. Because the causative gene mutations have been characterised for almost all dominant high penetrance CRC families, it seems unlikely that rare recurrent alleles in European populations have yet to be identified with large effects (OR > 5), apart from private mutations or recessive traits that are unlikely to be discovered through designed commercial arrays. Hence, population-specific custom exome arrays as well exome and genome sequencing of trios and families may be a way forward to identify recurrent rare genetic variation of moderate effect of risk and private mutations.

## Materials and Methods

### Study populations

The study was based on six independent case control series from European populations including Scotland (3,616 cases and 10,312 controls), England (4,558 cases and 11,249 controls), Germany (284 cases and 1,100 controls), Holland (480 cases and 480 controls), Spain (300 cases and 300 controls) and Portugal (200 cases and 200 controls). Details regarding these participating studies are described in the Supplementary Data (available online). All cases had histologically confirmed adenocarcinoma of the colon or rectum (codes 153 or 154 International Classification of Diseases (ICD), 9th revision or ICD10 C18, C19 or C20 codes). The study was undertaken at participating centres with written informed consent in accordance with respective Institutional Review Boards (IRB)/Ethics Committees.

To enhance our power we made use of previously published GWASs^8^,^10^ thus providing ~10.000 exome array variant data on 3,549 cases and 3,698 controls from UK1 and UK2 studies, 3,158 cases and 3,073 controls from Scotland Phase1, Scotland Phase2 and Scotland Phase3, and 1,794 cases and 2,686 controls from the VQ58 study^8^,^13^ (Supplementary Methods, Supplementary Tables 2, 3). After quality control and exclusion of expected and unexpected duplicates between studies we ended up with exome array variant data on 3,033 cases and 3,690 controls from UK1 and UK2 studies, 556 cases and 2,997 controls from Scotland Phase1, Scotland Phase2 and Scotland Phase3, and 949 cases and 538 controls from the VQ58 study^8^,^13^. Study details, details of genotyping, quality control procedures, sample and SNPs exclusion for these GWAS-focussed studies have been published previously^8^ (Supplementary Data, Supplementary Tables 2, 3).

### Exome Array Genotyping and Quality Control

DNA was extracted from EDTA-venous blood samples using standard methodologies at each centre. Genotyping was performed using the Infinium Human Exome BeadChip 12v1.0 or 12v1.1 (Illumina Inc., San Diego, CA), with genotype calling using Illumina GenCall for HumanExome-12v1.0 and HumanExome-12v1.1 versions called separately. Generation Scotland controls and a subset of the cases from the SOCCS study were genotyped using OmniExpressExome BeadChip 8v1.1 or 8v1.2^50^ (Illumina Inc., San Diego, CA). A summary of the array SNP content^51^,^52^ and the respective SNP inventory^53^ have been provided previously. Standard quality procedure were applied, with further details of sample and probe exclusion in Supplementary Material and Supplementary Table 2. We compared MAF and genotyping call genotyping call rates between different version of arrays used in the current study and excluded all variants that showed some evidence of differences (Supplementary Figures 1,3). Additionally, we compared allele frequency to the 1000G data and UK exome array consortium (Supplementary Figure 2). Following standard quality-assurance and quality control measures this collaborative initiative provided information on 12,638 CRCs cases and 29,045 controls (Supplementary Table 1).

### Statistical analysis

We designed the study according to an estimate of the sample size required to detect plausible effect sizes (OR = 1.5–5.0) at various rare allele frequencies (>0.001). Following completion of the study and all QC measures, we re-estimated statistical power for a given sample size using QUANTO version 1.2.4^54^ for the main effect of genetic variant and the log-additive model of inheritance stipulating a *P*-value of 5.5 × 10^−7^, which corresponds to Bonferroni-corrected exome-wide level of significance.

The association between individual variants and risk of CRC was evaluated in initial data analysis using unconditional logistic regression under a log-additive model of inheritance for each study separately. To examine whether associations at each identified locus were independent, we conducted conditional analysis by controlling for allelic dosage for the most significantly associated SNP at the locus. We subsequently applied conditional analysis to interrogate following CRC risk loci highlighted by the current study: 1q41 controlling for rs6687758, 8q23.3 controlling for rs16892766 and/or rs16888728, 8q24.21 controlling for rs10505477, rs6983267 and/or rs7014346, 11q32.1 controlling for rs3802842, 12q13.12 controlling for rs6580742, rs12303082 and rs1129406, 12q24.12 controlling for rs3184504, 14q22.2 controlling for rs4444235, 15q13.3 controlling for rs4779584, 18q21.1 controlling for rs4939827, 19q13.11 controlling for rs10411210, 20p12.3 controlling for rs961253 and 20q13.33 controlling for rs4925386.

Individual study effect estimates (Odds ratios (OR) and associated 95% confidence intervals (CIs)) derived from logistic regression were combined in a meta-analysis. We used a fixed effect inverse variance weighting model for meta-analysis to maximize discovery power of the current study^55^. Only non-monomorphic variants observed in at least two studies were included in the meta-analysis. We tested for over-dispersion of *P*-values in the meta-analysis by generating quantile-quantile (QQ) plots and deriving an inflation factor (λ). Cochran’s Q statistic was used to test for heterogeneity and the I^2^ statistic to quantify the proportion of the total variation due to heterogeneity. I^2^ values ≥ 75% were considered to indicate excessive heterogeneity^56^ and variants displaying I^*2*^ values > 75% in were excluded from further analysis. Taking all the above measures into account, 72,162 SNPs remained in the analysis, equating to a Bonferroni-corrected exome-wide threshold of statistical significance of 5.55 × 10^−7^. This is conservative given the likely linkage disequilibrium between some variants. We further examined top variants and excluded those that showed obvious problems with clustering and differences in clustering between versions of genotyping platforms in our analysis. This included monomorphic rs1058065 (exm2255298).

Association by sex, age, stage (invasive, non-invasive), MSI status and tumour site (rectal [ICD9:154], colonic [ICD9:153]) for the top new variants were further explored using ordered logistic regression in case-only analysis. All statistical tests were two-sided.

### Gene based and pathway analysis

To explore the effects of more than one variant in the same gene on CRC risk, we used the small-sample-adjusted unified test, SKAT-O^57^ with default weight on rare variants. All variants observed in at least two studies contributed to the SKAT-O results. We performed analyses for rare (MAF > 1%) and low frequency variants (MAF below 5%) including all and only High and Moderate effects as annotated by SnpEff  58. Due to the different number of variants in each individual study we performed SKAT-O test separately for each individual study and combined summary statistics from individual SKAT results in a meta-analysis using “MetaSKAT” package in R^59^ Similarly to single-variant analysis we tested for over-dispersion of *P*-values by generating QQ plots and deriving an inflation factor (λ). To account for multiple testing in these gene-based tests, we set the significance threshold to be P < 2 × 10^−6^ to reflect Bonferroni correction for the 23,280 genes examined. These 23,280 genes were selected on the base of the presence of 2 and more variants per gene and unique mapping coordinates. We further examined top genes and excluded those that were driven by single variant with the differences in clustering between versions of genotyping platforms in our analysis. This included monomorphic rs1058065 (*EIF2B4*) .

Further, we investigated variants contributing to the gene-based test. To determine whether genes identified in SKAT-O were enriched for particular molecular pathways, we performed a gene ontology (GO) enrichment analysis on a sorted by p value list of genes , using **G**ene **O**ntology en**RI**chment ana**L**ysis and visua**L**iz**A**tion tool (GOrilla)^60^,^61^.

### Search for candidate high-penetrance CRC alleles

We considered the possibility that rare damaging variants represented on the exome array might confer high-penetrance susceptibility to CRC and conducted exploratory data analysis. We reasoned on the basis of pre-existing empiric data that any dominant alleles would be likely to have frequencies of <0.1%, whereas recessive alleles would have frequencies of <2% in controls. Dominant alleles were filtered from the entire variant set as follows: [1] predicted not to be benign/tolerated by both SIFT^18^ and PolyPhen2^17^ or nonsense variants; [2] excluded probable miscalled SNPs through visual inspection of genotyping clusters; [3] absent in controls to ensure inclusion of potentially high penetrance risk alleles. Recessive alleles were filtered from the entire variant set as follows: [1] predicted not benign or tolerated by both SIFT^18^ and PolyPhen2^17^; [2] excluded probable miscalled SNPs through visual inspection of genotyping; [3] homozygotes absent in controls to ensure inclusion of potentially high penetrance risk alleles; [4] minor allele frequency ≤ 0.02 in controls.

We evaluated effect of rare damaging variants under dominant or recessive model of inheritance using Fisher’s exact test in a pooled analysis. Due to the limited number of rare damaging variants on traditional GWAS platforms, we included in the analysis case-control series genotyped using Exome Array only (8100 cases/21820 controls). We also looked for evidence of an excess of compound heterozygosity for rare damaging variants in cases compared to controls. The compound heterozygous list was filtered from the entire set of heterozygous variants as follows: (1) excluded probable miscalled SNPs through visual inspection of genotyping clusters, [2] predicted not to be benign/tolerated by both SIFT^18^ and PolyPhen2^17^, (3) number of rare damaging heterozygotes per gene in controls ≤ 1, (4) minor allele frequency ≤ 2% in controls. We further look for excess of rare damaging homozygous variants in DNA repair pathways by counting number of homozygous rare variants in cases and controls and testing significance by Fisher exact test.

Although this study did not have power to detect such alleles by association testing or by gene burden tests, we catalogued all candidate alleles that fulfilled these criteria.

## Additional Information

**How to cite this article**: Timofeeva, M. N. *et al*.Recurrent Coding Sequence Variation Explains Only A Small Fraction of the Genetic Architecture of Colorectal Cancer. *Sci. Rep*.**5**, 16286; doi: 10.1038/srep16286 (2015).

## Supplementary Material

Supplementary Information

## Figures and Tables

**Table 1:** Results of meta-analysis for variants reaching exome-wide level of significance (4 × 10^-7^) under a fixed effects model.

SNP rsID	Gene	Annotation	CHR	BP	Risk Allele	Reference Allele	EAF(cases/controls)	N studies	N cases	N controls	OR	P value	P value Bonferroni adjusted
rs1129406	*ATF1*	coding-synon	12	51203371	A	G	0.43/ 0.40	6	4730	12603	1.11	8.30 × 10^−9^	7.44 × 10^−04^
rs12303082	*FAM186A*	missense	12	50754563	A	C	037/0.35	9	10207	19886	1.09	7.40 × 10^−8^	6.63 × 10^−03^
rs6580742	*FAM186A*	missense	12	50727811	A	G	0.20/0.19	9	12539	29208	1.11	1.20 × 10^−7^	0.01
rs16888728	*UTP23*	missense	8	117783975	A	G	0.11/0.10	8	10621	26779	1.15	1.40 × 10^−7^	0.01
rs3184504	*SH2B3*	missense	12	111884608	G	A	0.53/0.51	9	12530	29197	1.08	3.90 × 10^−7^	0.03

EAF – effect allele frequency.

**Table 2:** Meta-analysis of gene-based (SKAT-O) tests.

SetID	Gene	N of variants #	Description	Chr	band	p.value
(A) low frequency (MAF < 5%) variants (n = 16,585)						
ENSG00000254245	*PCDHGA3*	89	protocadherin gamma subfamily A, 3	5	q31.3	7.29E-07
ENSG00000081853	*PCDHGA2*	90	protocadherin gamma subfamily A, 2	5	q31.3	7.49E-07
ENSG00000204956	*PCDHGA1*	91	protocadherin gamma subfamily A, 1	5	q31.3	7.86E-07
ENSG00000254221	*PCDHGB1*	82	protocadherin gamma subfamily B, 1	5	q31.3	1.43E-06
ENSG00000262576	*PCDHGA4*	79	protocadherin gamma subfamily A, 4	5	q31.3	2.91E-06
(B) High and Moderate low frequency (MAF < 5%) variants (n = 16,081)						
ENSG00000254245	*PCDHGA3*	83	protocadherin gamma subfamily A, 3	5	q31.3	2.59E-06
ENSG00000081853	*PCDHGA2*	84	protocadherin gamma subfamily A, 2	5	q31.3	2.79E-06
ENSG00000204956	*PCDHGA1*	85	protocadherin gamma subfamily A, 1	5	q31.3	2.96E-06

Top significant results for SKAT-O gene-based test for different subsets. We used Bonferroni correction to identify Exome-Wide level of significance for each of the subgroup separately. Only variants, which were observed in at least two independant studies, were included in the analysis. Genes with less than 2 variants per gene were exluded. Variants were defined High and Moderate accordind to classification adapted by SnpEff. # N of variants is based by the number of SNPs located within the genes and may vary by study, e.g. in case of monomorphic alleles.
