# Supplementary material for: Recurrent Coding Sequence Variation Explains Only A Small Fraction of the Genetic Architecture of Colorectal Cancer
Source: Sci Rep. 2015 Nov 10;5:16286. doi: 10.1038/srep16286 (PMC4639776; doi:10.1038/srep16286)
Supplement: Supplementary Information [file srep16286-s1.pdf]

# Recurrent Coding Sequence Variation Explains Only A Small fraction of the Genetic Architecture of Colorectal Cancer

¶[1]Maria N Timofeeva, ¶[2]Ben Kinnersley, [1]Susan M Farrington, [2]Nicola Whiffin, [3]Claire Palles, [1]Victoria Svinti, [2]Amy Lloyd, [3]Maggie Gorman, [1]Li-Yin Ooi, [2]Fay Hosking, [3]Ella Barclay, [1]Lina Zgaga, [2]Sara Dobbins, [3]Lynn Martin, [1,4]Evropi Theodoratou, [2]Peter Broderick, [5,6]Albert Tenesa, [1]Claire Smillie, [6]Graeme Grimes, [6]Caroline Hayward, [6,7]Archie Campbell, [6,7] David Porteous, [8]Ian J Deary, [6,8]Sarah E Harris, [9]Emma L. Northwood, [9] Jennifer H. Barrett, [10]Gillian Smith, [10]Roland Wolf, [11]David Forman, [12]Hans Morreau, [12]Dina Ruano, [13]Carli Tops, [14]Juul Wijnen, [12]Melanie Schrumpf, [12]Arnoud Boot, [15]Hans FA Vasen, [13] Frederik J Hes, [12]Tom van Wezel, [16]Andre Franke, [17]Wolgang Lieb, [18]Clemens Schafmayer, [19]Jochen Hampe, [19]Stephan Buch, [20]Peter Propping, [21,22]Kari Hemminki, [21,22]Asta Försti, [23]Helga Westers, [23,24]Robert Hofstra, [25]Manuela Pinheiro, [25]Carla Pinto, [25]Manuel Teixeira, [26]Clara Ruiz-Ponte, [26,3] Ceres Fernández-Rozadilla, [26]Angel Carracedo, [27]Antoni Castells, [27]Sergi Castellví-Bel, §[1,4]Harry Campbell, §[9]Tim Bishop, §[3]Ian PM Tomlinson, §[1]Malcolm G Dunlop* and §[2]Richard S Houlston.

[1] Colon Cancer Genetics Group, Institute of Genetics and Molecular Medicine, University of Edinburgh and MRC Human Genetics Unit, Western General Hospital Edinburgh, Crewe Road, Edinburgh, EH4 2XU, United Kingdom.

[2] Division of Genetics and Epidemiology, The Institute of Cancer Research, Sutton, Surrey SM2 5NG, United Kingdom.

[3] Wellcome Trust Centre for Human Genetics, University of Oxford, Oxford OX3 7BN, United Kingdom.

[4]Centre for Population Health Sciences, University of Edinburgh, Teviot Place, Edinburgh EH8 9AG, United Kingdom.

[5] Roslin Institute, University of Edinburgh, Easter Bush, Roslin EH25 9RG, United Kingdom.

[6]Institute of Genetics and Molecular Medicine, University of Edinburgh and MRC Human Genetics Unit, Western General Hospital Edinburgh, Crewe Road, Edinburgh, EH4 2XU, United Kingdom.

[7]Generation Scotland, Institute of Genetics and Molecular Medicine, University of Edinburgh, Western General Hospital Edinburgh, Crewe Road, Edinburgh, EH4 2XU, United Kingdom.

[8]University of Edinburgh Centre for Cognitive Ageing and Cognitive Epidemiology, Department of Psychology, University of Edinburgh, Edinburgh EH8 9JZ, United Kingdom.

[9]Section of Epidemiology & Biostatistics, Leeds Institute of Cancer and Pathology, University of Leeds, St James's University Hospital, Leeds, UK.

[10]Medical Research Institute, University of Dundee, Dundee, UK.

[11]IARC, Cancer Surveillance Unit, Lyon, France.

[12]Department of Pathology, Leiden University Medical Center, The Netherlands.

[13]Department of Clinical Genetics, Leiden University Medical Center, The Netherlands.

[14]Department of Human Genetics, Leiden University Medical Center, The Netherlands.

[15]Department of Gastroenterology, Leiden University Medical Center, The Netherlands.

[16]Institute of Clinical Molecular Biology, University Hospital Schleswig-Holstein, Campus Kiel, Kiel, Germany.

[17]Institute of Epidemiology, Christian-Albrechts-University Kiel, Kiel.

[18]Department of General and Thoracic Surgery, University Hospital Schleswig-Holstein, Campus Kiel, Kiel, Germany.

[19]Medical Department 1, University Hospital Dresden, TU Dresden, Dresden, Germany.

[20]Institute of Human Genetics, University Hospital Bonn, Bonn, Germany.

[21]Division of Molecular Genetic Epidemiology, German Cancer Research Center (DKFZ), Im Neuenheimer Feld 580, D-69120 Heidelberg, Germany.

[22]Center for Primary Health Care Research, Lund University, 205 02 Malmö, Sweden.

[23]University of Groningen, University Medical Centre Groningen, Department of Genetics, PO Box 30001, 9700 RB Groningen, the Netherlands.

[24]Department of Clinical Genetics, Erasmus Medical Center, Dr. Molewaterplein 50, 3015 GE Rotterdam, the Netherlands.

[25]Department of Genetics, Portuguese Oncology Institute and Biomedical Sciences Institute (ICBAS), University of Porto, Porto, Portugal.

[26]Fundación Pública Galega de Medicina Xenómica (FPGMX), Centro de Investigación Biomédica en Red de Enfermedades Raras (CIBERER), Genomics Medicine Group, Hospital Clínico, 15706 Santiago de Compostela, University of Santiago de Compostela, Galicia, Spain.

[27]Servei de Gastroenterologia, Hospital Clínic, Institut d'Investigacions Biomèdiques August Pi i Sunyer (IDIBAPS), Centro de Investigación Biomédica en Red de Enfermedades Hepáticas y Digestivas (CIBEREHD), University of Barcelona, 08036 Barcelona, Catalonia, Spain.

***Corresponding author**
**E-mail: malcolm.dunlop@igmm.ed.ac.uk**

¶These authors contributed equally to this work as 1st authors.

§These authors contributed equally to this work.

## Supplementary Methods

### *Exome Array Analysis*

The study was undertaken at participating centres with written informed consent in accordance with respective Institutional Review Boards (IRB)/ Ethics Committees (CORGI REC 06/Q1702/99; SOCCS REC 11/SS/0109; LBC1921 LREC/1998/4/183; LBC1936 MREC/01/0/56; NSCCG REC 02/0/97, LUMC (P01-019), Groningen (MEC97/02/037f), Germany (IRB - AZ LD4-16.1/03.001, and ethics AZ A 156/03), EPICOLON (Hospital Clínic, 07/03/2000, ref. 460).

All cases had histologically confirmed adenocarcinoma of the colon or rectum (codes 153 or 154 International Classification of Diseases (ICD), 9th revision or ICD10 C18, C19 or C20 codes).

The study was based on six independent case control series. The Scottish series comprised 3,517 cases (2013 male, mean age 58yrs) from the Scottish colorectal cancer study (SOCCS)[1] and 99 cases (65 male, mean age 67 yrs) from Ninewells Hospital, Dundee and Perth Royal Infirmary collected between 1997 and 2000[2]. Cases were oversampled for familial CRC and/or early age at diagnosis. Population controls with no personal history of cancer were ascertained from four cohorts including 8,533 (3,599 male, mean age 55.4 yrs) - from Generation Scotland-Scottish Family Health Study[3,4]; 513 (211 male, mean age 79 yrs) and 1,004 (508 male, mean age 70 yrs) from the Lothian Birth Cohorts 1921 and 1936[5], respectively; and 262 Dundee controls (132 male) were recruited through the same General Practice surgeries as cases or from spouses/friends of cases [2].

The English series comprised 1,344 cases (807 male, mean age 60yrs), enriched for familial CRC, from the National Study of Colorectal Cancer Genetics (NSCCG)[6], 1,547 cases (852 male, mean age 61yrs) from the Colorectal Tumour Gene Identification Consortium (CORGI) [7] study or QUASAR2 clinical trial of adjuvant bevacizumab, 1,667 cases (981 male, mean age 67yrs)

from cases from Yorkshire (Leeds General Infirmary and St James's Hospital, Harrogate District Hospital and York District Hospital) recruited between 1997 and 2000[2]. English cancer free controls comprised 5,964 individuals (3,350 male) from the UK 1958 Birth Cohort [8,9], 4,564 (2,056 male) from the Oxford Biobank, 648 healthy controls (301 male) from Leeds recruited via the same General Practice surgeries as Leeds cases or spouses/friends of cases and 73 controls (45 male) from York [2].

The Kiel series comprised 192 cases aged <50 years (92 male, mean age 44yrs). All cases were of German descent defined by parental birthplace and self-reported ethnicity. [10] None of the cases were Amsterdam or Bethesda positive or had a past history of inflammatory bowel disease. Population controls (N=1,008; 562 male, mean age 56 years, range 42-67) free of cancer at time of ascertainment were from POPGEN registry in Northern Germany [11]. The Heidelberg series included 92 cases (mean age at diagnosis 42 years) with familial or early-onset microsatellite stable (MS) CRC collected as part of the German HNPCC Consortium; all were Caucasian. The controls were 92 healthy blood donors frequency matched to cases by age and sex.

The Leiden series comprised 384 (190 males) patients with familial or early-onset CRC from the south-western part of the Netherlands, were found to have microsatellite-stable (MS) tumours. 384 controls were blood donors from the southwest region of the Netherlands. The Groningen series comprised 96 patients (36 male, mean age) who developed early-onset MS CRC. Population controls (n=96) had no-family or personal history of CRC or adenomas (46 male).

The Portuguese series comprised 200 patients with early-onset or familial CRC (109 male, mean age at diagnosis 49; SD±8.7). Fifty-four patients were Bethesda or Amsterdam criteria for Lynch syndrome but were mutation negative. Controls (109 male, mean age 49; SD±8.7) were blood donors from the Portuguese Oncology Institute of Porto.

The Spanish series were ascertained from the EPICOLON cohort: the 300 (194 male) cases that had tested negative for Lynch syndrome and were selected by (i) family history of CRC in first or second degree relatives (108 cases, aged 43-88 years), (ii) sporadic CRC diagnosed at under 60yrs (age-at diagnosis range 26-60yrs, 74 cases) , (iii) first degree relatives (FDR) with other Lynch tumours (28 cases, age at diagnosis range 63-71yrs) or , (iv) other (age-at diagnosis 71-73yrs,n=90). Controls comprised 300 cancer-free individuals from the Spanish population (163 male, aged 41-95yrs).

***Genotyping Quality Control for Exome Array Analysis***

Variants were excluded from analysis if call rate was < 99%, the variant deviated significantly from Hardy-Weinberg equilibrium (P<0.001) or was monomorphic in the studied population. We further examined clustering by visually assessing all top variants using Illumina Genome Studio and excluded probable miscalled SNPs through visual inspection of genotyping clusters. Sample exclusions were: genotyping success rate < 99%, abnormal heterozygosity (>3 standard deviations from mean); sex discrepancies between predicted and reposted gender (threshold of X chromosome homozygosity <30% for females and >70% for males), evidence of non- European ancestry using STRUCTURE analysis[12] or evidence of being population outlier based on principal component analysis (PCA) using EIGENSTRAT[13] or ACTA [14]. We also excluded unexpected duplicated samples and first degree relatives based on identity-by-descent (IBD) values. Further detail of sample and probe exclusion is detailed in Supplementary Table 2. Current study includes samples genotyped using different genotyping arrays and version of Illumina Exome array.  We addressed this issue by performing comparison of minor allele frequencies and genotyping rates between different arrays and versions of arrays[15] (Supplementary Figures 1, 2 and 3). We excluded all variants that showed high deviation in frequencies and call rates (defined as abs(diff(array1, array2)) > 0.10) between arrays/version of arrays. It excluded additional 53,639 probes . Clustering of cases

and controls by study and overall as well samples genotyped using different version of arrays were checked using principal component analyses as implemented in ACTA (Supplementary Figures 4 and 5)[14]. Genotyping quality control was evaluated using duplicate DNA samples in assays. 165 samples were genotyped on both the HumanExome-12v1.0 and HumanExome-12v1.1 arrays and genotype concordance was > 97% per pair, concordance rate was >99% for 2980 individuals overlapping between VQ58 study and England. Concordance of exome array genotypes with exome sequencing data performed on 14 samples was 99.7% for 6,451 sites. All variants are mapped and presented according to human reference sequence build 37 (GRCh37.p13).

***Additional GWAS series***

To enhance our power we made use of previously published GWASs[16,17], thus providing exome array variant data on 3,549 cases and 3,698 controls from UK1 and UK2 studies, 3,158 cases and 3,073 controls from Scotland Phase1, Scotland Phase2 and Scotland Phase3, and 1,794 cases and 2,686 controls from the VQ58 study[16,18]. Study details, details of genotyping, quality control procedures, sample and SNPs exclusion for these GWAS-focussed studies have been published previously[16]. Briefly, UK1[17] comprised 890 cases with CRC ascertained through (CoRGI) consortium. The 900 controls were spouses or partners unaffected by cancer and without a personal family history (to second-degree relative level) of colorectal neoplasia. UK2 (NSCCG) consisted of 2,659 cases ascertained through the Institute of Cancer Research /Royal Marsden Hospital NHS Trust (RMHNHST) from 1999 onwards – The NSCCG[6] and The Royal Marsden Hospital Trust/Institute of Cancer Research Family History and DNA Registry. The 2,798 controls were the cancer-free spouses or unrelated friends of cancer patients. Scotland Phase 1 (COGS)[17] comprised 973 early-onset CRC cases and 998 cancer-free population controls. An additional 178 individuals from Scotland Phase 3 study were recruited as part of SOCCS/COGS studies [18] and genotyped using Illumina HumanOmni5-4v1

array. Scotland Phase 2 was based on an additional 2,007 cases from SOCCS and 2,075 controls. VQ58 comprised 1,794 CRC cases from the VICTOR [19] and QUASAR2 (www.octo-oxford.org.uk/alltrials/trials/q2.html) trials. Controls were 2,686 individuals genotyped by the Wellcome Trust Case – Control Consortium 2 (WTCCC2) 1958 birth cohort [8]. Controls from the WTCCC2 1958 birth cohort were split and used as controls for cases from the Exome-Wide association study in UK and for cases from VICTOR/QUASAR2 trials.

VQ, UK1, Scotland Phase 1 cohorts were genotyped using Illumina Hap300, Hap240S, Hap370 or Hap550 arrays. 1958BC genotyping was performed as part of the WTCCC2 study on Hap1.2M-Duo Custom arrays. Scotland Phase 2 and UK2 samples were genotyped using Illumina Infinium-iSelect and GoldenGate arrays for a common set of 43,140 SNPs [16]. We excluded all expected duplicates between Scotland, UK and VQ58 GWAS studies and exome-array studies from Scotland and England, as well as the 1958 birth cohort controls. IBD analysis was performed across all samples and any further, unexpected, duplicates and first-degree relatives were excluded (Supplementary Table 3). After quality control procedures we ended up with ~10,000 exome array variants on 3,033 cases and 3,690 controls from UK1 and UK2 studies, 556 cases and 2,997 controls from Scotland Phase1, Scotland Phase2 and Scotland Phase3, and 949 cases and 538 controls from the VQ58 study[16,18].

***Heritability analyses.***

To estimate the contribution of exome-wide significant SNPs to the variance explained, we used the method proposed by Yang *et al*.[20,21], and implemented in Genome-Wide Complex Trait Analysis (GCTA) software[22]. The genetic relationship matrix was estimated from the exome array data using (1) all SNPs significant at the exome-wide level in our analysis and (2) 5 newly described variants significant at the exome-wide level. We used restricted maximum likelihood (REML), the default option for GCTA, to fit the appropriate variance components

model. The final estimate of heritability on the underlying liability scale assumed that the lifetime risk of colorectal cancer was 0.06 [23].

**Reference:**

1 Theodoratou, E. *et al.* Modification of the inverse association between dietary vitamin D intake and colorectal cancer risk by a FokI variant supports a chemoprotective action of Vitamin D intake mediated through VDR binding. *Int J Cancer* **123**, 2170-2179, doi:10.1002/ijc.23769 (2008).

2 Barrett, J. H. *et al.* Investigation of interaction between N-acetyltransferase 2 and heterocyclic amines as potential risk factors for colorectal cancer. *Carcinogenesis* **24**, 275-282 (2003).

3 Smith, B. H. *et al.* Cohort Profile: Generation Scotland: Scottish Family Health Study (GS:SFHS). The study, its participants and their potential for genetic research on health and illness. *Int J Epidemiol* **42**, 689-700, doi:10.1093/ije/dys084 (2013).

4 Smith, B. H. *et al.* Generation Scotland: the Scottish Family Health Study; a new resource for researching genes and heritability. *BMC Med Genet* **7**, 74, doi:10.1186/1471-2350-7-74 (2006).

5 Deary, I. J., Gow, A. J., Pattie, A. & Starr, J. M. Cohort profile: the Lothian Birth Cohorts of 1921 and 1936. *Int J Epidemiol* **41**, 1576-1584, doi:10.1093/ije/dyr197 (2012).

6 Penegar, S. *et al.* National study of colorectal cancer genetics. *Br J Cancer* **97**, 1305-1309, doi:10.1038/sj.bjc.6603997 (2007).

7 Kemp, Z. *et al.* Evidence for a colorectal cancer susceptibility locus on chromosome 3q21-q24 from a high-density SNP genome-wide linkage scan. *Hum Mol Genet* **15**, 2903-2910, doi:10.1093/hmg/ddl231 (2006).

8 Power, C. & Elliott, J. Cohort profile: 1958 British birth cohort (National Child Development Study). *Int J Epidemiol* **35**, 34-41, doi:10.1093/ije/dyi183 (2006).

9 Power, C., Jefferis, B. J., Manor, O. & Hertzman, C. The influence of birth weight and socioeconomic position on cognitive development: Does the early home and learning environment modify their effects? *J Pediatr* **148**, 54-61, doi:10.1016/j.jpeds.2005.07.028 (2006).

10 Castro, F. A. *et al.* TLR-3 polymorphism is an independent prognostic marker for stage II colorectal cancer. *Eur J Cancer* **47**, 1203-1210, doi:10.1016/j.ejca.2010.12.011 (2011).

11 Krawczak, M. *et al.* PopGen: population-based recruitment of patients and controls for the analysis of complex genotype-phenotype relationships. *Community Genet* **9**, 55-61, doi:10.1159/000090694 (2006).

12 Falush, D., Stephens, M. & Pritchard, J. K. Inference of population structure using multilocus genotype data: linked loci and correlated allele frequencies. *Genetics* **164**, 1567-1587 (2003).

13 Price, A. L. *et al.* Principal components analysis corrects for stratification in genome-wide association studies. *Nat Genet* **38**, 904-909, doi:10.1038/ng1847 (2006).

14 Gray, A., Stewart, I. & Tenesa, A. Advanced complex trait analysis. *Bioinformatics* **28**, 3134-3136, doi:10.1093/bioinformatics/bts571 (2012).

15 Guo, Y. *et al.* Illumina human exome genotyping array clustering and quality control. *Nat Protoc* **9**, 2643-2662, doi:10.1038/nprot.2014.174 (2014).

16 Dunlop, M. G. *et al.* Common variation near CDKN1A, POLD3 and SHROOM2 influences colorectal cancer risk. *Nat Genet* **44**, 770-776, doi:10.1038/ng.2293 (2012).

17 Houlston, R. S. *et al.* Meta-analysis of three genome-wide association studies identifies susceptibility loci for colorectal cancer at 1q41, 3q26.2, 12q13.13 and 20q13.33. *Nat Genet* **42**, 973-977, doi:10.1038/ng.670 (2010).

18 Tenesa, A. *et al.* Genome-wide association scan identifies a colorectal cancer susceptibility locus on 11q23 and replicates risk loci at 8q24 and 18q21. *Nat Genet* **40**, 631-637, doi:10.1038/ng.133 (2008).

19 Midgley, R. S. *et al.* Phase III randomized trial assessing rofecoxib in the adjuvant setting of colorectal cancer: final results of the VICTOR trial. *J Clin Oncol* **28**, 4575-4580, doi:10.1200/JCO.2010.29.6244 (2010).

20 Yang, J. *et al.* Common SNPs explain a large proportion of the heritability for human height. *Nat Genet* **42**, 565-569, doi:10.1038/ng.608 (2010).

21 Lee, S. H., Wray, N. R., Goddard, M. E. & Visscher, P. M. Estimating missing heritability for disease from genome-wide association studies. *Am J Hum Genet* **88**, 294-305, doi:10.1016/j.ajhg.2011.02.002 (2011).

22 Yang, J., Lee, S. H., Goddard, M. E. & Visscher, P. M. GCTA: a tool for genome-wide complex trait analysis. *Am J Hum Genet* **88**, 76-82, doi:10.1016/j.ajhg.2010.11.011 (2011).

23 Sasieni, P. D., Shelton, J., Ormiston-Smith, N., Thomson, C. S. & Silcocks, P. B. What is the lifetime risk of developing cancer?: the effect of adjusting for multiple primaries. *Br J Cancer* **105**, 460-465, doi:10.1038/bjc.2011.250 (2011).

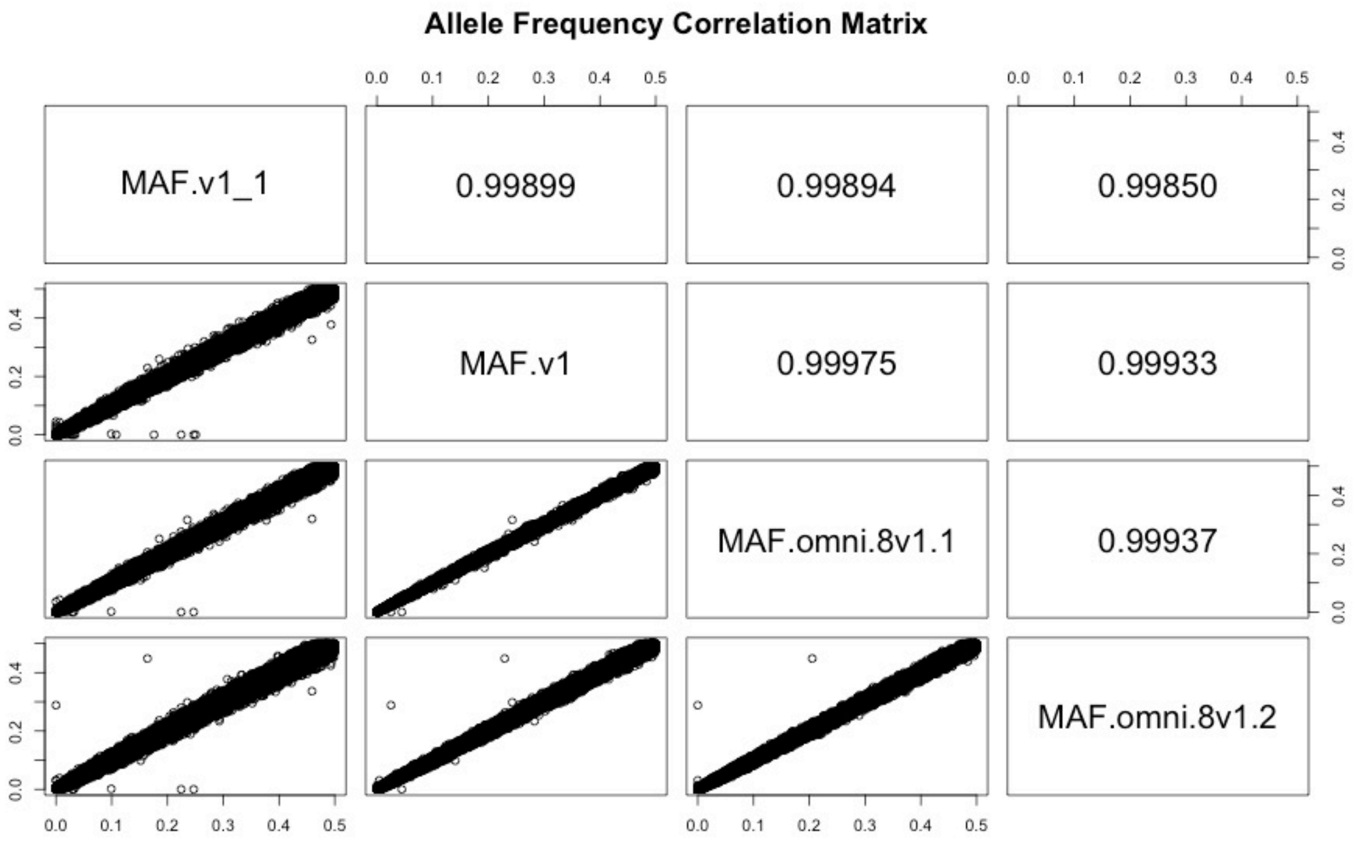


**Supplementary Figure 1**: Correlation matrix of allele frequency consistency between Infinium Human Exome BeadChip 12v1.0, 12v1.1 versions of arrays and OmniExpressExome BeadChip 8v1.1 / 8v1.2. MAF.v1_1 – Exome BeadChio 12v1.1 , MAF.v1 – ExomeBeadChip 12.v1.0 , MAF.omni .8v1.1 – OmniExpressExome BeadChip 8v1.1, MAF.omn.8v1.2 - OmniExpressExome BeadChip . Pearson moment correlation was calculated for each pair of comparison

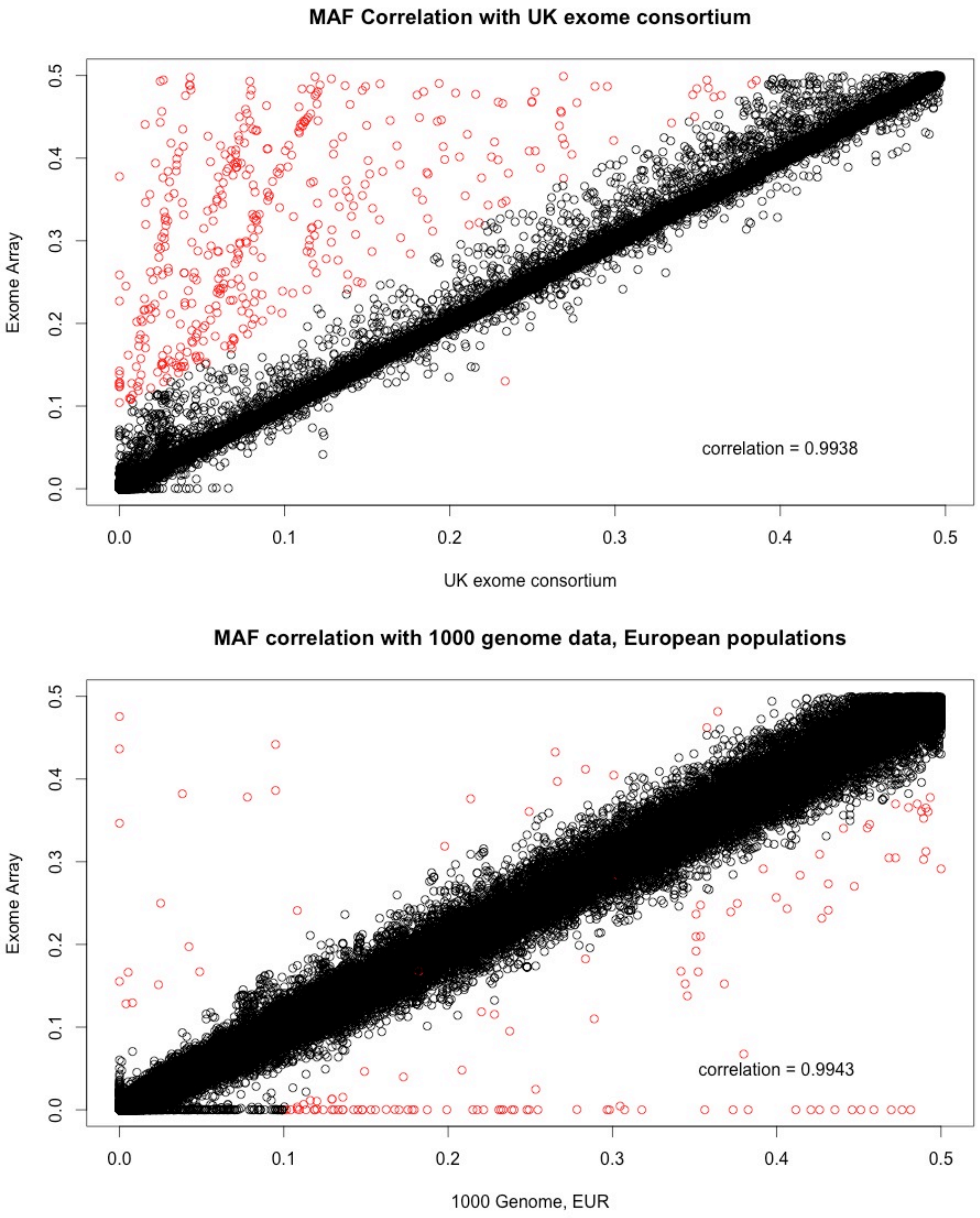


**Supplementary Figure 2** Correlation of MAF between exome array project (all studies and arrays combined) and (A) frequency of Exome array variants from UK exome consortium and (B) overlapping variants in 1000 Genome data. Frequencies in UK exome consortium are calculated using 55,726 European individuals from UK (unpublished data), This set includes control individuals from Oxford BioBank, 1958 birth cohort, as well as 1843 cases from Scotland and 1209 cases from England . MAF in 1000 Genome data was calculated using information on 379 individuals of European ancestry. Correlation between allele frequencies was estimated using Pearson product-moment correlation coefficient

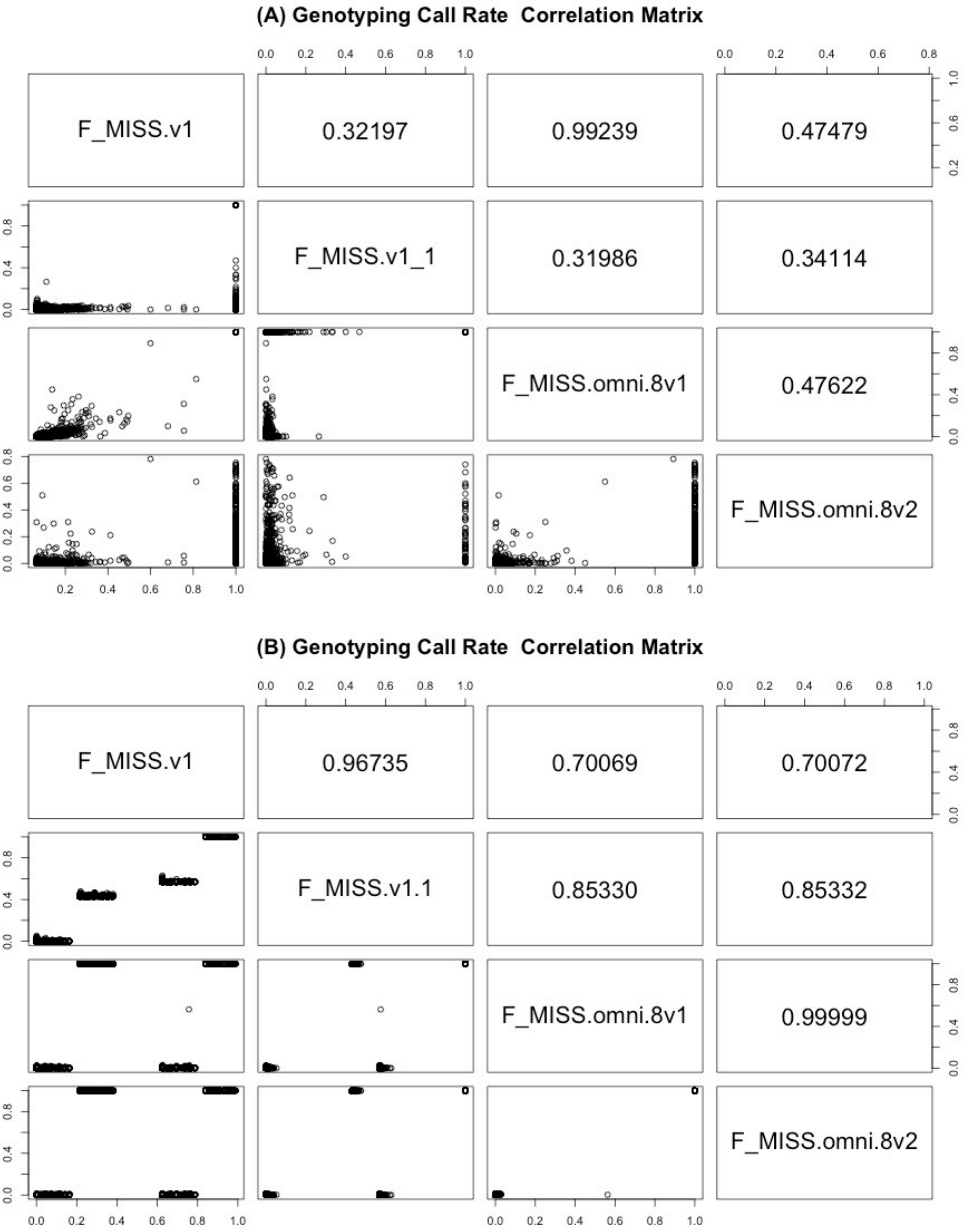


**Supplementary Figure 3** Correlation matrix of genotyping missing rate consistency (A) prior and (B) post quality control procedures between Infinium Human Exome BeadChip 12v1.0, 12v1.1 versions of arrays and OmniExpressExome BeadChip 8v1.1 / 8v1.2.

MAF.v1_1 – Exome BeadChio 12v1.1 , MAF.v1 – ExomeBeadChip 12.v1.0 , MAF.omni .8v1.1 – OmniExpressExome BeadChip 8v1.1, MAF.omn.8v1.2 - OmniExpressExome BeadChip

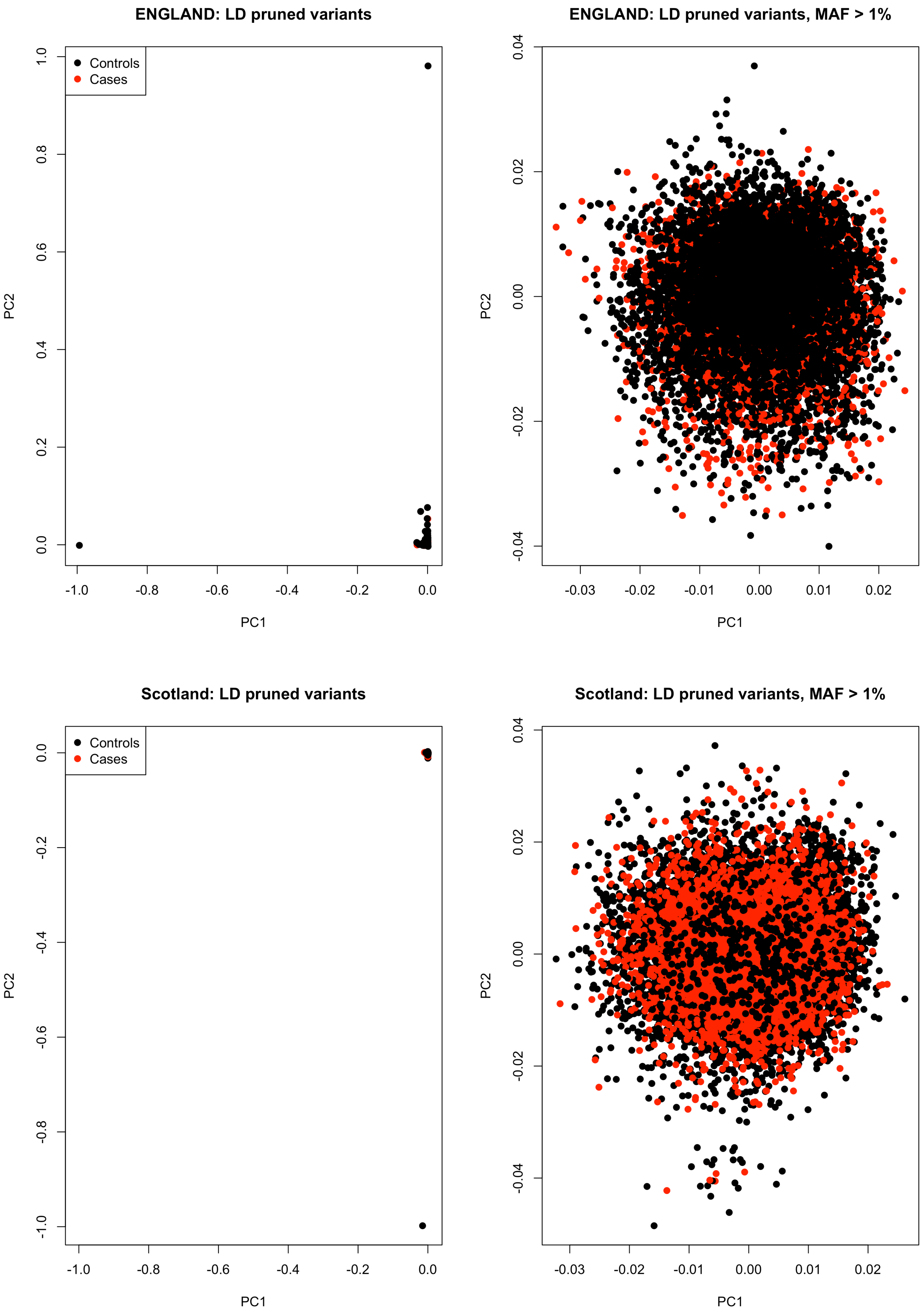
ENGLAND: LD pruned variants
ENGLAND: LD pruned variants, MAF > 1%
Scotland: LD pruned variants
Scotland: LD pruned variants, MAF > 1%
Controls
Cases
PC1
PC2

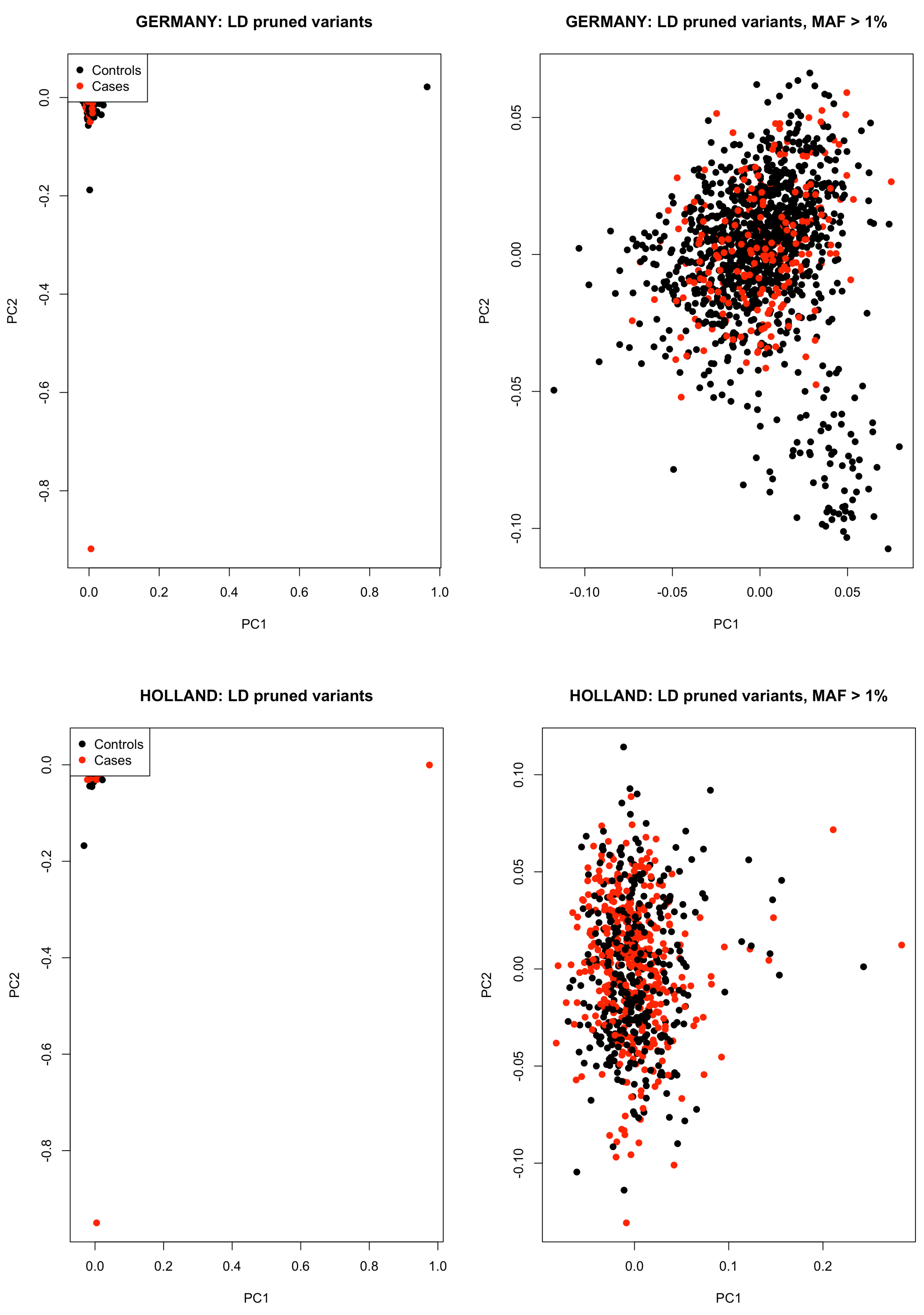
GERMANY: LD pruned variants
Controls
Cases
PC1
PC2
GERMANY: LD pruned variants, MAF > 1%
PC1
PC2
HOLLAND: LD pruned variants
Controls
Cases
PC1
PC2
HOLLAND: LD pruned variants, MAF > 1%
PC1
PC2

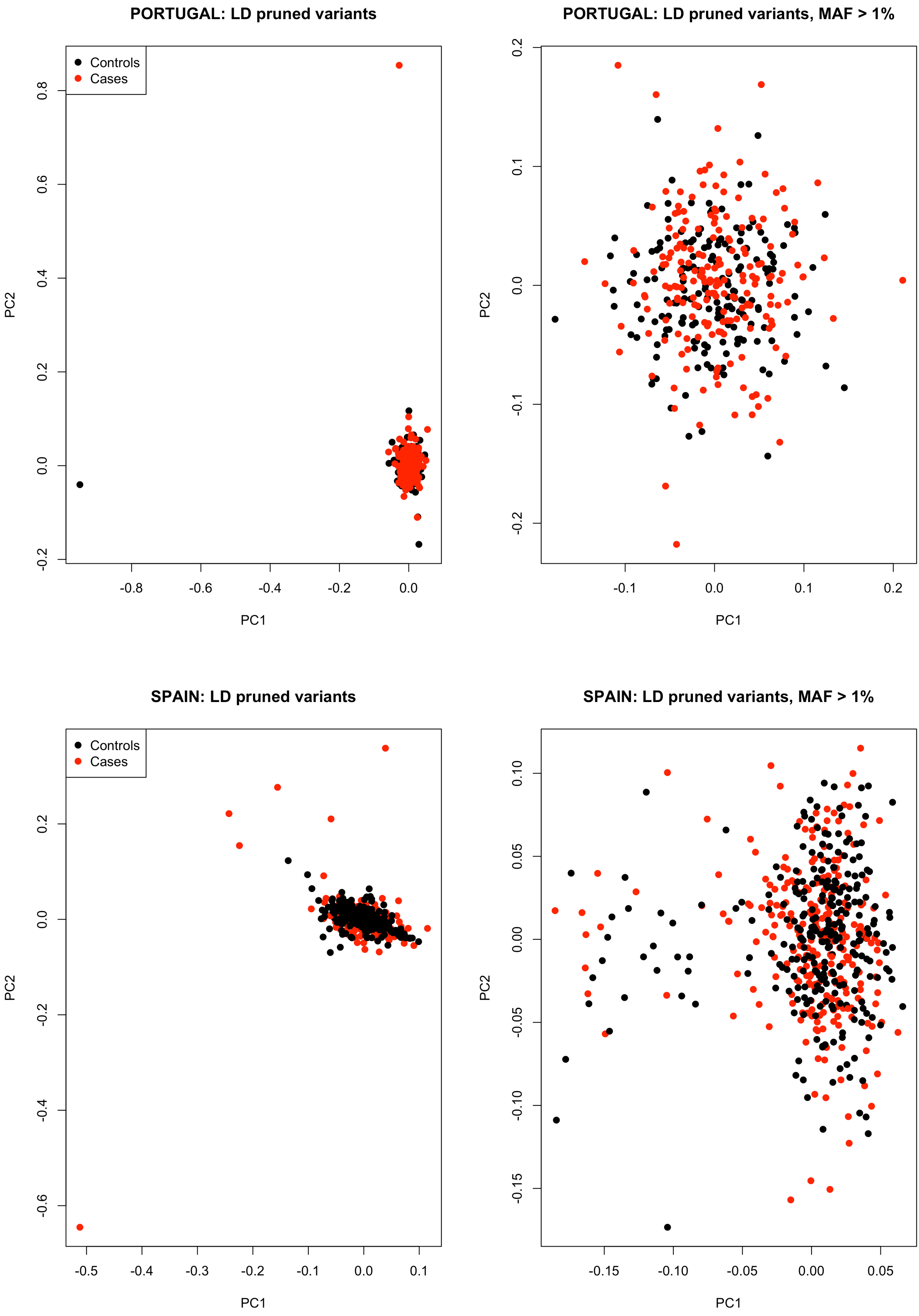
PORTUGAL: LD pruned variants
Controls
Cases
PC1
PC2
PORTUGAL: LD pruned variants, MAF > 1%
PC1
PC2
SPAIN: LD pruned variants
Controls
Cases
PC1
PC2
SPAIN: LD pruned variants, MAF > 1%
PC1
PC2

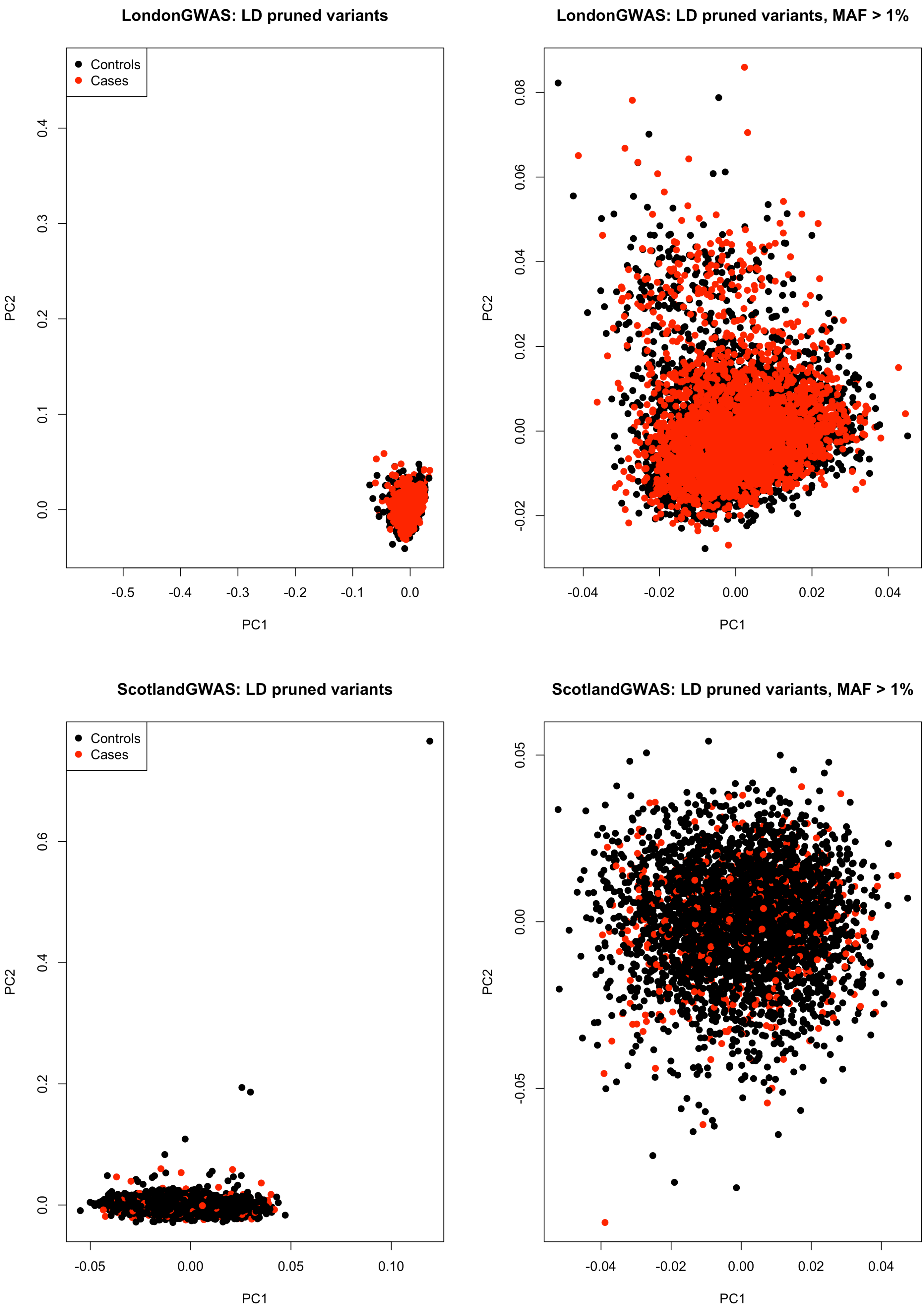
LondonGWAS: LD pruned variants
Controls
Cases
PC1
PC2
LondonGWAS: LD pruned variants, MAF > 1%
PC1
PC2
ScotlandGWAS: LD pruned variants
Controls
Cases
PC1
PC2
ScotlandGWAS: LD pruned variants, MAF > 1%
PC1
PC2

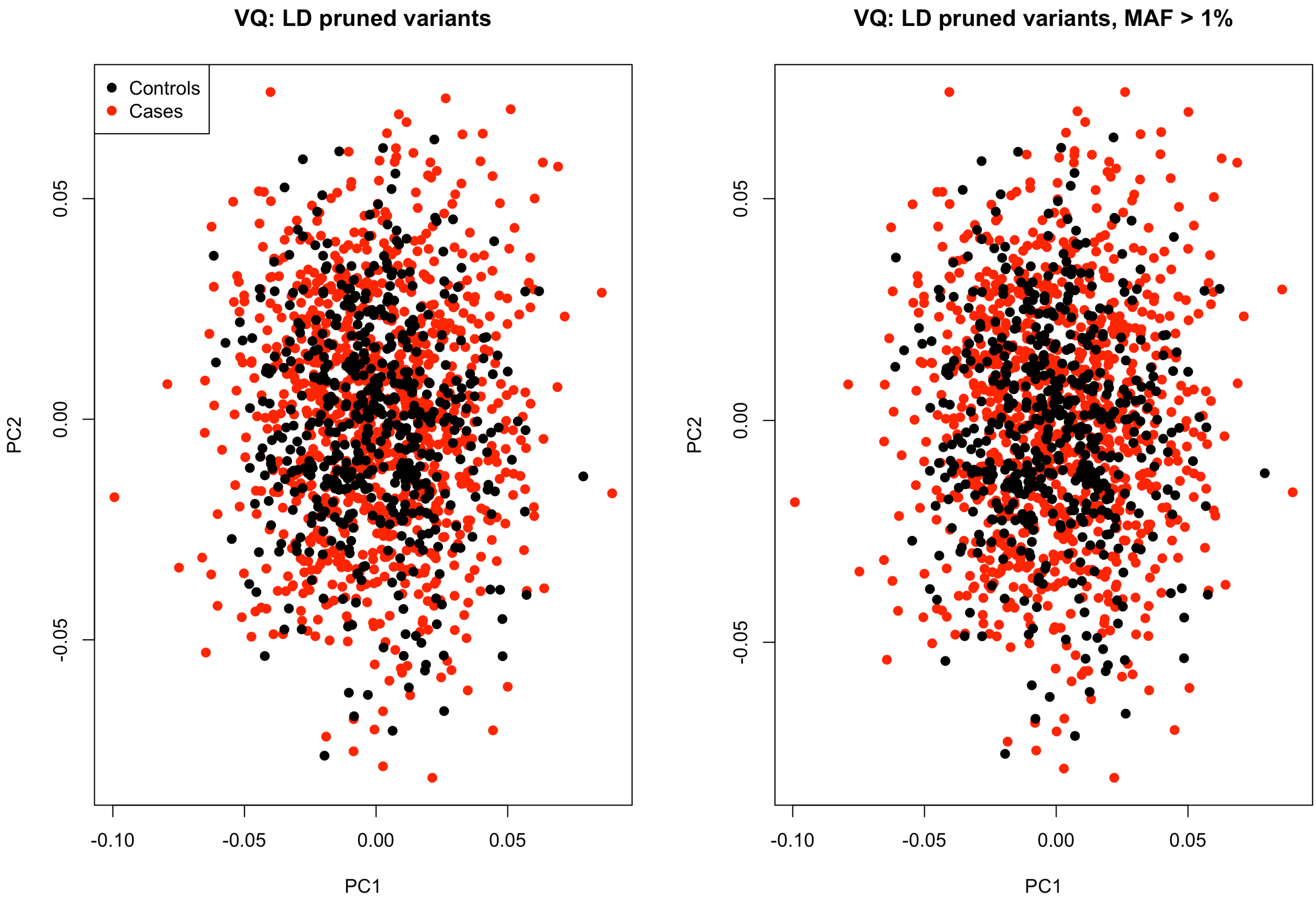


**Supplementary Figure 4**: Identification of non random clustering between cases and controls in different studies using principal component analysis. . LD prunning prior analysis was done in PLINK to exclude highly correlated variants (Parameter used for pruning : --indep-pairwise 100 5 0.1). (A) All variants; (B) All variants with allele frequency above 1%.

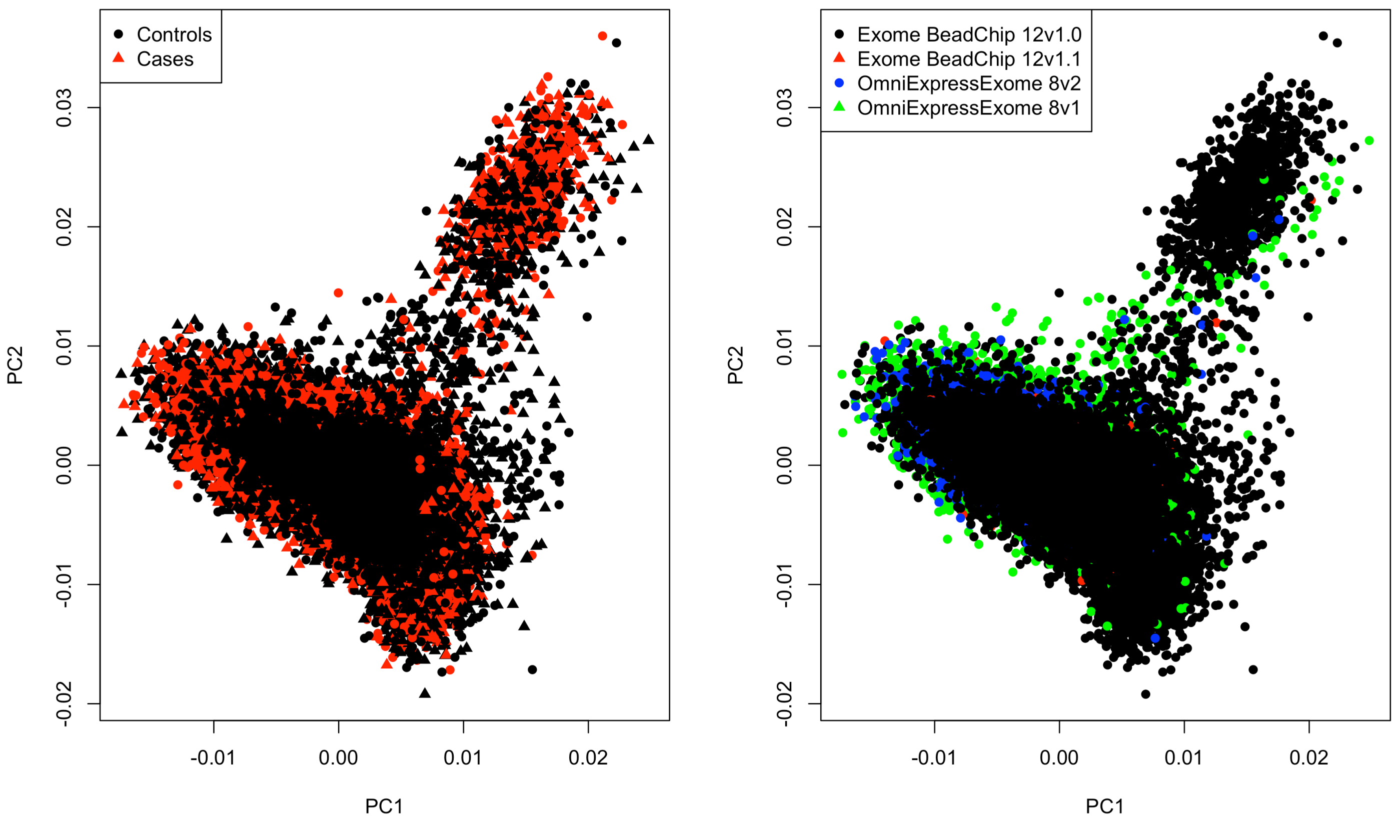


**Supplementary Figure 5**: Identification of non random clustering between all cases and controls (A) and between samples genotyped on different arrays using principal component analysis. LD prunning on the final list of variants after all quality control procedures (MAF>0.001) was done in PLINK to exclude highly correlated variants (Parameter used for pruning : --indep-pairwise 100 5 0.1)

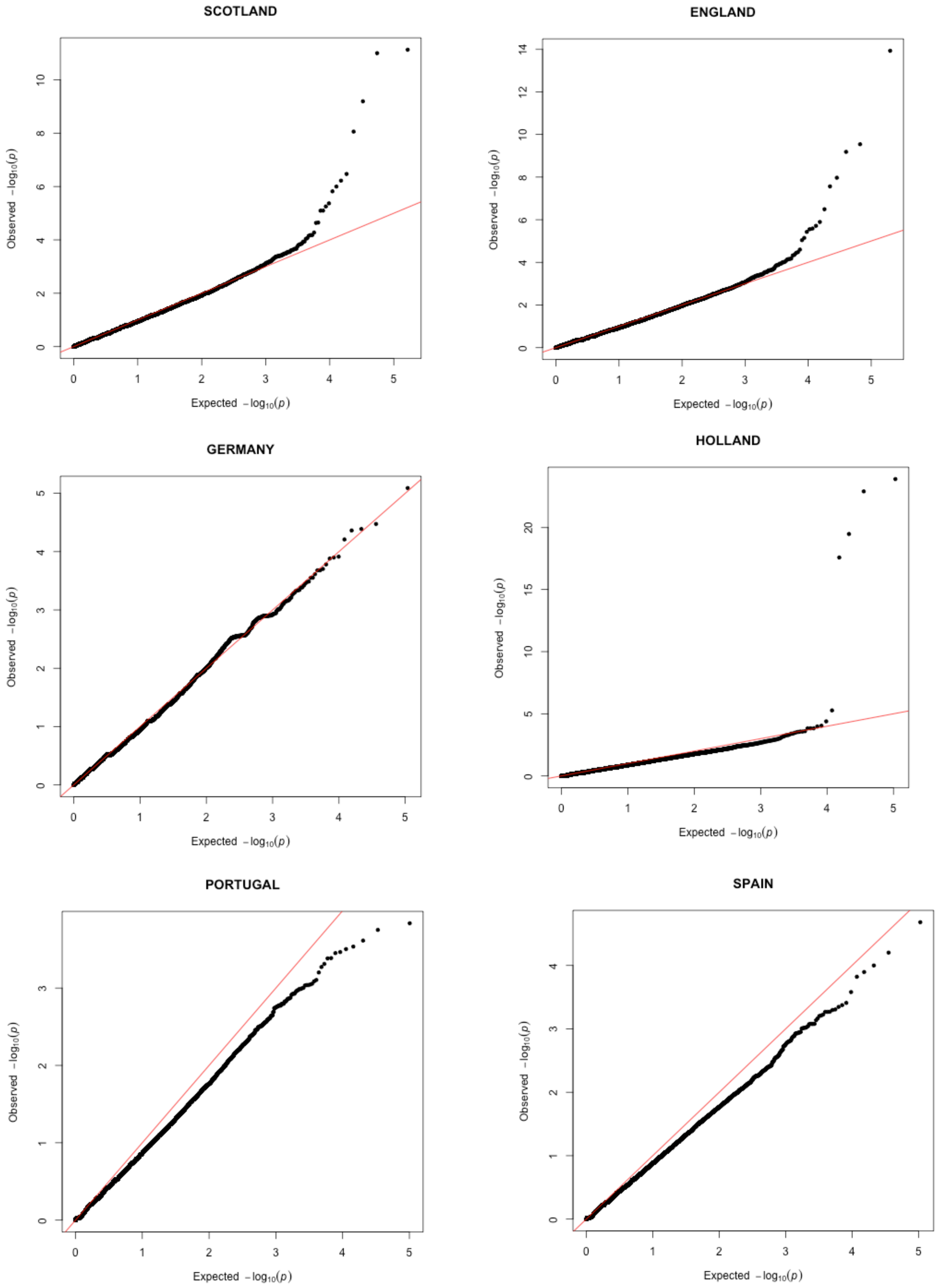


**Supplementary Figure 6**: Quantile-Quantile (Q-Q) plots of observed and expected p values in –log10 scale of association between SNP genotype and colorectal cancer risk in six European studies. (a) Scotland, genomic inflation factor lambda (λ) =0,96; (b) England, λ =0,96; (c) Germany, λ =0,96; (d) Holland, λ =0,88; (e) Portugal, λ =0,84 and (f) Spain, λ =0,85.

A

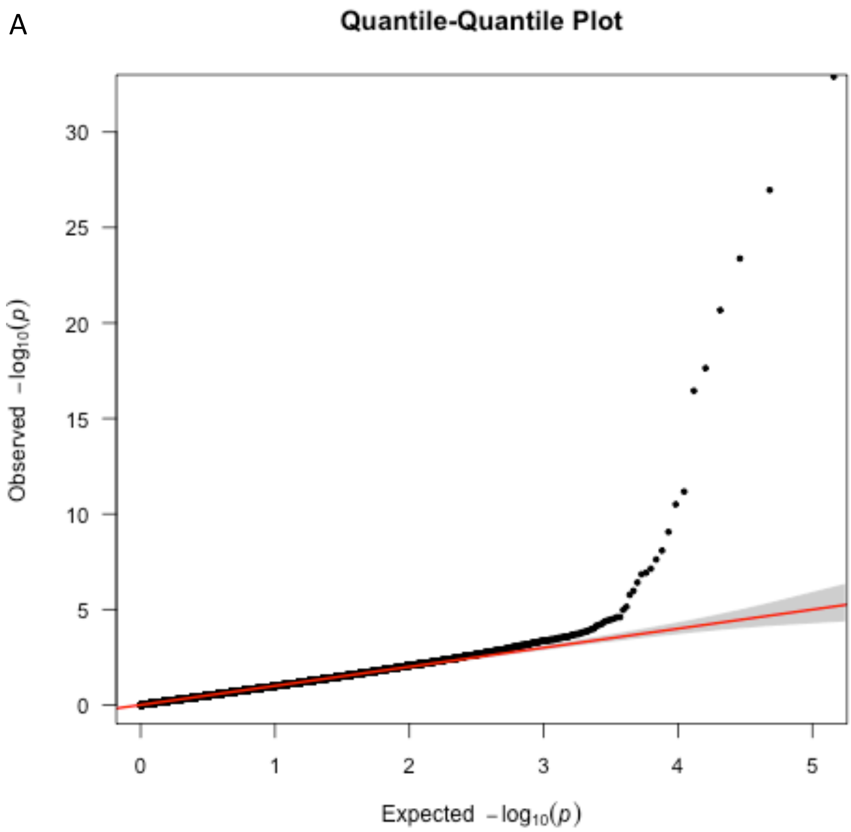


B

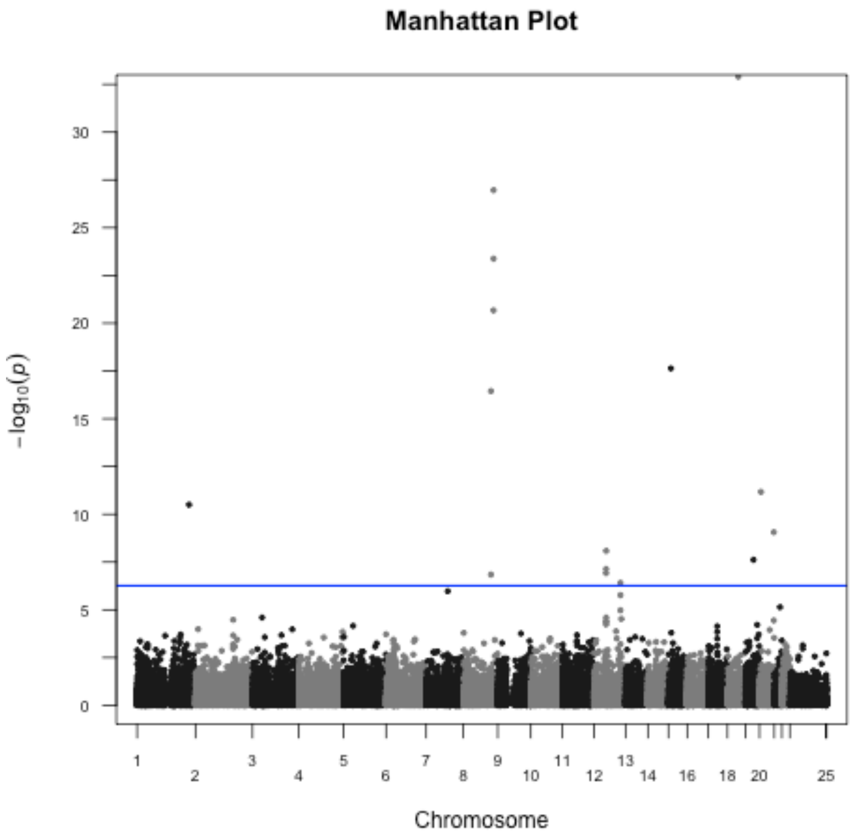


**Supplementary Figure 7**: QQ plot of observed and expected p-values in –log10 scale (A) and Manhattan (B) plots of association between 72,162 non-monomorphic variants and colorectal cancer risk in a meta-analysis comprising of 12638 cases and 29048 controls of European origin.

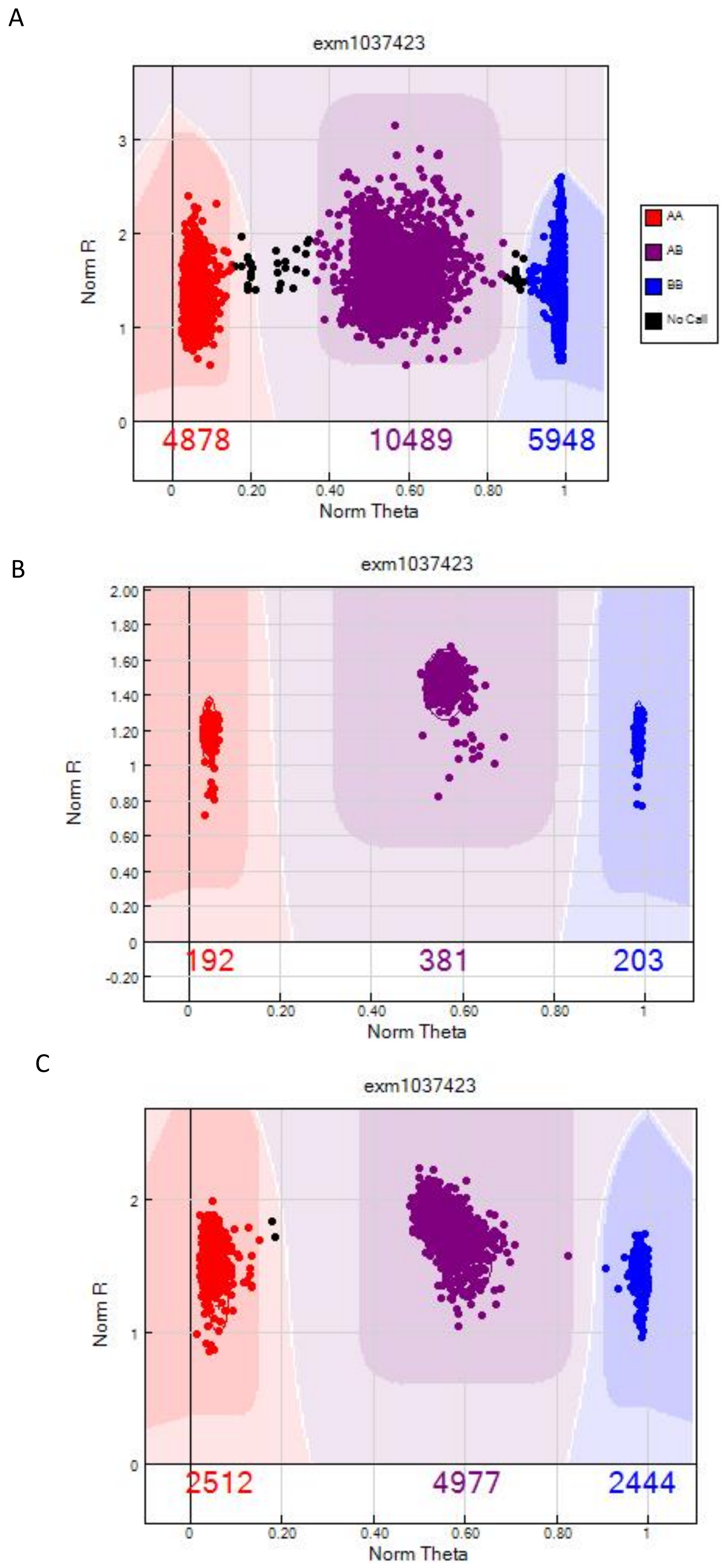


**Supplementary Figure 8**: Cluster plots for rs3184504 (*SH2B3*, 12q24) variant in different arrays.
(A) Infinium Human Exome BeadChip 12v1.0, (B) Infinium Human Exome BeadChip 12v1.1 , (C) OmniExpressExome BeadChip 8v1.1

A

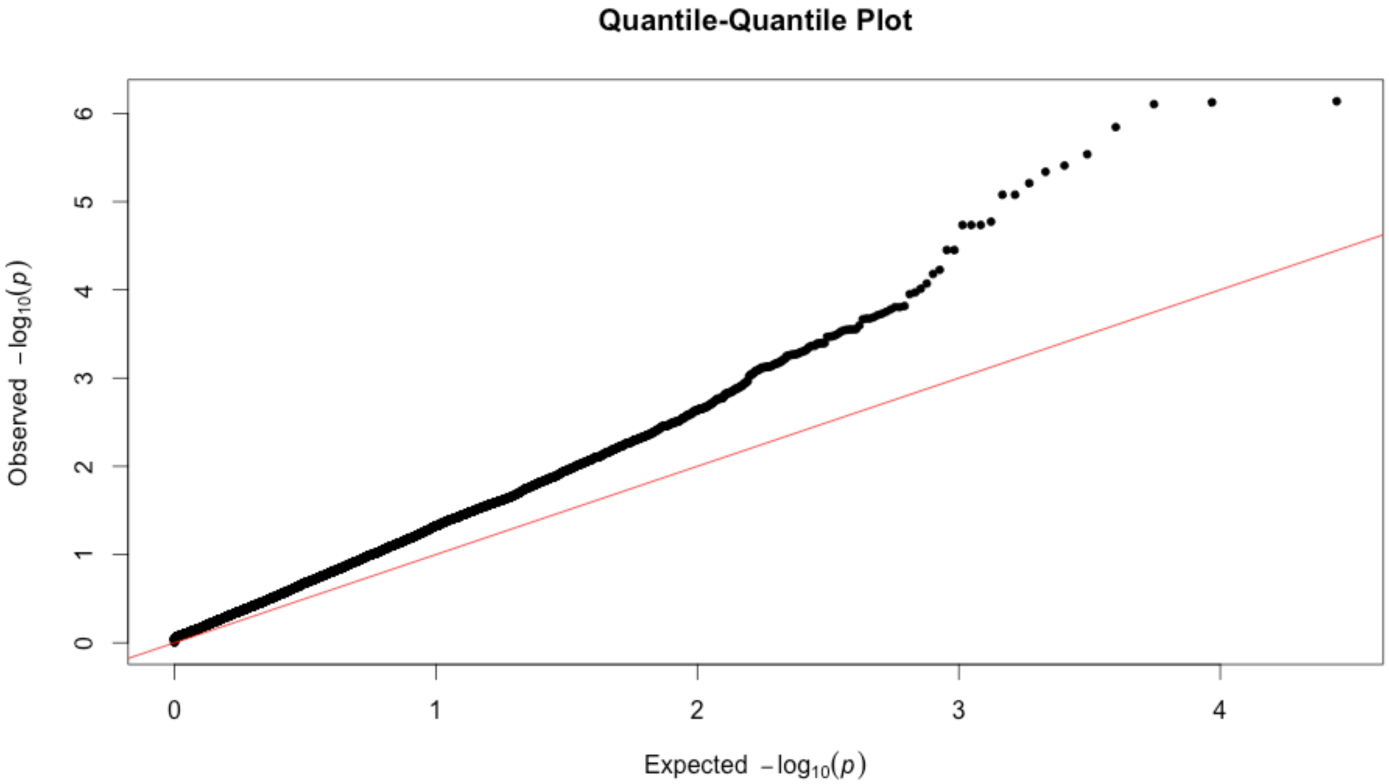


B

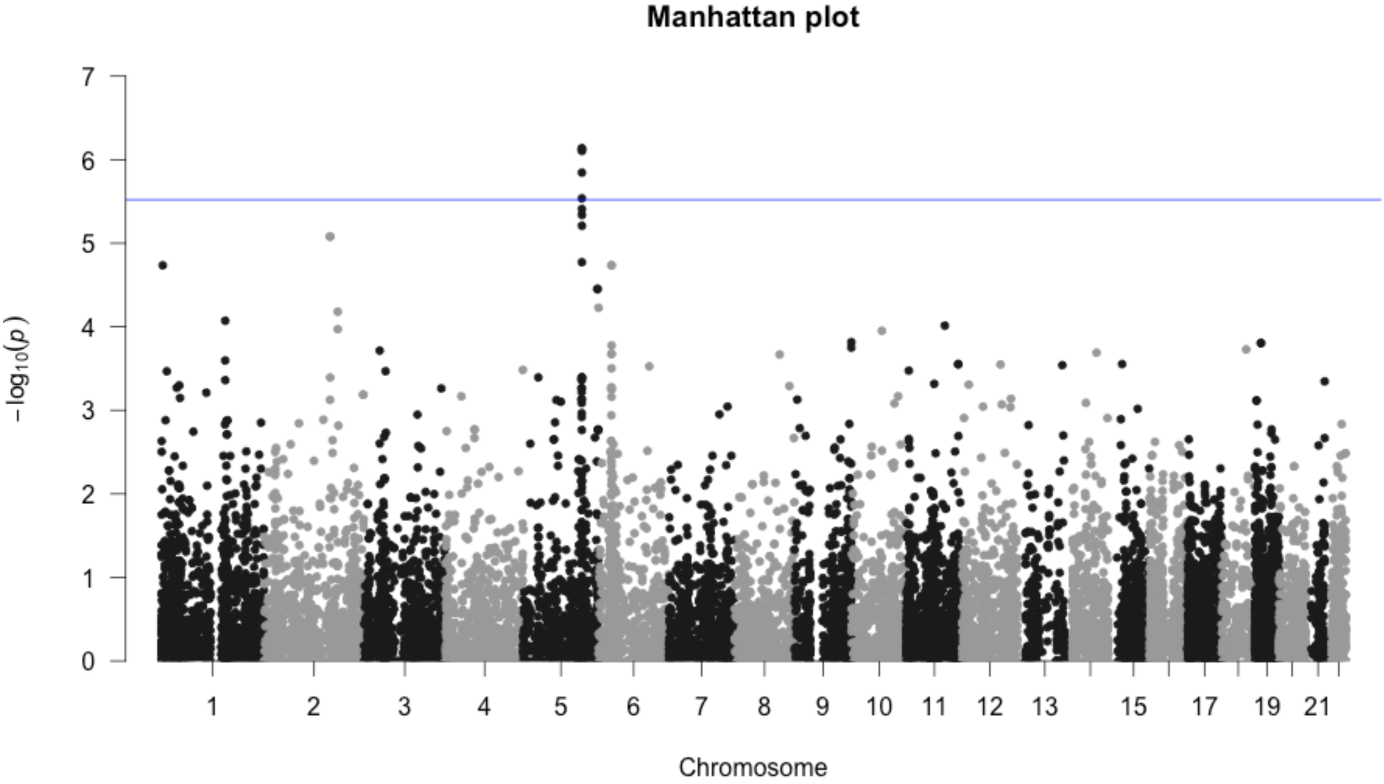


**Supplementary Figure 9**: QQ plot of observed and expected P-values in – $\log_{10}$ scale (A) and Manhattan (B) plots of association between 16,585 genes and colorectal cancer risk in a gene-based meta-analysis comprising of 12638 cases and 29045 controls of European origin.

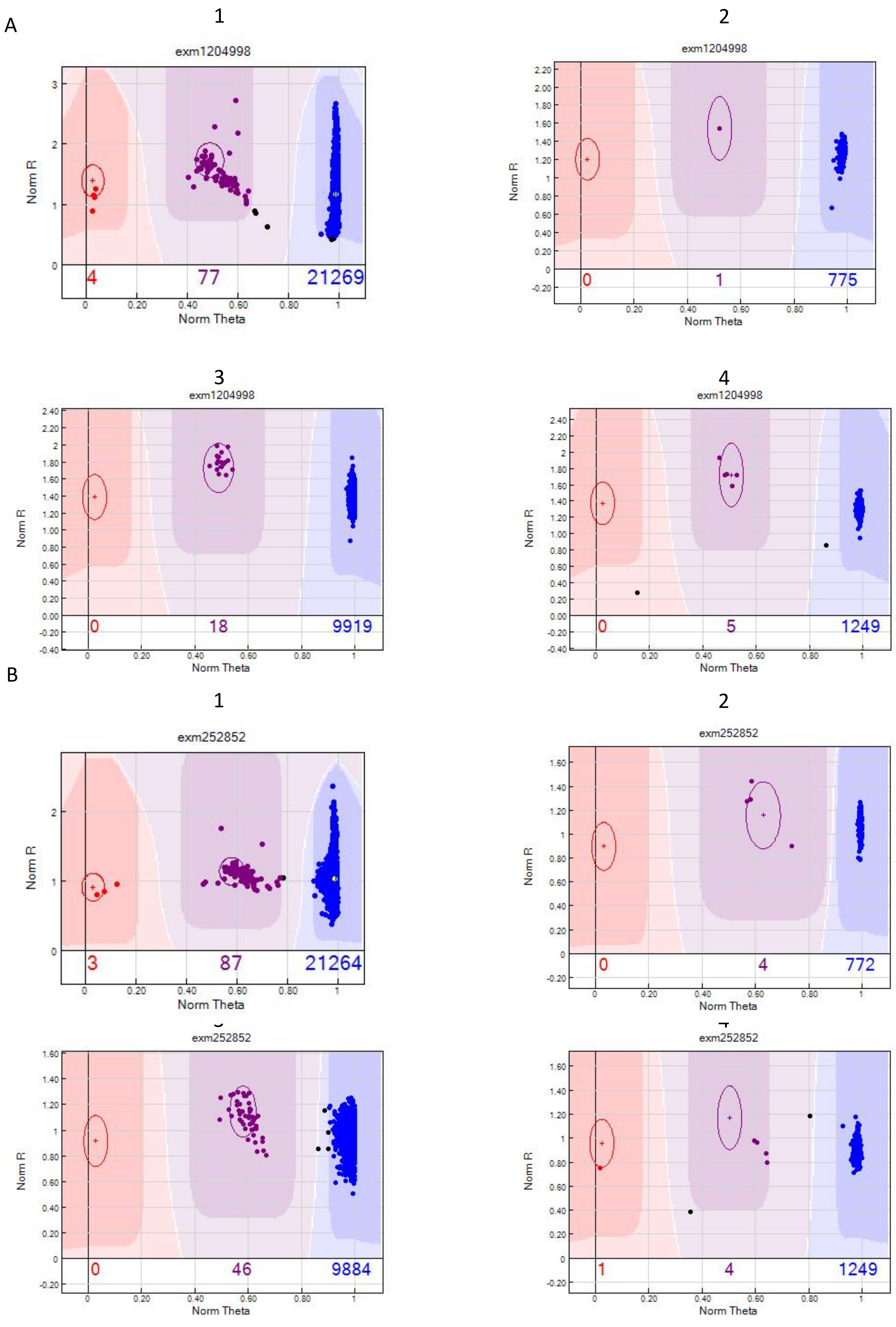


**Supplementary Figure 10**: Cluster plots for rs150766139 (p.Gln90*,*NTHL1*, 16p13.3,exm1204998) and rs61756360 (p.Thr75Ile,PMS1, 2q32.2,exm252852) variants in different arrays.
(1) Infinium Human Exome BeadChip 12v1.0, (2) Infinium Human Exome BeadChip 12v1.1 , (3) OmniExpressExome BeadChip 8v1.1, (4) OmniExpressExome BeadChip 8v1.2.

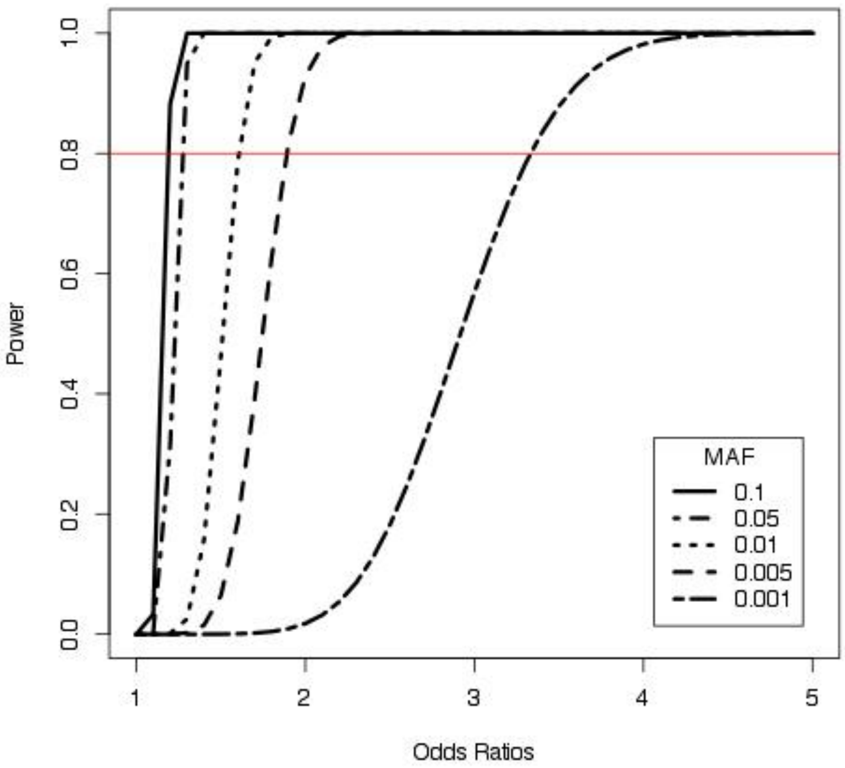


**Supplementary Figure 11:** Power to detect CRC susceptibility variants over different effect size (OR) and for various minor allele frequencies (MAF).

**Supplementary Table 1. Distribution of cases and controls by study.**

| Studies | Cases | Controls | N of variants after QC | N of nonmonomorphic variants contributing to meta-analysis |
|---|---|---|---|---|
| Scotland Exome | 3418 | 9350 | 192460 | 109465 |
| England | 3584 | 10590 | 192460 | 118938 |
| Germany | 247 | 1053 | 192460 | 68005 |
| Holland | 397 | 376 | 192120 | 56527 |
| Spain | 259 | 273 | 192030 | 57351 |
| Portugal | 195 | 178 | 192342 | 53132 |
| **Overall Exome Array** | **8100** | **21820** | **192460** | |
| UK1+UK2 | 3033 | 3690 | 9853 | 9749 |
| Scotland | 556 | 2997 | 8789 | 8626 |
| VQ/58 | 949 | 538 | 7545 | 7545 |
| **Overal Replication** | **4538** | **7225** | | |
| **Overall** | **12638** | **29045** | | 72,162 |

**Supplementary Table 2 . Sample and probe exclusion by study.**

| | England | Scotland | Germany | Holland | Spain | Portugal |
|---|---|---|---|---|---|---|
| **QC on samples** | | | | | | |
| **Pre-QC (cases/controls)** | 4 558/11 249 | 3 616/10 312 | 284/1 100 | 480/480 | 300/300 | 200/200 |
| **Individual QC by study** | 1,191 | | | | | |
| **LeedsYork** | 30 | | | | | |
| **OXBB** | 0 | | | | | |
| **ENGLAND_WALES** | 38 | | | | | |
| | 3 661/10 888 | | | | | |
| Missing rate per person (>0.01) | 9 | 151 | 45 | 115 | 22 | 2 |
| Inbreeding, sample contamination (mean heterozygosity rate ± 3sd/6sd) | 29 | 19 | 0 | 0 | 0 | 0 |
| Population outliers (ACTA and STRUCTURE outliers) | 77 | 105 | 34 | 59 | 32 | 14 |
| Diagnosed with cancer (for population-based controls) | | 184 | 0 | 0 | 0 | 0 |
| Sex discrepencies | 191 | 47 | 0 | 0 | 0 | 0 |
| Other (apendix, adenoma cases, sample swap, the same ID as case and as a control) | | 21 | 0 | 0 | 12 | 0 |
| Between study duplicates, first degree relatives | 69 | 617 | 5 | 14 | 2 | 11 |
| Genotyping duplicates | | 16 | | | | |
| **Post QC (cases/controls)** | 3 584/10 590 | 3 418/9 350 | 247/1 053 | 397/376 | 259/273 | 195/178 |
| **QC on probes** | | | | | | |
| Strand problem | | 14 | | | | |
| deviation from HWE (p<=0.001 in controls) | 914 | 504 | 910 | 1,246 | 165 | 124 |
| Missing rate | 8,969 | 28,571 | 6,052 | 7,335 | 4,166 | 2,959 |
| Missing by case-control status | 11,318 | 19,538 | 3,858 | 511 | 626 | 4 |
| Differences in call rate and frequency between different version of arrays | 33,922 | 6,783 | 44,826 | 46,841 | 50,719 | 52,455 |
| Monomorphic variants (MAF=0) | 73,522 | 91,634 | 124,455 | 135,593 | 134,679 | 139,210 |
| **Final list of non-monomoprhic variants** | 118,938 | 109,465 | 68,005 | 56,527 | 57,351 | 53,132 |

**Supplementary Table 3. Exclusion of between study duplicates for GWAS studies.**

| | UK Phase 1 and 2 | Scotland Phase 1 and 2 and Phase 3* | VQ |
|---|---|---|---|
| **QC on samples** | | | |
| **GWAS QC (ca/co) #** | 3 549/3 698 | 3 158/3 073 | 1 794/2 686 |
| Other (known dominant polyposis syndromes, HNPCC/ Lynch syndrome, adenoma cases) | 294 | 20 | 0 |
| Additional between studies duplicates and relatives † | 230 | 2658 | 2993 |
| **Post-QC (ca/co)** | 3 033/3 690 | 556/2 997 | 949/538 |
| Non monomorphic variants overlapping with Illumina Exome Array | 9749 | 8626 | 7545 |

# Details of QC are presented elsewhere (Dunlop et al., 2012).

* Quality Control for Scotland 3 was done following strandard protocol. 9 individuals overlapping with Scotland 1 and 15 adenoma and non cancer cases were excluded from the analysis.

†Duplicated samples were preferentially removed from these datasets over datasets with available exome-wide data .

**Supplementary Table 4. Top results (p value <0.0001) for the meta-analysis**

| | | | | | | | | Risk Allele Frequency | | | | England | | | | Scotland | | | Holland | | |
|---|---|---|---|---|---|---|---|---|---|---|---|---|---|---|---|---|---|---|---|---|---|
| **SNP** | **rsid** | **CHR** | **BP** | **PPgene** | **A1** | **A2** | **N** | **Cases** | **Controls** | **OR.fixed** | **P.fixed** | **I2** | **OR.fixed** | **Cases** | **Controls** | **OR.fixed** | **Cases** | **Controls** | **OR.fixed** | **Cases** | **Controls** |
| exm-rs4939827 | rs4939827 | 18 | 46453463 | *SMAD7* | A | G | 9 | 0.57 | 0.52 | 1.21 | 1.3E-33 | 0 | 1.24 | 1168/1757/658 | 2859/5257/2472 | 1.22 | 1066/1746/606 | 2520/4665/2157 | 1.12 | 110/206/80 | 99/185/92 |
| exm-rs6983267 | rs6983267 | 8 | 128413305 | | C | A | 9 | 0.56 | 0.52 | 1.19 | 1.1E-27 | 0 | 1.19 | 1127/1798/659 | 2882/5300/2408 | 1.15 | 1033/1716/668 | 2520/4675/2155 | 1.13 | 121/208/68 | 114/176/86 |
| exm-rs7014346 | rs7014346 | 8 | 128424792 | | A | G | 9 | 0.41 | 0.37 | 1.17 | 4.2E-24 | 0 | 1.17 | 611/1738/1234 | 1519/4924/4147 | 1.16 | 572/1665/1180 | 1311/4409/3630 | 1.09 | 74/189/134 | 63/178/135 |
| exm-rs10505477 | rs10505477 | 8 | 128407443 | | A | G | 9 | 0.55 | 0.51 | 1.17 | 2.1E-21 | 0 | 1.19 | 1087/1808/689 | 2777/5322/2491 | 1.14 | 1006/1705/706 | 2456/4668/2225 | 1.08 | 116/202/79 | 109/178/89 |
| exm-rs4779584 | rs4779584 | 15 | 32994756 | | A | G | 9 | 0.21 | 0.19 | 1.19 | 2.3E-18 | 0 | 1.15 | 156/1220/2208 | 386/3277/6926 | 1.16 | 165/1094/2159 | 285/2887/6178 | 1.41 | 19/130/247 | 7/107/262 |
| exm-rs16892766 | rs16892766 | 8 | 117630683 | | C | A | 9 | 0.10 | 0.08 | 1.26 | 3.6E-17 | 0 | 1.25 | 37/629/2918 | 64/1571/8955 | 1.25 | 29/635/2754 | 63/1426/7861 | 1.05 | 4/69/324 | 3/64/309 |
| exm-rs961253 | rs961253 | 20 | 6404281 | | A | C | 9 | 0.39 | 0.36 | 1.12 | 6.8E-12 | 0 | 1.12 | 526/1715/1342 | 1379/4865/4346 | 1.10 | 515/1622/1281 | 1269/4307/3774 | 1.08 | 77/191/129 | 63/187/126 |
| exm-rs6687758 | rs6687758 | 1 | 222164948 | | G | A | 9 | 0.22 | 0.20 | 1.14 | 3.2E-11 | 0 | 1.17 | 177/1224/2181 | 425/3249/6915 | 1.11 | 157/1153/2107 | 379/2927/6027 | 1.19 | 17/141/239 | 11/123/242 |
| exm-rs4925386 | rs4925386 | 20 | 60921044 | *LAMA5* | G | A | 9 | 0.71 | 0.68 | 1.11 | 8.7E-10 | 0 | 1.09 | 1753/1510/320 | 4965/4511/1113 | 1.17 | 1741/1403/273 | 4352/4065/932 | 1.15 | 206/159/32 | 182/155/39 |
| exm1002721 | rs1129406 | 12 | 51203371 | *ATF1* | A | G | 6 | 0.43 | 0.40 | 1.11 | 8.3E-09 | 14 | 1.14 | 666/1762/1156 | 1662/5163/3765 | 1.10 | 609/1726/1083 | 1566/4483/3300 | 1.03 | 67/183/147 | 63/168/145 |
| exm-rs10411210 | rs10411210 | 19 | 33532300 | *RHPN2* | G | A | 9 | 0.92 | 0.91 | 1.18 | 2.4E-08 | 23.9 | 1.12 | 3041/522/20 | 8820/1694/76 | 1.20 | 2933/469/16 | 7799/1484/66 | 1.02 | 347/46/4 | 328/44/4 |
| exm1002434 | rs12303082 | 12 | 50754563 | *FAM186A* | A | C | 9 | 0.37 | 0.35 | 1.09 | 7.4E-08 | 9.15 | 1.11 | 490/1671/1423 | 1249/4850/4491 | 1.06 | 458/1654/1306 | 1253/4253/3843 | 1.03 | 41/173/183 | 42/152/182 |
| exm1002264 | rs6580742 | 12 | 50727811 | *FAM186A* | A | G | 9 | 0.20 | 0.19 | 1.11 | 1.2E-07 | 0 | 1.13 | 147/1153/2284 | 357/3151/7082 | 1.09 | 163/1150/2105 | 383/2996/5971 | 0.89 | 7/106/284 | 15/96/265 |
| exm716877 | rs16888728 | 8 | 117783975 | *UTP23* | A | G | 8 | 0.11 | 0.10 | 1.15 | 1.4E-07 | 0 | 1.16 | 41/705/2838 | 101/1831/8658 | 1.13 | 43/665/2710 | 88/1664/7597 | 0.84 | 4/79/314 | 4/88/284 |
| exm1037423 | rs3184504 | 12 | 111884608 | *SH2B3* | G | A | 9 | 0.53 | 0.51 | 1.08 | 3.9E-07 | 0 | 1.07 | 1023/1780/781 | 2816/5297/2476 | 1.09 | 932/1689/797 | 2328/4646/2374 | 1.10 | 137/178/82 | 116/177/83 |
| exm-rs2282978 | rs2282978 | 7 | 92264410 | *CDK6* | G | A | 9 | 0.34 | 0.32 | 1.08 | 1.1E-06 | 15.3 | 1.04 | 415/1569/1600 | 1124/4669/4795 | 1.12 | 366/1540/1512 | 894/3986/4469 | 0.96 | 52/170/175 | 44/179/153 |
| exm-rs653178 | rs653178 | 12 | 112007756 | *ATXN2* | A | G | 8 | 0.53 | 0.51 | 1.09 | 1.7E-06 | 0 | 1.07 | 1016/1786/782 | 2803/5307/2480 | 1.09 | 925/1692/801 | 2312/4650/2387 | 1.11 | 136/180/81 | 115/177/84 |
| exm-rs1209950 | rs1209950 | 21 | 40173528 | | A | G | 6 | 0.43 | 0.41 | 1.09 | 7.3E-06 | 0 | 1.07 | 661/1711/1212 | 1732/5175/3682 | 1.10 | 629/1671/1118 | 1554/4472/3322 | 1.30 | 83/179/135 | 49/179/147 |
| exm-rs10774625 | rs10774625 | 12 | 111910219 | *ATXN2* | G | A | 6 | 0.52 | 0.49 | 1.09 | 1.1E-05 | 0 | 1.07 | 967/1786/831 | 2664/5300/2626 | 1.09 | 869/1697/852 | 2184/4642/2524 | 1.15 | 135/180/82 | 111/176/89 |
| exm2265440 | rs6599132 | 3 | 41039907 | | G | A | 9 | 0.57 | 0.55 | 1.08 | 2.5E-05 | 0 | 1.06 | 1152/1798/634 | 3256/5300/2033 | 1.11 | 1115/1670/633 | 2735/4708/1907 | 1.11 | 113/203/80 | 102/184/90 |
| exm-rs11169552 | rs11169552 | 12 | 51155663 | | G | A | 9 | 0.75 | 0.73 | 1.08 | 2.6E-05 | 0 | 1.08 | 1985/1375/224 | 5670/4117/801 | 1.10 | 1910/1309/199 | 5000/3687/663 | 1.04 | 229/145/23 | 217/132/27 |
| exm-rs7315438 | rs7315438 | 12 | 115891403 | | A | G | 9 | 0.59 | 0.57 | 1.08 | 3.0E-05 | 14.3 | 1.06 | 1280/1697/604 | 3585/5093/1911 | 1.09 | 1189/1631/597 | 2971/4635/1741 | 1.05 | 159/176/62 | 147/163/65 |
| exm235708 | rs78446341 | 2 | 160690656 | *LY75* | A | G | 6 | 0.03 | 0.02 | 1.27 | 3.3E-05 | 0 | 1.24 | 4/191/3389 | 7/461/10122 | 1.30 | 1/226/3191 | 4/479/8867 | 1.30 | 0/15/382 | 0/11/365 |
| exm1556471 | rs2236200 | 20 | 60986019 | *C20orf151* | A | C | 9 | 0.76 | 0.74 | 1.08 | 3.6E-05 | 0 | 1.09 | 2056/1320/208 | 5813/4080/696 | 1.10 | 1986/1246/185 | 5220/3502/624 | 1.13 | 232/144/20 | 208/145/23 |
| exm1002260 | rs6580741 | 12 | 50727706 | *FAM186A* | G | C | 6 | 0.37 | 0.35 | 1.08 | 3.9E-05 | 19.9 | 1.11 | 490/1675/1419 | 1252/4850/4485 | 1.07 | 459/1651/1307 | 1250/4229/3842 | 1.04 | 42/172/183 | 42/152/182 |
| exm1002762 | rs861204 | 12 | 51237816 | *TMPRSS12* | G | A | 9 | 0.67 | 0.66 | 1.07 | 4.2E-05 | 5.75 | 1.08 | 1623/1572/388 | 4547/4801/1242 | 1.06 | 1545/1485/388 | 4028/4206/1112 | 0.97 | 168/175/54 | 154/181/41 |
| exm1002276 | rs7296291 | 12 | 50744119 | *FAM186A* | G | A | 6 | 0.37 | 0.35 | 1.08 | 5.8E-05 | 18 | 1.11 | 490/1671/1423 | 1249/4850/4491 | 1.06 | 457/1654/1306 | 1250/4254/3843 | 1.03 | 42/172/183 | 42/153/181 |
| exm1488109 | rs2307019 | 19 | 49244220 | *IZUMO1* | G | A | 9 | 0.59 | 0.58 | 1.07 | 6.1E-05 | 2.3 | 1.07 | 1229/1719/635 | 3410/5168/2010 | 1.07 | 1315/1612/489 | 3378/4537/1432 | 1.33 | 126/195/76 | 90/191/95 |
| exm-rs2548145 | rs2548145 | 5 | 40134777 | | G | A | 8 | 0.54 | 0.52 | 1.07 | 6.9E-05 | 1.98 | 1.08 | 1053/1763/768 | 2859/5288/2442 | 1.10 | 998/1735/685 | 2557/4641/2151 | 0.88 | 92/194/111 | 89/204/83 |
| exm-rs11869286 | rs11869286 | 17 | 37813856 | *STARD3* | C | G | 6 | 0.34 | 0.32 | 1.08 | 7.3E-05 | 0 | 1.08 | 423/1652/1509 | 1160/4699/4730 | 1.08 | 387/1500/1530 | 929/4046/4358 | 1.13 | 50/186/161 | 44/162/170 |

| | Spain | | | Germany | | | Portugal | | | VQ | | | London GWAS | | | Scotland GWAS | | |
|---|---|---|---|---|---|---|---|---|---|---|---|---|---|---|---|---|---|---|
| **SNP** | **OR.fixed** | **Cases** | **Controls** | **OR.fixed** | **Cases** | **Controls** | **OR.fixed** | **Cases** | **Controls** | **OR.fixed** | **Cases** | **Controls** | **OR.fixed** | **Cases** | **Controls** | **OR.fixed** | **Cases** | **Controls** |
| exm-rs4939827 | 1.26 | 99/115/45 | 78/143/52 | 1.23 | 80/116/51 | 267/533/253 | 1.07 | 61/92/42 | 53/83/42 | 1.16 | 299/467/182 | 143/280/115 | 1.19 | 968/1494/571 | 1001/1847/842 | 1.15 | 171/279/106 | 829/1474/694 |
| exm-rs6983267 | 1.13 | 91/124/44 | 90/123/58 | 1.28 | 76/123/47 | 261/528/264 | 1.07 | 71/86/38 | 57/88/33 | 1.13 | 272/481/194 | 141/268/129 | 1.24 | 956/1510/567 | 975/1813/900 | 1.26 | 174/292/90 | 821/1461/715 |
| exm-rs7014346 | 1.21 | 48/117/94 | 42/114/117 | 1.20 | 40/123/84 | 144/487/422 | 1.19 | 32/96/67 | 26/79/73 | 1.24 | 144/471/333 | 67/243/228 | 1.20 | 528/1441/1064 | 518/1674/1497 | 1.18 | 84/291/181 | 429/1385/1183 |
| exm-rs10505477 | 1.15 | 87/124/48 | 85/123/64 | 1.25 | 71/125/51 | 245/532/276 | 1.10 | 66/90/39 | 53/88/37 | 0.87 | 05/04/2006 | 137/267/134 | 1.21 | 758/1244/492 | 722/1380/689 | 1.31 | 58/109/29 | 530/992/500 |
| exm-rs4779584 | 1.47 | 12/84/163 | 6/71/196 | 1.20 | 16/84/147 | 37/356/660 | 1.54 | 7/68/120 | 4/45/129 | 1.15 | 51/293/605 | 15/170/353 | 1.22 | 163/998/1871 | 126/1117/2446 | 1.27 | 18/123/219 | 37/279/659 |
| exm-rs16892766 | 1.17 | 0/35/224 | 2/28/243 | 1.33 | 6/41/200 | 1/174/878 | 1.44 | 3/39/153 | 3/23/152 | 0.94 | 1/0/14 | 3/70/465 | 1.36 | 24/555/2454 | 19/520/3151 | 1.17 | 5/98/453 | 19/467/2511 |
| exm-rs961253 | 1.14 | 32/122/105 | 28/124/121 | 1.24 | 50/95/102 | 127/467/459 | 1.16 | 26/91/78 | 22/74/82 | 0.96 | 0/11/4 | 77/251/210 | 1.10 | 460/1399/1174 | 477/1698/1515 | 1.25 | 93/266/197 | 386/1344/1267 |
| exm-rs6687758 | 1.23 | 14/88/156 | 9/86/178 | 1.20 | 9/92/146 | 38/331/684 | 1.08 | 6/66/123 | 5/57/116 | 0.97 | 48/306/592 | 24/186/326 | 1.11 | 120/1040/1872 | 129/1180/2381 | 1.21 | 30/192/334 | 110/946/1941 |
| exm-rs4925386 | 1.12 | 126/111/22 | 124/122/27 | 0.96 | 119/100/28 | 491/475/87 | 1.10 | 99/80/16 | 82/83/13 | 1.09 | 469/392/88 | 253/228/57 | 1.10 | 1505/1285/243 | 1746/1590/354 | 1.10 | 97/82/17 | 942/885/195 |
| exm1002721 | 1.37 | 50/145/64 | 42/135/96 | 0.98 | 42/107/98 | 180/466/407 | 1.04 | 39/96/60 | 38/79/61 | NA | NA | NA | NA | NA | NA | NA | NA | NA |
| exm-rs10411210 | 0.82 | 196/61/2 | 218/52/3 | 1.32 | 217/28/2 | 885/159/9 | 1.80 | 162/30/3 | 126/49/3 | 0.86 | 12/03/2000 | 447/88/3 | 1.20 | 2563/448/21 | 3014/647/29 | 1.38 | 479/73/3 | 2451/522/24 |
| exm1002434 | 1.33 | 40/138/81 | 34/127/112 | 0.94 | 25/105/117 | 128/437/488 | 0.96 | 25/102/68 | 27/88/63 | 1.17 | 102/495/352 | 53/255/230 | 1.13 | 423/1425/1184 | 421/1711/1558 | 1.00 | 22/98/76 | 268/929/825 |
| exm1002264 | 1.23 | 9/78/172 | 8/69/196 | 0.91 | 7/61/179 | 35/277/741 | 1.12 | 7/56/132 | 5/48/125 | 1.22 | 34/310/605 | 11/161/366 | 1.12 | 132/985/1916 | 128/1133/2429 | 1.09 | 25/183/348 | 112/947/1938 |
| exm716877 | 1.12 | 1/37/221 | 2/33/238 | 1.30 | 8/44/195 | 4/195/854 | 1.38 | 7/45/143 | 2/36/140 | NA | NA | NA | 1.20 | 29/479/1986 | 20/470/2302 | 1.15 | 3/36/157 | 12/359/1651 |
| exm1037423 | 1.12 | 75/140/44 | 78/134/60 | 1.18 | 64/130/53 | 250/512/291 | 1.08 | 60/91/44 | 48/90/40 | 1.19 | 270/468/211 | 123/279/136 | 1.07 | 876/1532/620 | 1012/1847/825 | 0.99 | 133/291/130 | 769/1490/737 |
| exm-rs2282978 | 1.13 | 50/125/84 | 43/135/95 | 0.95 | 29/101/116 | 107/492/454 | 1.23 | 42/90/63 | 25/91/62 | 1.15 | 116/407/425 | 54/220/264 | 1.09 | 362/1341/1329 | 372/1623/1695 | 1.19 | 72/250/234 | 317/1254/1426 |
| exm-rs653178 | 1.14 | 75/140/44 | 77/135/61 | 1.17 | 62/132/53 | 249/511/293 | 1.06 | 60/91/44 | 48/91/39 | 1.20 | 270/469/210 | 122/279/136 | 1.01 | 134/281/124 | 230/452/216 | NA | NA | NA |
| exm-rs1209950 | 1.08 | 57/126/76 | 56/130/87 | 1.06 | 39/119/89 | 154/502/397 | 1.12 | 33/101/61 | 27/89/62 | NA | NA | NA | NA | NA | NA | NA | NA | NA |
| exm-rs10774625 | 1.09 | 73/139/47 | 77/134/61 | 1.20 | 63/130/54 | 243/510/300 | 1.09 | 60/90/45 | 46/92/40 | NA | NA | NA | NA | NA | NA | NA | NA | NA |
| exm2265440 | 1.18 | 97/126/36 | 94/127/52 | 1.10 | 78/123/46 | 307/529/217 | 0.90 | 68/87/40 | 62/89/27 | 0.98 | 296/471/182 | 180/247/111 | 1.02 | 166/272/101 | 278/440/180 | 1.06 | 111/172/77 | 290/458/227 |
| exm-rs11169552 | 0.83 | 149/95/15 | 172/88/13 | 0.94 | 132/102/13 | 598/388/67 | 1.25 | 123/63/9 | 101/66/11 | 1.18 | 553/348/48 | 287/218/33 | 1.06 | 1698/1115/220 | 1974/1452/263 | 1.04 | 290/231/33 | 1574/1199/224 |
| exm-rs7315438 | 0.96 | 68/127/64 | 70/143/60 | 1.22 | 96/118/32 | 347/538/168 | 0.75 | 45/92/58 | 49/94/35 | 1.10 | 339/466/144 | 174/277/87 | 1.13 | 202/258/79 | 300/451/147 | 1.11 | 122/187/51 | 326/468/181 |
| exm235708 | 1.69 | 0/11/248 | 0/7/266 | 1.03 | 0/7/240 | 0/29/1024 | 1.10 | 0/12/183 | 0/10/168 | NA | NA | NA | NA | NA | NA | NA | NA | NA |
| exm1556471 | 1.07 | 148/93/18 | 142/118/12 | 1.11 | 141/101/5 | 603/389/61 | 1.12 | 128/57/10 | 108/63/7 | 1.05 | 566/332/50 | 322/178/38 | 1.04 | 1686/1164/182 | 2002/1465/223 | 1.00 | 109/75/12 | 1122/777/123 |
| exm1002260 | 1.35 | 39/140/80 | 33/128/112 | 0.94 | 25/105/117 | 128/436/489 | 0.96 | 25/100/70 | 27/86/65 | NA | NA | NA | NA | NA | NA | NA | NA | NA |
| exm1002762 | 1.45 | 120/117/22 | 103/123/46 | 1.14 | 110/109/28 | 431/477/145 | 0.89 | 85/83/27 | 79/82/17 | 1.01 | 402/442/104 | 222/260/56 | 1.07 | 1432/1262/334 | 1627/1649/407 | 1.07 | 256/242/57 | 1314/1348/334 |
| exm1002276 | 1.33 | 40/138/81 | 34/127/112 | 0.94 | 25/105/117 | 128/437/488 | 0.96 | 25/102/68 | 27/88/63 | NA | NA | NA | NA | NA | NA | NA | NA | NA |
| exm1488109 | 1.06 | 81/127/51 | 83/130/60 | 1.16 | 77/122/48 | 284/532/237 | 1.04 | 56/93/46 | 55/73/50 | 0.94 | 312/460/177 | 194/244/100 | 1.10 | 189/259/91 | 286/450/162 | 1.04 | 136/176/48 | 372/450/153 |
| exm-rs2548145 | 1.04 | 80/127/52 | 87/123/63 | 1.06 | 62/133/52 | 272/520/261 | 1.08 | 66/96/33 | 54/94/30 | 1.02 | 273/456/220 | 144/274/120 | 0.97 | 146/278/115 | 251/463/184 | NA | NA | NA |
| exm-rs11869286 | 1.22 | 37/134/88 | 34/127/112 | 1.05 | 25/108/114 | 111/430/512 | 1.02 | 29/94/72 | 25/87/66 | NA | NA | NA | NA | NA | NA | NA | NA | NA |

**Supplementary Table 5. Results of conditional analysis for 12q24.12 locus.**

| SNP | A1 | RsID | CHR | BP | PPgene | Annotation | EAF | Results of meta-analysis | | | Conditional to rs3184504 | | |
|---|---|---|---|---|---|---|---|---|---|---|---|---|---|
| | | | | | | | | N | OR.fixed | P.fixed | N.cond | OR.cond | P.cond |
| exm1037167 | C | rs200420920 | 12 | 111652019 | CUX2 | missense | 0.9995 | 2 | 1.1103 | 0.8273 | 2 | 1.1422 | 0.7817 |
| exm1037169 | A | rs199531850 | 12 | 111652040 | CUX2 | missense | 0.0003 | 2 | 1.7067 | 0.3008 | 2 | 1.7488 | 0.2794 |
| exm1037224 | G | rs201856438 | 12 | 111744903 | CUX2 | missense | 0.0002 | 2 | 1.5745 | 0.5784 | 2 | 1.492 | 0.6242 |
| exm1037295 | G | rs201719553 | 12 | 111776225 | CUX2 | missense | 0.0002 | 2 | 1.1957 | 0.8288 | 2 | 1.2584 | 0.7812 |
| exm1037299 | A | rs200121526 | 12 | 111779619 | CUX2 | missense | 0.0002 | 2 | 1.6207 | 0.4773 | 2 | 1.6255 | 0.4748 |
| exm1037318 | A | rs61745424 | 12 | 111785515 | CUX2 | missense | 0.0240 | 6 | 1.1162 | 0.06097 | 6 | 1.0841 | 0.1722 |
| exm1037367 | A | rs201849141 | 12 | 111800849 | FAM109A | missense | 0.0023 | 4 | 1.2243 | 0.2721 | 4 | 1.1781 | 0.3745 |
| exm1037423 | G | rs3184504 | 12 | 111884608 | SH2B3 | missense | 0.5072 | 9 | 1.0822 | 3.877E-07 | #N/A | #N/A | #N/A |
| exm1037447 | G | rs72650673 | 12 | 111885310 | SH2B3 | missense | 0.9970 | 2 | 1.1421 | 0.471 | 2 | 1.1878 | 0.3511 |
| exm1037482 | A | rs72650662 | 12 | 111886074 | SH2B3 | missense | 0.0003 | 2 | 0.9477 | 0.926 | 2 | 0.9125 | 0.8741 |
| exm1037483 | A | rs148791142 | 12 | 111886075 | SH2B3 | missense | 0.0003 | 2 | 1.0846 | 0.8781 | 2 | 1.0283 | 0.9579 |
| exm1037484 | G | rs199803113 | 12 | 111886081 | SH2B3 | missense | 0.0003 | 2 | 2.4032 | 0.03785 | 2 | 2.3719 | 0.04087 |
| exm1037527 | G | rs140262591 | 12 | 111908545 | ATXN2 | coding-synon | 0.0057 | 5 | 1.0563 | 0.6539 | 5 | 1.0189 | 0.8786 |
| exm-rs10774625 | G | rs10774625 | 12 | 111910219 | ATXN2 | intron | 0.4930 | 6 | 1.0851 | 1.06E-05 | 6 | 0.9971 | 0.9698 |
| exm1037532 | A | rs142462470 | 12 | 111923594 | ATXN2 | missense | 0.0004 | 4 | 1.1942 | 0.6806 | 4 | 1.1424 | 0.7576 |
| exm1037574 | G | rs117851901 | 12 | 111956226 | ATXN2 | missense | 0.0034 | 2 | 1.0263 | 0.8719 | 2 | 1.0686 | 0.6812 |
| exm1037605 | G | rs7969300 | 12 | 111993712 | ATXN2 | missense | 0.9977 | 7 | 1.3863 | 0.1142 | 6 | 1.4486 | 0.07365 |
| exm-rs653178 | A | rs653178 | 12 | 112007756 | ATXN2 | intron | 0.5058 | 8 | 1.0878 | 1.71E-06 | 8 | 0.9702 | 0.8762 |
| exm-rs11065987 | A | rs11065987 | 12 | 112072424 | | | 0.5747 | 9 | 1.0631 | 6.30E-04 | 9 | 0.9816 | 0.5983 |
| exm1037707 | A | rs148204415 | 12 | 112130611 | ACAD10 | missense | 0.0012 | 2 | 1.7007 | 0.02406 | 2 | 1.6312 | 0.03792 |
| exm1037760 | G | rs200607092 | 12 | 112165819 | ACAD10 | missense | 0.0011 | 2 | 1.2811 | 0.3422 | 2 | 1.2225 | 0.4416 |
| exm1037802 | A | rs138790472 | 12 | 112182585 | ACAD10 | missense | 0.9993 | 3 | 1.2202 | 0.6413 | 3 | 1.2863 | 0.556 |
| exm1037831 | G | rs150349412 | 12 | 112184086 | ACAD10 | missense | 0.9983 | 4 | 1.1962 | 0.4717 | 4 | 1.2559 | 0.3608 |
| exm1037842 | T | rs141918583 | 12 | 112185166 | ACAD10 | missense | 0.0004 | 2 | 1.5619 | 0.2391 | 2 | 1.6538 | 0.1845 |
| exm1037851 | G | rs34245489 | 12 | 112186274 | ACAD10 | missense | 0.9526 | 6 | 1.002 | 0.9641 | 6 | 0.973 | 0.5397 |
| exm2259959 | G | rs2238151 | 12 | 112211833 | ALDH2 | intron | 0.3202 | 6 | 1.0177 | 0.3764 | 6 | 0.9688 | 0.1578 |
| exm1037914 | G | rs147086207 | 12 | 112221070 | ALDH2 | missense | 0.0010 | 2 | 1.0448 | 0.8851 | 2 | 0.9991 | 0.9977 |

Variants used for conditional analysis are shaded grey. Previously described GWAS variant(s) are higlighted using bold font.

**Supplementary Table 6. Results of conditional analysis for 8q23.3-8q24.11 locus.**

| | | | | | | | | Results of meta-analysis | | | Conditional to rs16888728 | | | Conditional to rs16892766 | | |
|---|---|---|---|---|---|---|---|---|---|---|---|---|---|---|---|---|
| SNP | A1 | RsID | CHR | BP | PPgene | Annotation | EAF | N | OR.fixed | P.fixed | N | OR.fixed | P.fixed | N | OR.fixed | P.fixed |
| exm-rs799889 | C | rs799889 | 8 | 117250895 | | | 0.18 | 6 | 1.02 | 0.40 | 6 | 1.02 | 0.42 | 6 | 1.01 | 0.55 |
| exm-rs4876662 | A | rs4876662 | 8 | 117556270 | | | 0.19 | 6 | 1.00 | 0.92 | 6 | 1.00 | 0.96 | 6 | 0.99 | 0.67 |
| **exm-rs16892766** | **C** | **rs16892766** | **8** | **117630683** | | | 0.08 | **9** | **1.26** | **3.57E-17** | **8** | **1.27** | **5.13E-10** | **#N/A** | **#N/A** | **#N/A** |
| exm716811 | A | rs200534489 | 8 | 117658748 | *EIF3H* | missense | 0.0002 | 2 | 2.23 | 0.26 | 2 | 2.29 | 0.24 | 2 | 2.32 | 0.23 |
| exm716877 | A | rs16888728 | 8 | 117783975 | *UTP23* | missense | 0.10 | 8 | 1.15 | 1.43E-07 | #N/A | #N/A | #N/A | 8 | 0.99 | 0.83 |
| exm716893 | G | rs139935751 | 8 | 117859924 | *RAD21* | missense | 1.00 | 2 | 1.43 | 0.64 | 4 | 1.44 | 0.64 | 3 | 1.40 | 0.66 |
| exm716897 | A | rs143363239 | 8 | 117861258 | *RAD21* | missense | 0.00025 | 2 | 2.07 | 0.12 | 2 | 2.12 | 0.11 | 2 | 2.11 | 0.11 |
| exm716913 | C | rs144953114 | 8 | 117864305 | *RAD21* | missense | 0.0005 | 2 | 1.17 | 0.72 | 2 | 1.18 | 0.70 | 2 | 1.19 | 0.68 |
| exm716958 | G | rs16889042 | 8 | 117879001 | *RAD21* | intron | 1.00 | 4 | 1.04 | 0.83 | 5 | 1.15 | 0.48 | 4 | 1.04 | 0.86 |

Variants used for conditional analysis are shaded grey. Previously described GWAS variant(s) are higlighted using bold font.

**Supplementary Table 7. Results of conditional analysis for 12q13.12 locus.**

| | | | | | | | | Results of meta-analysis | | | Conditional to rs1129406 | | | Conditional to rs12303082 | | | Conditional to rs6580742 | | |
|---|---|---|---|---|---|---|---|---|---|---|---|---|---|---|---|---|---|---|---|
| **SNP** | **A1** | **RsID** | **CHR** | **BP** | **PPgene** | **Annotation** | **EAF** | **N** | **OR.fixed** | **P.fixed** | **N.cond** | **OR.cond** | **P.cond** | **N.cond** | **OR.cond** | **P.cond** | **N.cond** | **OR.cond** | **P.cond** |
| exm1002126 | A | rs146787766 | 12 | 50535840 | LASS5 | missense | 0.00018 | 2 | 2.39 | 0.15 | 2 | 2.49 | 0.13 | 2 | 2.45 | 0.14 | 2 | 2.44 | 0.14 |
| exm1002141 | G | rs7302981 | 12 | 50537815 | LASS5 | missense | 0.626 | 9 | 1.05 | 1.40E-03 | 6 | 0.99 | 0.82 | 9 | 1.01 | 0.62 | 9 | 1.02 | 0.16 |
| exm1002146 | A | rs143484198 | 12 | 50561023 | LASS5 | missense | 0.007 | 6 | 1.09 | 0.43 | 6 | 1.13 | 0.25 | 6 | 1.12 | 0.29 | 6 | 1.11 | 0.34 |
| exm1002199 | A | rs142007630 | 12 | 50586275 | LIMA1 | missense | 0.00023 | 2 | 1.45 | 0.50 | 2 | 1.55 | 0.43 | 2 | 1.52 | 0.45 | 2 | 1.50 | 0.46 |
| exm1002256 | C | rs12809349 | 12 | 50724444 | FAM186A | missense | 0.036 | 6 | 1.16 | 1.75E-03 | 6 | 1.09 | 0.07 | 6 | 1.11 | 0.04 | 6 | 1.08 | 0.15 |
| exm1002260 | G | rs6580741 | 12 | 50727706 | FAM186A | missense | 0.352 | 6 | 1.08 | 3.92E-05 | 6 | 0.96 | 0.26 | 5 | 1.41 | 0.27 | 6 | 1.05 | 0.06 |
| exm1002264 | A | rs6580742 | 12 | 50727811 | FAM186A | missense | 0.189 | 9 | 1.11 | 1.20E-07 | 6 | 1.03 | 0.26 | 9 | 1.06 | 0.04 | #N/A | #N/A | #N/A |
| exm1002266 | G | rs80201036 | 12 | 50727870 | FAM186A | nonsense | 0.990 | 6 | 1.28 | 0.01 | 6 | 1.22 | 0.04 | 6 | 1.24 | 0.03 | 6 | 1.25 | 0.02 |
| exm1002276 | G | rs7296291 | 12 | 50744119 | FAM186A | missense | 0.353 | 6 | 1.08 | 5.76E-05 | 6 | 0.96 | 0.21 | | | | 6 | 1.05 | 0.08 |
| exm1002287 | C | rs183549613 | 12 | 50744680 | FAM186A | missense | 0.0003 | 2 | 2.17 | 0.13 | 2 | 2.18 | 0.13 | 2 | 1.99 | 0.18 | 2 | 2.21 | 0.12 |
| exm1002397 | G | rs201058635 | 12 | 50748127 | FAM186A | missense | 0.998 | 3 | 1.07 | 0.74 | 3 | 1.03 | 0.90 | 3 | 1.04 | 0.86 | 3 | 1.05 | 0.83 |
| exm1002414 | C | rs4435082 | 12 | 50749221 | FAM186A | missense | 0.0002 | 3 | 1.09 | 0.89 | 3 | 1.06 | 0.93 | 3 | 0.99 | 0.99 | 3 | 1.12 | 0.86 |
| exm1002415 | C | rs4625558 | 12 | 50749227 | FAM186A | missense | 1.000 | 2 | 1.22 | 0.82 | 2 | 1.27 | 0.78 | 2 | 1.25 | 0.80 | 2 | 1.17 | 0.86 |
| exm1002419 | C | rs74090114 | 12 | 50749554 | FAM186A | missense | 0.989 | 6 | 1.03 | 0.78 | 6 | 0.99 | 0.89 | 6 | 1.00 | 0.98 | 6 | 1.01 | 0.91 |
| exm1002434 | A | rs12303082 | 12 | 50754563 | FAM186A | missense | 0.353 | 9 | 1.09 | 7.36E-08 | 6 | 0.96 | 0.21 | #N/A | #N/A | #N/A | 9 | 1.06 | 0.01 |
| exm1002436 | C | rs201711271 | 12 | 50754577 | FAM186A | missense | 0.00023 | 2 | 1.15 | 0.84 | 2 | 1.25 | 0.75 | 2 | 1.21 | 0.79 | 2 | 1.20 | 0.80 |
| exm1002440 | C | rs184587740 | 12 | 50757020 | FAM186A | missense | 0.999 | 2 | 1.33 | 0.54 | 4 | 1.24 | 0.64 | 2 | 1.27 | 0.60 | 2 | 1.28 | 0.59 |
| exm2271842 | G | rs10735825 | 12 | 50768339 | FAM186A | intron | 0.055 | 6 | 1.06 | 0.15 | 6 | 1.02 | 0.58 | 6 | 1.02 | 0.58 | 6 | 1.08 | 0.05 |
| exm1002449 | C | rs146142861 | 12 | 50821551 | LARP4 | missense | 0.976 | 6 | 1.12 | 0.06 | 6 | 1.10 | 0.14 | 6 | 1.09 | 0.17 | 6 | 1.10 | 0.13 |
| exm1002524 | A | rs201453176 | 12 | 50869569 | LARP4 | missense | 0.00013 | 2 | 1.52 | 0.50 | 2 | 1.43 | 0.57 | 2 | 1.46 | 0.55 | 2 | 1.41 | 0.59 |
| exm-rs10876041 | G | rs10876041 | 12 | 50901882 | DIP2B | intron | 0.637 | 6 | 1.05 | 0.02 | 6 | 0.96 | 0.10 | 6 | 1.00 | 0.84 | 6 | 1.02 | 0.39 |
| exm1002555 | A | rs73093419 | 12 | 51068409 | DIP2B | missense | 0.014 | 5 | 1.02 | 0.80 | 5 | 1.08 | 0.38 | 5 | 1.05 | 0.53 | 5 | 1.04 | 0.60 |
| exm1002585 | A | rs74751916 | 12 | 51080364 | DIP2B | missense | 0.021 | 6 | 1.05 | 0.41 | 6 | 0.99 | 0.93 | 6 | 1.01 | 0.91 | 6 | 1.08 | 0.24 |
| exm1002587 | C | rs148830732 | 12 | 51080389 | DIP2B | missense | 0.999 | 2 | 0.97 | 0.92 | 3 | 0.93 | 0.83 | 2 | 0.95 | 0.87 | 2 | 0.95 | 0.88 |
| exm1002627 | A | rs151181050 | 12 | 51108283 | DIP2B | missense | 0.002 | 5 | 1.24 | 0.23 | 5 | 1.31 | 0.14 | 5 | 1.28 | 0.17 | 5 | 1.27 | 0.19 |
| **exm-rs1116955** | **G** | **rs11169552** | **12** | **51155663** | | | 0.734 | **9** | **1.08** | **2.55E-05** | **6** | **1.02** | **0.35** | **9** | **1.04** | **0.03** | **9** | **1.06** | **3.60E-03** |
| exm1002721 | A | rs1129406 | 12 | 51203371 | ATF1 | coding-synon/splice | 0.403 | 6 | 1.11 | 8.27E-09 | #N/A | #N/A | #N/A | 6 | 1.15 | 1.23E-05 | 6 | 1.10 | 2.86E-05 |
| exm1002733 | G | rs2230674 | 12 | 51208122 | ATF1 | missense | 0.965 | 8 | 1.05 | 0.32 | 6 | 1.03 | 0.53 | 8 | 1.03 | 0.50 | 8 | 1.04 | 0.45 |
| exm-rs1729165( | A | rs17291650 | 12 | 51213433 | ATF1 | coding-synon | 0.905 | 9 | 1.01 | 0.72 | 6 | 0.98 | 0.50 | 8 | 0.98 | 0.62 | 9 | 0.99 | 0.73 |

Variants used for conditional analysis are shaded grey. Previously described GWAS variant(s) are higlighted using bold font.

**Supplementary Table 8. Results of conditional analysis for 1q41 locus.**

| | | | | | | | | Results of meta-analysis | | | Conditional to rs6687758 | | |
|---|---|---|---|---|---|---|---|---|---|---|---|---|---|
| **SNP** | **A1** | **RsID** | **CHR** | **BP** | **PPgene** | **Annotation** | **EAF** | **N** | **OR.fixed** | **P.fixed** | **N.cond** | **OR.cond** | **P.cond** |
| exm150731 | G | rs115082227 | 1 | 221879569 | DUSP10 | missense | 0.9957 | 4 | 1.11 | 0.50 | 3 | 1.09 | 0.54 |
| exm150738 | C | rs140139532 | 1 | 221879742 | DUSP10 | missense | 0.9998 | 3 | 1.22 | 0.73 | 3 | 1.32 | 0.64 |
| exm150778 | C | rs148146409 | 1 | 221912959 | DUSP10 | missense | 0.9996 | 4 | 1.15 | 0.78 | 4 | 1.29 | 0.61 |
| **exm-rs6687758** | **G** | **rs6687758** | **1** | **222164948** | | | 0.1955 | **9** | **1.14** | **3.15E-11** | **#N/A** | **#N/A** | **#N/A** |
| exm-rs873549 | A | rs873549 | 1 | 222271767 | | | 0.7137 | 9 | 1.00 | 0.98 | 9 | 0.99 | 0.60 |
| exm-rs17163128 | G | rs17163128 | 1 | 222619902 | | | 0.1964 | 6 | 1.04 | 0.13 | 6 | 1.02 | 0.29 |
| exm2263851 | A | rs11485177 | 1 | 222640209 | | | 0.5368 | 6 | 1.03 | 0.09 | 6 | 1.03 | 0.10 |

Variants used for conditional analysis are shaded grey. Previously described GWAS variant(s) are higlighted using bold font.

**Supplementary Table 9. Results of conditional analysis for 8q24.21 locus.**

| | | | | | | | | Results of meta-analysis | | | Conditional to rs16888728 | | | Conditional to rs7014346 | | | Conditional to rs10505477 | | | Conditional to rs10505477 and rs7014346 | | |
|---|---|---|---|---|---|---|---|---|---|---|---|---|---|---|---|---|---|---|---|---|---|---|
| **SNP** | **A1** | **RsID** | **CHR** | **BP** | **PPgene** | **Annotation** | **EAF** | **N** | **OR.fixed** | **P.fixed** | **N** | **P.fixed** | **OR.fixed** | **N.cond** | **OR.cond** | **P.cond** | **N.cond** | **OR.cond** | **P.cond** | **N.cond** | **OR.cond** | **P.cond** |
| exm-rs16902094 | G | rs16902094 | 8 | 128320346 | | | 0.141 | 6 | 1.01 | 0.78 | 6 | 1.01 | 0.85 | 6 | 0.99 | 0.73 | 6 | 1.01 | 0.76 | 6 | 0.9981 | 0.9449 |
| exm-rs445114 | A | rs445114 | 8 | 128323181 | | | 0.633 | 8 | 1.04 | 0.02 | 8 | 1.02 | 0.19 | 8 | 1.03 | 0.14 | 6 | 1.02 | 0.29 | 6 | 1.0196 | 0.3201 |
| exm-rs1562430 | A | rs1562430 | 8 | 128387852 | | | 0.571 | 9 | 1.01 | 0.47 | 9 | 1.01 | 0.71 | 9 | 1.00 | 0.93 | 9 | 1.01 | 0.48 | 9 | 1.0096 | 0.5722 |
| **exm-rs10505477** | **A** | **rs10505477** | **8** | **128407443** | ***CASC8*** | | 0.511 | **9** | **1.17** | **2.13E-21** | **9** | **0.98** | **0.73** | **9** | **1.12** | **1.57E-05** | **#N/A** | **#N/A** | **#N/A** | **#N/A** | **#N/A** | **#N/A** |
| **exm-rs6983267** | **C** | **rs6983267** | **8** | **128413305** | ***CASC8*** | | 0.520 | **9** | **1.19** | **1.09E-27** | **#N/A** | **#N/A** | **#N/A** | **9** | **1.13** | **2.96E-07** | **9** | **1.21** | **6.07E-03** | **9** | **1.19** | **0.01117** |
| **exm-rs7014346** | **A** | **rs7014346** | **8** | **128424792** | ***CASC8*** | | 0.376 | **9** | **1.17** | **4.20E-24** | **9** | **1.07** | **3.06E-03** | **#N/A** | **#N/A** | **#N/A** | **9** | **1.07** | **8.55E-03** | **#N/A** | **#N/A** | **#N/A** |
| exm-rs1447295 | A | rs1447295 | 8 | 128485038 | | | 0.100 | 9 | 1.05 | 0.12 | 9 | 1.04 | 0.15 | 9 | 1.03 | 0.36 | 7 | 1.04 | 0.19 | 7 | 1.0324 | 0.2976 |
| exm2270923 | C | rs7836840 | 8 | 128491792 | | | 0.518 | 6 | 1.01 | 0.54 | 6 | 1.03 | 0.14 | 6 | 1.02 | 0.37 | 6 | 1.03 | 0.15 | 6 | 1.0243 | 0.2037 |
| exm-rs4242382 | A | rs4242382 | 8 | 128517573 | | | 0.101 | 9 | 1.06 | 0.06 | 9 | 1.05 | 0.07 | 9 | 1.04 | 0.20 | 7 | 1.05 | 0.09 | 7 | 1.0452 | 0.1477 |
| exm-rs4242384 | C | rs4242384 | 8 | 128518554 | | | 0.100 | 8 | 1.05 | 0.12 | 8 | 1.04 | 0.16 | 8 | 1.03 | 0.35 | 7 | 1.06 | 0.08 | 7 | 1.0474 | 0.1304 |
| exm720579 | G | rs146505192 | 8 | 128750527 | *MYC* | missense | 0.001 | 4 | 1.16 | 0.62 | 4 | 1.18 | 0.56 | 4 | 1.17 | 0.59 | 4 | 1.18 | 0.57 | 4 | 1.1782 | 0.5718 |
| exm720581 | G | rs4645959 | 8 | 128750540 | *MYC* | missense | 0.040 | 9 | 1.01 | 0.88 | 9 | 1.00 | 0.91 | 9 | 1.00 | 0.97 | 9 | 1.03 | 0.47 | 9 | 1.0312 | 0.4686 |
| exm720620 | G | rs200431478 | 8 | 128752924 | *MYC* | missense | 0.9997 | 3 | 1.42 | 0.68 | 2 | 1.46 | 0.65 | 2 | 1.46 | 0.65 | 2 | 1.47 | 0.65 | 2 | 1.47 | 0.6472 |
| exm2266765 | A | rs959409 | 8 | 128920127 | | | 0.998 | 6 | 1.01 | 0.98 | 6 | 1.02 | 0.94 | 6 | 1.01 | 0.98 | 6 | 1.02 | 0.93 | 6 | 1.0126 | 0.9574 |

Variants used for conditional analysis are shaded grey. Previously described GWAS variant(s) are higlighted using bold font.

**Supplementary Table 10. Results of conditional analysis for 15q13.3 locus.**

| | | | | | | | | Results of meta-analysis | | | Conditional to rs4779584 | | |
|---|---|---|---|---|---|---|---|---|---|---|---|---|---|
| **SNP** | **A1** | **RsID** | **CHR** | **BP** | **PPgene** | **Annotation** | **EAF** | **N** | **OR.fixed** | **P.fixed** | **N.cond** | **OR.cond** | **P.cond** |
| exm1145149 | G | rs61733064 | 15 | 32925302 | *ARHGAP11A* | missense | 0.986 | 6 | 1.03 | 0.69 | 6 | 1.01 | 0.87 |
| exm1145205 | A | rs34173159 | 15 | 32929624 | *ARHGAP11A* | missense | 0.965 | 6 | 1.04 | 0.40 | 6 | 1.03 | 0.59 |
| **exm-rs4779584** | **A** | **rs4779584** | **15** | **32994756** | | | 0.188 | **9** | **1.19** | **2.3E-18** | **#N/A** | **#N/A** | **#N/A** |
| exm1145262 | G | rs199894051 | 15 | 33022968 | *GREM1* | missense | 0.00018 | 2 | 0.81 | 0.75 | 2 | 0.83 | 0.78 |
| exm1145283 | A | rs200979045 | 15 | 33091015 | *FMN1* | missense | 0.999 | 2 | 1.74 | 0.13 | 2 | 1.93 | 0.07 |
| exm2272223 | A | rs16959110 | 15 | 33106236 | *FMN1* | intron | 0.264 | 9 | 1.04 | 0.02 | 9 | 0.98 | 0.30 |
| exm1145344 | A | rs150962800 | 15 | 33260973 | *FMN1* | missense | 0.024 | 6 | 1.04 | 0.51 | 6 | 1.04 | 0.55 |
| exm1145368 | G | rs201216330 | 15 | 33261263 | *FMN1* | missense | 0.999 | 2 | 1.43 | 0.27 | 2 | 1.47 | 0.23 |

Variants used for conditional analysis are shaded grey. Previously described GWAS variant(s) are higlighted using bold font.

**Supplementary Table 11. Results of conditional analysis for 18q21.1 locus.**

| | | | | | | | | Results of meta-analysis | | | Conditional to rs4939827 | | |
|---|---|---|---|---|---|---|---|---|---|---|---|---|---|
| **SNP** | **A1** | **RsID** | **CHR** | **BP** | **PPgene** | **Annotation** | **EAF** | **N** | **OR.fixed** | **P.fixed** | **N.cond** | **OR.cond** | **P.cond** |
| exm2268151 | G | rs12454113 | 18 | 46044052 | | | 0.838 | 6 | 1.03 | 0.29 | 6 | 1.03 | 0.31 |
| exm1385990 | A | rs2277712 | 18 | 46163049 | *KIAA0427* | missense | 0.034 | 8 | 1.05 | 0.28 | 8 | 1.03 | 0.48 |
| exm1386018 | G | rs145237824 | 18 | 46284585 | *KIAA0427* | missense | 0.998 | 4 | 1.46 | 0.10 | 4 | 1.41 | 0.13 |
| exm1386072 | G | rs147123396 | 18 | 46383972 | *KIAA0427* | missense | 0.00038 | 3 | 1.14 | 0.77 | 3 | 1.01 | 0.98 |
| exm2273563 | A | rs142559064 | 18 | 46385959 | *KIAA0427* | utr-3 | 0.008 | 6 | 1.09 | 0.41 | 6 | 1.06 | 0.59 |
| **exm-rs4939827** | **A** | **rs4939827** | **18** | **46453463** | ***SMAD7*** | **intron** | 0.519 | **9** | **1.21** | **1.3E-33** | **#N/A** | **#N/A** | **#N/A** |
| exm1386154 | G | rs142608802 | 18 | 46623780 | *DYM* | missense | 0.00043 | 2 | 1.18 | 0.72 | 2 | 1.10 | 0.83 |
| exm1386166 | G | rs138427861 | 18 | 46645157 | *DYM* | missense | 0.998 | 4 | 1.16 | 0.49 | 4 | 1.21 | 0.38 |
| exm-rs11661691 | C | rs11661691 | 18 | 46770186 | *DYM* | intron | 0.523 | 6 | 1.02 | 0.29 | 6 | 1.02 | 0.38 |
| exm1386180 | G | rs145408029 | 18 | 46798603 | *DYM* | missense | 0.001 | 4 | 1.40 | 0.20 | 4 | 1.31 | 0.30 |
| exm-rs9967417 | C | rs9967417 | 18 | 46959500 | *DYM* | intron | 0.435 | 6 | 1.02 | 0.36 | 6 | 1.02 | 0.26 |
| exm2268102 | A | rs2156497 | 18 | 46976586 | *DYM* | intron | 0.664 | 6 | 1.02 | 0.38 | 6 | 1.02 | 0.41 |
| exm-rs8099594 | A | rs8099594 | 18 | 46991160 | | | 0.662 | 6 | 1.02 | 0.35 | 6 | 1.02 | 0.38 |

Variants used for conditional analysis are shaded grey. Previously described GWAS variant(s) are higlighted using bold font.

**Supplementary Table 12. Results of conditional analysis for 19q13.11 locus.**

| | | | | | | | | Results of meta-analysis | | | Conditional to rs10411210 | | |
|---|---|---|---|---|---|---|---|---|---|---|---|---|---|
| **SNP** | **A1** | **RsID** | **CHR** | **BP** | **PPgene** | **Annotation** | **EAF** | **N** | **OR.fixed** | **P.fixed** | **N.cond** | **OR.cond** | **P.cond** |
| exm1453011 | G | rs36017455 | 19 | 33465099 | *C19orf40* | missense | 0.99 | 6 | 1.02 | 0.85 | 6 | 1.02 | 0.85 |
| exm1453016 | G | rs2304103 | 19 | 33467413 | *C19orf40* | missense | 0.96 | 6 | 1.08 | 0.14 | 6 | 1.03 | 0.51 |
| exm1453018 | A | rs141801484 | 19 | 33467427 | *C19orf40* | missense | 0.0003 | 3 | 1.09 | 0.88 | 3 | 1.07 | 0.91 |
| exm1453024 | A | rs3816032 | 19 | 33467515 | *C19orf40* | missense | 0.90 | 9 | 1.03 | 0.28 | 9 | 1.01 | 0.62 |
| exm1453027 | G | rs148106526 | 19 | 33467575 | *C19orf40* | missense | 1.00 | 4 | 1.35 | 0.15 | 4 | 1.31 | 0.20 |
| **exm-rs10411210** | **G** | **rs10411210** | **19** | **33532300** | ***RHPN2*** | **intron** | 0.91 | **9** | **1.18** | **2.4E-08** | **#N/A** | **#N/A** | **#N/A** |
| exm1453177 | A | rs148710327 | 19 | 33584313 | *GPATCH1* | missense | 0.0004 | 2 | 1.62 | 0.29 | 2 | 1.59 | 0.30 |
| exm1453180 | G | rs150894192 | 19 | 33584352 | *GPATCH1* | missense | 0.9991 | 4 | 1.24 | 0.55 | 4 | 1.25 | 0.53 |
| exm1453218 | G | rs139753668 | 19 | 33588770 | *GPATCH1* | missense | 0.0010 | 4 | 1.21 | 0.48 | 4 | 1.19 | 0.52 |
| exm1453236 | A | rs2287679 | 19 | 33600764 | *GPATCH1* | missense | 0.75 | 6 | 1.02 | 0.47 | 6 | 0.96 | 0.14 |
| exm1453272 | C | rs143082587 | 19 | 33604701 | *GPATCH1* | missense | 0.0013 | 2 | 1.22 | 0.43 | 2 | 1.21 | 0.46 |
| exm1453308 | G | rs73039449 | 19 | 33616077 | *GPATCH1* | missense | 0.99 | 6 | 1.13 | 0.20 | 6 | 1.11 | 0.28 |

Variants used for conditional analysis are shaded grey. Previously described GWAS variant(s) are higlighted using bold font.

**Supplementary Table 13. Results of conditional analysis for 20p12.3 locus.**

| | | | | | | | | Results of meta-analysis | | | Conditional to rs961253 | | |
|---|---|---|---|---|---|---|---|---|---|---|---|---|---|
| **SNP** | **A1** | **RsID** | **CHR** | **BP** | **PPgene** | **Annotation** | **EAF** | **N** | **OR.fixed** | **P.fixed** | **N.cond** | **OR.cond** | **P.cond** |
| exm1524404 | G | rs2232078 | 20 | 6064805 | *FERMT1* | missense | 0.99 | 6 | 1.11 | 0.39 | 6 | 1.10 | 0.42 |
| exm1524408 | G | rs2232074 | 20 | 6065729 | *FERMT1* | missense | 0.63 | 6 | 1.02 | 0.30 | 6 | 1.02 | 0.30 |
| exm1524416 | G | rs145202913 | 20 | 6065922 | *FERMT1* | missense | 0.9990 | 3 | 1.17 | 0.64 | 3 | 1.14 | 0.70 |
| exm2254361 | G | rs35413391 | 20 | 6069723 | *FERMT1* | coding-synon | 0.93 | 6 | 1.09 | 0.03 | 6 | 1.08 | 0.03 |
| exm1524442 | A | rs202037230 | 20 | 6078265 | *FERMT1* | missense | 0.00023 | 2 | 2.01 | 0.16 | 2 | 2.01 | 0.16 |
| exm1524451 | G | rs55666319 | 20 | 6090969 | *FERMT1* | missense | 0.05 | 6 | 1.04 | 0.31 | 6 | 1.05 | 0.25 |
| exm1524465 | A | rs16991866 | 20 | 6093177 | *FERMT1* | missense | 0.90 | 8 | 1.04 | 0.20 | 8 | 1.04 | 0.15 |
| **exm-rs961253** | **A** | **rs961253** | **20** | **6404281** | | | 0.36 | **9** | **1.12** | **6.8E-12** | **#N/A** | **#N/A** | **#N/A** |
| exm1524497 | A | rs2273073 | 20 | 6750882 | *BMP2* | missense | 0.98 | 6 | 1.07 | 0.38 | 6 | 1.08 | 0.28 |

Variants used for conditional analysis are shaded grey. Previously described GWAS variant(s) are higlighted using bold font.

**Supplementary Table 14. Results of conditional analysis for 20q13.33 locus.**

| | | | | | | | | Results of meta-analysis | | | Conditional to rs4925386 | | |
|---|---|---|---|---|---|---|---|---|---|---|---|---|---|
| **SNP** | **A1** | **RsID** | **CHR** | **BP** | **PPgene** | **Annotation** | **EAF** | **N** | **OR.fixed** | **P.fixed** | **N.cond** | **OR.cond** | **P.cond** |
| exm1555380 | A | rs140197067 | 20 | 60884852 | *LAMA5* | missense | 0.0113 | 6 | 1.19 | 0.04 | 6 | 1.15 | 0.10 |
| exm1555390 | A | rs41310831 | 20 | 60885119 | *LAMA5* | missense | 0.0022 | 5 | 1.28 | 0.17 | 5 | 1.24 | 0.23 |
| exm1555393 | G | rs139502000 | 20 | 60885242 | *LAMA5* | missense | 0.9944 | 6 | 0.99 | 0.94 | 6 | 1.01 | 0.97 |
| exm1555398 | G | rs146516865 | 20 | 60885275 | *LAMA5* | missense | 0.9994 | 2 | 1.30 | 0.58 | 2 | 1.35 | 0.53 |
| exm1555403 | A | rs41307203 | 20 | 60885362 | *LAMA5* | missense | 0.0237 | 6 | 1.02 | 0.71 | 6 | 0.99 | 0.87 |
| exm2234682 | A | rs200093098 | 20 | 60885845 | *LAMA5* | missense | 0.0002 | 2 | 2.23 | 0.39 | 2 | 2.16 | 0.41 |
| exm1555432 | A | rs147595855 | 20 | 60886106 | *LAMA5* | missense | 0.9996 | 2 | 1.40 | 0.55 | 2 | 1.41 | 0.54 |
| exm1971015 | A | rs112963711 | 20 | 60886272 | *LAMA5* | missense | 0.0008 | 4 | 1.00 | 1.00 | 4 | 1.08 | 0.82 |
| exm1555461 | A | rs142756912 | 20 | 60886683 | *LAMA5* | missense | 0.0007 | 3 | 1.58 | 0.13 | 3 | 1.53 | 0.16 |
| exm1971028 | A | rs201837442 | 20 | 60887030 | *LAMA5* | missense | 0.0001 | 2 | 1.42 | 0.68 | 2 | 1.36 | 0.72 |
| exm1555486 | A | rs147777385 | 20 | 60887230 | *LAMA5* | missense | 0.0004 | 2 | 2.17 | 0.07 | 2 | 2.27 | 0.05 |
| exm1555488 | A | rs140181393 | 20 | 60887239 | *LAMA5* | missense | 0.0235 | 6 | 1.03 | 0.61 | 6 | 1.00 | 0.97 |
| exm1555503 | G | rs149357675 | 20 | 60887356 | *LAMA5* | missense | 0.9962 | 4 | 1.29 | 0.14 | 4 | 1.32 | 0.10 |
| exm1555538 | A | rs148336880 | 20 | 60888018 | *LAMA5* | missense | 0.999 | 2 | 4.32 | 0.05 | 2 | 4.40 | 0.04 |
| exm1555563 | A | rs138708242 | 20 | 60888510 | *LAMA5* | missense | 0.01 | 6 | 1.04 | 0.77 | 6 | 1.00 | 0.98 |
| exm1555588 | A | rs150774821 | 20 | 60889493 | *LAMA5* | missense | 0.9996 | 2 | 1.85 | 0.33 | 2 | 1.91 | 0.30 |
| exm1555634 | A | rs141753663 | 20 | 60890155 | *LAMA5* | missense | 0.0004 | 2 | 2.00 | 0.07 | 2 | 2.12 | 0.05 |
| exm1555643 | G | rs201926183 | 20 | 60890262 | *LAMA5* | coding-synon | 0.9996 | 2 | 1.41 | 0.54 | 2 | 1.28 | 0.66 |
| exm1555702 | A | rs140777270 | 20 | 60892813 | *LAMA5* | missense | 0.0011 | 3 | 1.37 | 0.23 | 3 | 1.32 | 0.30 |
| exm1555706 | A | rs201111971 | 20 | 60893527 | *LAMA5* | missense | 0.0001 | 2 | 4.20 | 0.12 | 2 | 3.91 | 0.14 |
| exm1555718 | G | rs150998056 | 20 | 60893611 | *LAMA5* | missense | 0.9996 | 2 | 1.53 | 0.62 | 2 | 1.57 | 0.60 |
| exm1555735 | A | rs147290767 | 20 | 60893697 | *LAMA5* | missense | 0.0029 | 5 | 1.11 | 0.51 | 5 | 1.19 | 0.29 |
| exm1555779 | A | rs140781444 | 20 | 60895806 | *LAMA5* | missense | 0.0002 | 2 | 1.45 | 0.67 | 2 | 1.45 | 0.67 |
| exm1555786 | G | rs139401504 | 20 | 60895865 | *LAMA5* | missense | 0.9988 | 4 | 1.14 | 0.67 | 4 | 1.18 | 0.59 |
| exm1555804 | A | rs141208202 | 20 | 60897104 | *LAMA5* | missense | 0.04 | 6 | 1.09 | 0.05 | 6 | 1.06 | 0.23 |
| exm1555826 | A | rs200678763 | 20 | 60897453 | *LAMA5* | missense | 0.0005 | 2 | 2.20 | 0.04 | 2 | 2.11 | 0.05 |
| exm1555881 | G | rs141989486 | 20 | 60899224 | *LAMA5* | missense | 0.9987 | 4 | 1.18 | 0.57 | 3 | 1.08 | 0.79 |
| exm1555885 | C | rs148177752 | 20 | 60899513 | *LAMA5* | missense | 0.99 | 4 | 0.99 | 0.92 | 6 | 1.03 | 0.82 |
| exm1555893 | A | rs142055388 | 20 | 60900388 | *LAMA5* | missense | 0.0032 | 4 | 1.19 | 0.25 | 4 | 1.30 | 0.09 |
| exm1555901 | A | rs2427284 | 20 | 60900481 | *LAMA5* | missense | 0.05 | 6 | 1.04 | 0.31 | 6 | 1.14 | 1.88E-03 |
| exm1555902 | A | rs149570905 | 20 | 60900490 | *LAMA5* | missense | 0.00018 | 2 | 1.87 | 0.36 | 2 | 1.81 | 0.39 |
| exm1555914 | A | rs139530736 | 20 | 60900593 | *LAMA5* | missense | 0.00008 | 2 | 1.42 | 0.68 | 2 | 1.41 | 0.69 |
| exm1555919 | A | rs11699758 | 20 | 60901762 | *LAMA5* | missense | 0.03 | 6 | 1.03 | 0.59 | 6 | 1.12 | 0.03 |
| exm1555925 | G | rs149220558 | 20 | 60901785 | *LAMA5* | missense | 0.9994 | 3 | 1.52 | 0.37 | 3 | 1.40 | 0.47 |
| exm1555929 | A | rs45496002 | 20 | 60901932 | *LAMA5* | missense | 0.01 | 6 | 1.08 | 0.41 | 6 | 1.04 | 0.64 |
| exm1555934 | A | rs875379 | 20 | 60901986 | *LAMA5* | missense | 0.09 | 9 | 1.05 | 0.08 | 9 | 1.01 | 0.59 |
| exm1555939 | A | rs150196385 | 20 | 60902022 | *LAMA5* | missense | 0.0003 | 2 | 1.76 | 0.19 | 2 | 1.70 | 0.21 |
| exm1555946 | G | rs34000043 | 20 | 60902366 | *LAMA5* | missense | 0.99 | 4 | 1.21 | 0.04 | 5 | 1.13 | 0.20 |
| exm1555957 | A | rs199963174 | 20 | 60902604 | *LAMA5* | missense | 0.00038 | 2 | 1.38 | 0.43 | 2 | 1.31 | 0.51 |
| exm1556005 | A | rs144368979 | 20 | 60904031 | *LAMA5* | missense | 0.00064 | 3 | 1.05 | 0.90 | 3 | 1.03 | 0.94 |
| exm1556030 | A | rs150741810 | 20 | 60905559 | *LAMA5* | missense | 0.00041 | 2 | 1.24 | 0.66 | 2 | 1.16 | 0.76 |
| exm1556058 | A | rs201679986 | 20 | 60906148 | *LAMA5* | missense | 0.00023 | 2 | 1.44 | 0.51 | 2 | 1.41 | 0.54 |
| exm1556077 | A | rs138521932 | 20 | 60907761 | *LAMA5* | missense | 0.01 | 4 | 1.14 | 0.29 | 4 | 1.23 | 0.10 |
| exm1556106 | G | rs13042941 | 20 | 60908969 | *LAMA5* | missense | 0.93 | 6 | 1.01 | 0.75 | 6 | 0.92 | 0.04 |
| exm1556143 | A | rs79319629 | 20 | 60910124 | *LAMA5* | missense | 0.97 | 6 | 1.06 | 0.28 | 6 | 1.10 | 0.09 |
| exm1556159 | A | rs201119098 | 20 | 60911471 | *LAMA5* | missense | 0.0004 | 2 | 1.09 | 0.86 | 2 | 1.05 | 0.92 |
| exm1556196 | A | rs199759497 | 20 | 60912983 | *LAMA5* | missense | 0.0008 | 3 | 1.72 | 0.06 | 3 | 1.66 | 0.08 |
| **exm-rs4925386** | **G** | **rs4925386** | **20** | **60921044** | ***LAMA5*** | **intron** | 0.68 | **9** | **1.11** | **8.676E-10** | **#N/A** | **#N/A** | **#N/A** |
| exm1556277 | A | rs78026347 | 20 | 60926766 | *LAMA5* | missense | 0.01 | 4 | 1.09 | 0.48 | 4 | 1.18 | 0.19 |
| exm1556279 | A | rs114928407 | 20 | 60926772 | *LAMA5* | missense | 0.0005 | 2 | 1.06 | 0.89 | 2 | 1.01 | 0.98 |
| exm1556360 | A | rs111872483 | 20 | 60963386 | *RPS21* | missense | 0.0004 | 2 | 1.44 | 0.38 | 2 | 1.53 | 0.30 |
| exm1556410 | A | rs143243918 | 20 | 60968561 | *CABLES2* | missense | 0.0006 | 2 | 1.31 | 0.45 | 2 | 1.27 | 0.50 |
| exm1556432 | G | rs41284974 | 20 | 60971397 | *CABLES2* | missense | 0.9934 | 5 | 1.28 | 0.06 | 5 | 1.19 | 0.18 |
| exm1556470 | G | rs141000397 | 20 | 60985999 | *C20orf151* | missense | 0.9998 | 2 | 1.04 | 0.95 | 2 | 1.03 | 0.97 |
| exm1556471 | A | rs2236200 | 20 | 60986019 | *C20orf151* | missense | 0.75 | 9 | 1.08 | 3.60E-05 | 9 | 1.03 | 0.10 |
| exm1556479 | G | rs138112542 | 20 | 60987715 | *C20orf151* | missense | 0.99960 | 2 | 1.27 | 0.66 | 2 | 1.33 | 0.60 |
| exm1556489 | A | rs141215868 | 20 | 60987888 | *C20orf151* | missense | 0.00028 | 3 | 1.28 | 0.67 | 3 | 1.31 | 0.65 |

ey. Previously described GWAS variant(s) are higlighted using bold font.

**Supplementary Table 15. Relationship between rs1129406 *(ATF1,* 12q13), rs12303082 *(FAM186A,* 12q13), rs6580742 *(FAM186A,* 12q13), rs16888728 *(UTP23,* 8q24) and rs3184504 *(SH2B3* , 12q24) genotypes and sex, age at diagnosis of CRC, tumour site (rectal [ICD9:154], colonic [ICD9:153]), stage and MSI status.**

| | | Age | | | Gender | | | Site | | | MSI | | | Stage (Invasive vs Non Invasive) | | |
|---|---|---|---|---|---|---|---|---|---|---|---|---|---|---|---|---|
| | | OR(95% CI) | p value | Sample Size | OR(95% CI) | p value | Sample Size | OR(95% CI) | p value | Sample Size | OR(95% CI) | p value | Sample Size | OR(95% CI) | p value | Sample Size |
| rs1129406 | exm1002721 | 0.998 (0.993-1.003) | 0.42 | 5410 | 1.059 (0.974-1.152) | 0.18 | 7964 | 0.984 (0.886-1.093) | 0.77 | 5281 | 0.998 (0.993-1.003) | 0.85 | 213 | 0.998 (0.993-1.003) | 0.40 | 4280 |
| rs12303082 | exm1002434 | 0.996 (0.991-1) | 0.06 | 5410 | 1.044 (0.96-1.135) | 0.31 | 8160 | 0.995 (0.896-1.106) | 0.93 | 5281 | 0.996 (0.991-1) | 0.76 | 213 | 0.996 (0.991-1) | 0.44 | 4280 |
| rs6580742 | exm1002264 | 0.997 (0.992-1.002) | 0.18 | 5410 | 1.053 (0.964-1.15) | 0.25 | 8461 | 0.97 (0.866-1.087) | 0.60 | 5281 | 0.997 (0.992-1.002) | 0.88 | 213 | 0.997 (0.992-1.002) | 0.53 | 4280 |
| rs16888728 | exm716877 | 0.993 (0.988-0.999) | 0.03 | 5410 | 1.209 (1.085-1.345) | 5.6E-04 | 8160 | 0.98 (0.855-1.124) | 0.77 | 5281 | 0.993 (0.988-0.999) | 0.34 | 213 | 0.993 (0.988-0.999) | 0.42 | 4280 |
| rs3184504 | exm1037423 | 1.001 (0.997-1.006) | 0.53 | 5410 | 1.005 (0.926-1.09) | 0.91 | 8459 | 0.924 (0.832-1.026) | 0.14 | 5281 | 1.001 (0.997-1.006) | 0.28 | 213 | 1.001 (0.997-1.006) | 0.13 | 4280 |

* Test is significant after correction for multiple testing (p<0.05/25)

**Supplementary Table 16. Characteristics and genotype counts of SNPs within *PRAMEF12* and *MALRD1***

| Gene | rs-number | Position | A1 | A2 | N of genotypes in cases | N of genotypes in controls | OR | P for Fisher Exact Test |
|---|---|---|---|---|---|---|---|---|
| *PCDHGA1* | rs201832666 | chr5:140790128 | A | C | 0/0/6903 | 0/3/21916 | 0 | 0.5681 |
| *PCDHGA2* | rs111794989 | chr5:140763615 | A | C | 0/58/6849 | 0/103/21821 | 1.587 | 0.005863 |
| *PCDHGA3* | rs182127695 | chr5:140795143 | G | A | 0/2/6905 | 0/5/21919 | 1.077 | 1 |
| *PCDHGA4* | rs6878145 | chr5:140718552 | G | A | 0/1/6906 | 0/2/21923 | 1.347 | 1 |
| *PCDHGB1* | rs144548345 | chr5:140718897 | G | A | 0/7/6900 | 0/15/21908 | 1.257 | 0.6331 |
| | rs17097185 | chr5:140711097 | C | G | 0/1/6906 | 0/2/21919 | 1.347 | 1 |
| | rs200981359 | chr5:140718994 | C | A | 0/1/6906 | 0/2/21923 | 1.347 | 1 |
| | rs201553091 | chr5:140719317 | A | G | 0/0/6907 | 0/2/21923 | 0 | 1 |
| | rs200811046 | chr5:140719478 | G | A | 0/1/6906 | 0/3/21922 | 0.8979 | 1 |
| | rs144241311 | chr5:140719556 | A | G | 0/22/6885 | 0/45/21880 | 1.437 | 0.1742 |
| | rs143727841 | chr5:140719633 | G | A | 0/1/6906 | 0/9/21902 | 0.2991 | 0.3051 |
| | rs199852408 | chr5:140720144 | A | C | 0/6/6901 | 0/16/21908 | 1.01 | 1 |
| | rs186274609 | chr5:140724879 | A | G | 0/11/6896 | 0/22/21903 | 1.406 | 0.3435 |
| | rs200604016 | chr5:140725033 | T | A | 0/16/6891 | 0/47/21877 | 1.291 | 0.349 |
| | rs201709248 | chr5:140726055 | C | A | 0/1/6906 | 0/2/21923 | 1.347 | 1 |
| | rs76289268 | chr5:140730210 | A | G | 0/0/6907 | 0/2/21922 | 0 | 1 |
| | rs199977912 | chr5:140730489 | A | G | 0/9/6898 | 0/37/21888 | 0.9464 | 1 |
| | rs77250251 | chr5:140731022 | G | A | 7/393/6507 | 17/1163/20744 | 1.058 | 0.314 |
| | rs200777796 | chr5:140732220 | A | C | 0/12/6895 | 0/22/21902 | 1.592 | 0.1852 |
| | rs146402451 | chr5:140734802 | G | C | 0/32/6875 | 0/82/21840 | 1.183 | 0.4073 |
| | rs201855847 | chr5:140735405 | A | C | 0/0/6902 | 0/6/21910 | 0 | 0.2001 |
| | rs201518165 | chr5:140739812 | G | A | 0/3/6904 | 0/13/21908 | 1.036 | 1 |
| | rs150944400 | chr5:140740021 | A | G | 1/0/6906 | 0/2/21922 | 4.041 | 0.1266 |
| | rs62621827 | chr5:140740060 | A | G | 0/2/6905 | 0/2/21923 | 2.695 | 0.297 |
| | rs201960802 | chr5:140742092 | A | C | 0/0/6905 | 0/4/21919 | 0 | 0.58 |
| | rs144886424 | chr5:140744055 | A | G | 0/5/6902 | 0/8/21917 | 2.021 | 0.2262 |
| | rs199512708 | chr5:140744841 | G | A | 0/7/6900 | 0/15/21908 | 1.796 | 0.1749 |
| | rs200032836 | chr5:140745129 | C | A | 0/13/6894 | 0/33/21890 | 1.304 | 0.4078 |
| | rs201155008 | chr5:140750439 | A | G | 0/1/6906 | 0/7/21914 | 0.3847 | 0.6913 |
| | rs116495533 | chr5:140750460 | C | G | 0/3/6904 | 0/3/21921 | 2.694 | 0.3539 |
| | rs199674539 | chr5:140750710 | G | A | 0/1/6906 | 0/7/21916 | 0.4489 | 0.6825 |
| | rs201701201 | chr5:140750849 | C | A | 0/3/6904 | 0/8/21917 | 1.539 | 0.5028 |
| | rs199851082 | chr5:140750868 | G | A | 0/3/6904 | 0/5/21918 | 2.155 | 0.2649 |
| | rs200031435 | chr5:140751096 | G | A | 0/7/6900 | 0/16/21907 | 1.179 | 0.8144 |
| | rs201408759 | chr5:140752321 | A | G | 0/13/6894 | 0/43/21882 | 1.476 | 0.1613 |
| | rs201390749 | chr5:140753970 | G | A | 0/11/6896 | 0/22/21903 | 1.347 | 0.4344 |
| | rs11575955 | chr5:140755901 | A | C | 0/270/6636 | 0/788/21132 | 1.119 | 0.09463 |
| | rs148240637 | chr5:140763317 | A | T | 0/3/6904 | 0/3/21908 | 2.693 | 0.354 |
| | rs141242913 | chr5:140763370 | G | A | 0/9/6898 | 0/24/21900 | 1.235 | 0.5698 |
| | rs201582947 | chr5:140763490 | A | C | 0/0/6907 | 0/12/21908 | 0.4488 | 0.3771 |
| | rs199642192 | chr5:140763515 | G | C | 0/0/6907 | 0/7/21912 | 0 | 0.201 |
| | rs185786686 | chr5:140763665 | G | A | 2/160/6745 | 0/399/21526 | 1.29 | 0.004516 |
| | rs200109598 | chr5:140768308 | C | A | 0/7/6900 | 0/28/21895 | 1.347 | 0.3854 |
| | rs144915863 | chr5:140768676 | A | G | 0/6/6901 | 0/17/21907 | 1.109 | 0.8195 |
| | rs202220616 | chr5:140768767 | G | A | 0/20/6887 | 0/52/21872 | 1.057 | 0.791 |
| | rs199638280 | chr5:140769438 | C | G | 0/5/6902 | 0/5/21920 | 2.694 | 0.1472 |
| | rs113280752 | chr5:140772736 | G | A | 0/26/6881 | 0/74/21851 | 1.123 | 0.5786 |
| | rs201697840 | chr5:140773461 | C | G | 0/20/6887 | 0/55/21867 | 1.047 | 0.8966 |
| | rs115102808 | chr5:140773738 | A | G | 1/202/6704 | 3/611/21309 | 1.064 | 0.4169 |
| | rs116789057 | chr5:140774403 | C | A | 0/2/6905 | 0/1/21923 | 1.796 | 0.6171 |
| | rs201846904 | chr5:140778163 | A | G | 0/23/6884 | 1/35/21889 | 1.676 | 0.05819 |
| | rs150385715 | chr5:140778259 | G | A | 0/26/6881 | 0/87/21838 | 1.053 | 0.8377 |
| | rs202099773 | chr5:140782608 | G | A | 0/1/6906 | 0/3/21922 | 0.898 | 1 |
| | rs199643799 | chr5:140783490 | A | C | 0/27/6880 | 0/48/21877 | 1.629 | 0.04024 |
| | rs200620626 | chr5:140784038 | G | A | 0/4/6903 | 0/10/21915 | 1.078 | 1 |
| | rs145718404 | chr5:140784495 | A | G | 0/0/6907 | 0/2/21923 | 1.347 | 1 |
| | rs200974828 | chr5:140784636 | A | G | 0/1/6906 | 1/14/21909 | 0.1584 | 0.0582 |
| | rs17097274 | chr5:140784892 | C | G | 0/2/6905 | 0/1/21922 | 1.796 | 0.6171 |
| | rs115772303 | chr5:140788731 | A | G | 0/0/6907 | 0/8/21917 | 0 | 0.201 |
| | rs186373896 | chr5:140788965 | G | A | 0/2/6905 | 0/0/21923 | NA | 0.0733 |
| | rs199531162 | chr5:140789304 | G | C | 0/20/6887 | 0/64/21860 | 0.8843 | 0.7203 |
| | rs201698858 | chr5:140789981 | A | G | 0/0/6905 | 0/4/21918 | 0 | 0.3328 |
| | rs6891442 | chr5:140790092 | C | A | 0/4/6903 | 0/3/21922 | 3.592 | 0.09126 |
| | rs11575962 | chr5:140794963 | A | G | 0/32/6749 | 0/79/21630 | 1.244 | 0.2914 |
| | rs200868391 | chr5:140795153 | A | G | 0/5/6902 | 0/14/21910 | 0.962 | 1 |
| | rs201327680 | chr5:140798669 | G | A | 0/2/6905 | 0/4/21921 | 2.021 | 0.3974 |
| | rs185228661 | chr5:140798742 | A | C | 0/0/6907 | 0/2/21923 | 0 | 1 |
| | rs200899065 | chr5:140799306 | A | G | 0/2/6905 | 0/7/21918 | 0.7696 | 1 |
| | rs200342957 | chr5:140801897 | A | G | 0/16/6891 | 0/26/21897 | 1.697 | 0.09058 |
| | rs199795822 | chr5:140802002 | G | A | 0/3/6904 | 0/6/21918 | 1.347 | 0.7105 |
| | rs199507728 | chr5:140802374 | A | G | 0/13/6894 | 0/32/21893 | 1.094 | 0.74 |
| | rs141810253 | chr5:140803055 | G | A | 0/8/6896 | 0/20/21890 | 1.616 | 0.2305 |
| | rs114008539 | chr5:140803260 | A | C | 0/2/6905 | 0/5/21918 | 0.8978 | 1 |
| | rs143083513 | chr5:140810655 | G | A | 0/3/6904 | 0/4/21919 | 2.02 | 0.3974 |
| | rs150444699 | chr5:140856212 | G | A | 0/2/6905 | 0/6/21919 | 0.8979 | 1 |
| | rs140933475 | chr5:140856972 | A | G | 0/8/6899 | 0/16/21909 | 1.617 | 0.2546 |
| | rs114678203 | chr5:140864858 | A | C | 0/4/6903 | 0/10/21915 | 1.617 | 0.3982 |
| | rs76923861 | chr5:140865264 | G | A | 5/348/6554 | 16/1017/20891 | 1.081 | 0.1815 |
| | rs144347539 | chr5:140866832 | C | G | 0/83/6824 | 2/218/21700 | 1.085 | 0.5218 |
| | rs201458212 | chr5:140867006 | T | A | 0/1/6906 | 0/8/21916 | 0.3367 | 0.4598 |
| | rs116370895 | chr5:140867061 | A | C | 0/11/6896 | 0/41/21883 | 0.9669 | 1 |
| | rs115565444 | chr5:140867087 | A | G | 0/1/6906 | 0/4/21921 | 0.6734 | 1 |
| | rs151293422 | chr5:140867123 | G | A | 0/10/6897 | 1/26/21898 | 1.058 | 0.8579 |
| | rs199722860 | chr5:140867277 | A | G | 0/8/6899 | 0/16/21904 | 1.616 | 0.2547 |
| | rs2233601 | chr5:140869229 | A | G | 0/4/6903 | 0/12/21913 | 1.122 | 0.789 |
| | rs2233603 | chr5:140869630 | A | G | 0/2/6905 | 0/2/21923 | 2.021 | 0.3974 |
| | rs141484080 | chr5:140870165 | A | G | 0/3/6904 | 0/7/21916 | 1.154 | 0.7361 |
| | rs201409669 | chr5:140870270 | A | G | 0/5/6902 | 0/12/21912 | 1.347 | 0.5965 |
| | rs141959335 | chr5:140870828 | G | A | 0/1/6906 | 0/5/21920 | 0 | 0.58 |
| | rs200418116 | chr5:140874422 | A | G | 0/2/6905 | 0/2/21923 | 1.796 | 0.6171 |
| | rs61749029 | chr5:140890616 | A | G | 0/5/6902 | 0/7/21918 | 3.08 | 0.03657 |

**Supplementary Table 17. Gene Ontology (GO) enrichment analysis.**

| GO Term | Description | P-value | FDR q-value | Enrichment (=(b/n) / (B/N)) | Total number of genes | Total number of genes associated with a specific GO term | Number of genes in the top of the user's input list | Number of genes in the intersection | Genes |
|---|---|---|---|---|---|---|---|---|---|
| GO:0007156 | homophilic cell adhesion via plasma membrane adhesion molecules | 2.36E-24 | 2.93E-20 | 20.57 | 11710 | 133 | 107 | 25 | [PCDHA8, PCDHA7, PCDHA6, PCDHA5, PCDHA4, PCDHA3, PCDHA2, PCDHA1, PCDHGB3, PCDHGB2, PCDHGB1, CELSR2, PCDHGB5, CADM3, PCDHGA7, PCDHGA6, PCDHGA3, PCDHGA2, PCDHGA5, PCDHGA4, PCDHGA1, FAT3, PCDHB1, PCDHB8, FAT1] |
| GO:0098742 | cell-cell adhesion via plasma-membrane adhesion molecules | 8.46E-22 | 5.25E-18 | 16.58 | 11710 | 165 | 107 | 25 | [PCDHA8, PCDHA7, PCDHA6, PCDHA5, PCDHA4, PCDHA3, PCDHA2, PCDHA1, PCDHGB3, PCDHGB2, PCDHGB1, CELSR2, PCDHGB5 , CADM3 , PCDHGA7, PCDHGA6 , PCDHGA3, PCDHGA2, PCDHGA5, PCDHGA4, PCDHGA1, FAT3 , PCDHB1 , PCDHB8, FAT1] |
| GO:0098609 | cell-cell adhesion | 8.03E-17 | 3.32E-13 | 9.27 | 11710 | 406 | 84 | 27 | [PCDHA8 , PCDHA7 , PCDHA6 , PCDHA5, PCDHA4, PCDHA3, PCDHA2, PCDHA1 , PCDHGB3 , PCDHGB2 , PCDHGB1 , GPR98 , CELSR2 , PCDHGB5, CLIC1, PCDHGA7, PCDHGA6 , PCDHGA3, PCDHGA2 , PCDHGA5 , PCDHGA4, PCDHGA1, FAT3, PCDHB1, IRF4, IL7R, FAT1] |
| GO:0007155 | cell adhesion | 4.09E-13 | 1.27E-09 | 5.61 | 11710 | 727 | 89 | 31 | [PCDHA8, PCDHA7, PCDHA6, PCDHA5, PCDHA4, PCDHA3, PCDHA2, PCDHA1, HEPACAM, PCDHGB3, PCDHGB2, ITGB6, PCDHGB1, GPR98, CELSR2 , PCDHGB5, CLIC1, PCDHGA7, PCDHGA6, PCDHGA3, PCDHGA2, PCDHGA5, PCDHGA4 , PCDHGA1, SLAMF7, FAT3, PCDHB1, IRF4, IL7R, COL17A1, FAT1] |
| GO:0022610 | biological adhesion | 4.26E-13 | 1.06E-09 | 5.6 | 11710 | 728 | 89 | 31 | [PCDHA8 8, PCDHA7, PCDHA6, PCDHA5, PCDHA4, PCDHA3, PCDHA2, PCDHA1, HEPACAM, PCDHGB3, ITGB6, PCDHGB2, PCDHGB1, GPR98, CELSR2, PCDHGB5, CLIC1, PCDHGA7, PCDHGA6, PCDHGA3, PCDHGA2, PCDHGA5 , PCDHGA4, PCDHGA1, SLAMF7, FAT3, PCDHB1, IRF4 IL7R , COL17A1, FAT1] |
| GO:0007399 | nervous system development | 5.75E-06 | 1.19E-02 | 7.37 | 11710 | 169 | 94 | 10 | [PCDHA8, PCDHA7, GPR98, PCDHA6, PCDHA5, EP300, PCDHA4, PCDHA3, PCDHA2, PCDHA1] |
| GO:2000400 | positive regulation of thymocyte aggregation | 1.71E-05 | 3.03E-02 | 16.6 | 11710 | 8 | 441 | 5 | [RASGRP1, GLI2, TESPA1, IL7R, VNN1] |
| GO:0033089 | positive regulation of T cell differentiation in thymus | 1.71E-05 | 2.65E-02 | 16.6 | 11710 | 8 | 441 | 5 | [RASGRP1, GLI2, TESPA1, IL7R, VNN1] |
| GO:0001539 | cilium or flagellum-dependent cell motility | 2.44E-05 | 3.37E-02 | 8.05 | 11710 | 11 | 926 | 7 | [DNAH17, DNAH3, DNAH1, DRC1, DNAH7, DNAH8, DNAH6] |
| GO:0007018 | microtubule-based movement | 8.23E-05 | 1.02E-01 | 2.92 | 11710 | 155 | 518 | 20 | [DNAH17 , KIF14, NDE1, TTC21A, STK36, KIF15, KIF21B, DNAH11, RASGRP1, IFT74, DNHD1, KIF26A, DNAH1, CELSR2, STARD9, IFT122, DNAH8, DNAH6, HEATR2, KIF27] |
| GO:0060989 | lipid tube assembly involved in organelle fusion | 8.54E-05 | 9.64E-02 | 11,710.00 | 11710 | 1 | 1 | 1 | [PCDHGA3] |
| GO:0048731 | system development | 1.08E-04 | 1.12E-01 | 3.89 | 11710 | 426 | 99 | 14 | [MAPK9, PCDHA8, PCDHA7, PCDHA6, PCDHA5, PCDHA4, PCDHA3, PCDHA2, PCDHA1, GPR98, KIF26A , EP300, CELSR2, SH3GL1] |
| GO:0021914 | negative regulation of smoothened signaling pathway involved in ventral spinal cord patterning | 2.01E-04 | 1.92E-01 | 21.25 | 11710 | 3 | 551 | 3 | [TULP3, IFT122, RFX4] |
| GO:0021952 | central nervous system projection neuron axonogenesis | 2.88E-04 | 2.55E-01 | 6.01 | 11710 | 14 | 974 | 7 | [PAFAH1B1, MYCBP2, GLI2, SZT2, CDH11, EPHB2, PLXNA4] |
| GO:2000398 | regulation of thymocyte aggregation | 2.95E-04 | 2.44E-01 | 6.61 | 11710 | 20 | 620 | 7 | [RASGRP1, GLI2, TESPA1, IL7R, BMP4, SOS2, VNN1] |
| GO:0033081 | regulation of T cell differentiation in thymus | 2.95E-04 | 2.29E-01 | 6.61 | 11710 | 20 | 620 | 7 | [RASGRP1, GLI2, TESPA1, IL7R, BMP4, SOS2, VNN1] |
| GO:0048625 | myoblast fate commitment | 3.66E-04 | 2.67E-01 | 70.54 | 11710 | 2 | 166 | 2 | [TCF7L2, EPAS1] |
| GO:0021955 | central nervous system neuron axonogenesis | 4.47E-04 | 3.08E-01 | 5.06 | 11710 | 19 | 974 | 8 | [PAFAH1B1, MYCBP2, GLI2, SZT2, CDH11, EPHB2, NDEL1, PLXNA4 ] |
| GO:0006427 | histidyl-tRNA aminoacylation | 6.90E-04 | 4.51E-01 | 52.75 | 11710 | 2 | 222 | 2 | [HARS2, HARS] |
| GO:0060988 | lipid tube assembly | 7.47E-04 | 4.64E-01 | 3,903.33 | 11710 | 3 | 1 | 1 | [PCDHGA3] |

The system has recognized 12826 genes out of 16584 gene terms entered by the user.
0 genes were recognized by gene symbol and 12826 genes by other gene IDs .
1 duplicate genes were removed (keeping the highest ranking instance of each gene) leaving a total of 12825 genes.
Only 11710 of these genes are associated with a GO term.

**Supplementary Table 18. (1) Candidate dominant high-penetrance CRC alleles; (2) Candidate recessive high-penetrance CRC alleles.**

**(1) Dominant high-penetrance alleles**

| SNP | RsID | Gene | Mutation | Position | AFF | UNAFF | P | A1 | A2 | ALL.cases | ALL.controls | ENGLAND.cases | ENGLAND.controls | Scotland.cases | Scotland.controls | PORTUGAL.cases | PORTUGAL.controls | HOLLAND.cases | HOLLAND.controls | SPAIN.cases | SPAIN.controls | GERMANY.cases | GERMANY.controls | pp_score_mean | pp_score_mean_predic | SIFT_score | SIFT_prediction | SIFT_cons | SIFT_OMIM | pp_SIFT_agree | seattle_function_first | anno_type | ExAC freq (EUR,non-Finnish)* |
|---|---|---|---|---|---|---|---|---|---|---|---|---|---|---|---|---|---|---|---|---|---|---|---|---|---|---|---|---|---|---|---|---|---|
| ***Stop mutations*** | | | | | | | | | | | | | | | | | | | | | | | | | | | | | | | | | |
| exm1440289 | rs199995129 | *NWD1* | Nonsense_R1390X | chr19:16918828 | 4/8096 | 0/21819 | 0.00537 | A | G | 0/4/8096 | 0/0/21819 | 0/3/3581 | 0/0/10590 | 0/1/3417 | 0/0/9349 | 0/0/195 | 0/0/178 | 0/0/397 | 0/0/376 | 0/0/259 | 0/0/273 | 0/0/247 | 0/0/1053 | NA | | N/A | N/A | N/A | 0 | NA | stop-gained | stop// | 0.0001499 |
| exm112227 | rs149019370 | *CD1A* | Nonsense_W31X | chr1:158224908 | 3/8097 | 0/21817 | 0.01984 | A | G | 0/3/8097 | 0/0/21817 | 0/1/3583 | 0/0/10590 | 0/1/3417 | 0/0/9347 | 0/0/195 | 0/0/178 | 0/1/396 | 0/0/376 | 0/0/259 | 0/0/273 | 0/0/247 | 0/0/1053 | NA | | N/A | N/A | N/A | 0 | NA | stop-gained | stop// | 0.0002098 |
| exm1283459 | rs199641371 | *ZNF594* | Nonsense_Q230X | chr17:5086864 | 3/8097 | 0/21820 | 0.01984 | A | G | 0/3/8097 | 0/0/21820 | 0/0/3584 | 0/0/10590 | 0/3/3415 | 0/0/9350 | 0/0/195 | 0/0/178 | 0/0/397 | 0/0/376 | 0/0/259 | 0/0/273 | 0/0/247 | 0/0/1053 | NA | | N/A | N/A | N/A | 0 | NA | stop-gained | stop// | 1.50E-05 |
| exm1295619 | rs200681631 | *DNAH9* | Nonsense_Y1573X | chr17:11597289 | 3/8097 | 0/21819 | 0.01984 | C | A | 0/3/8097 | 0/0/21819 | 0/1/3583 | 0/0/10590 | 0/0/3418 | 0/0/9349 | 0/0/195 | 0/0/178 | 0/1/396 | 0/0/376 | 0/1/258 | 0/0/273 | 0/0/247 | 0/0/1053 | NA | | N/A | N/A | N/A | 0 | NA | stop-gained | stop// | n/a |
| exm1515136 | rs113587027 | *ZNF418* | Nonsense_R664X | chr19:58437559 | 3/8097 | 0/21820 | 0.01984 | A | G | 0/3/8097 | 0/0/21820 | 0/2/3582 | 0/0/10590 | 0/1/3417 | 0/0/9350 | 0/0/195 | 0/0/178 | 0/0/397 | 0/0/376 | 0/0/259 | 0/0/273 | 0/0/247 | 0/0/1053 | NA | | N/A | N/A | N/A | 0 | NA | stop-gained | stop// | 0 |
| exm347715 | rs201216056 | *ABTB1* | Nonsense_C188X | chr3:127395847 | 3/8097 | 0/21816 | 0.01984 | A | T | 0/3/8097 | 0/0/21816 | 0/0/3584 | 0/0/10590 | 0/3/3415 | 0/0/9346 | 0/0/195 | 0/0/178 | 0/0/397 | 0/0/376 | 0/0/259 | 0/0/273 | 0/0/247 | 0/0/1053 | NA | | N/A | N/A | N/A | 0 | NA | stop-gained | stop// | 3.08E-05 |
| exm521348 | rs181930473 | *HIST1H3A* | Nonsense_Q69X | chr6:26020922 | 3/8097 | 0/21820 | 0.01984 | A | G | 0/3/8097 | 0/0/21820 | 0/0/3584 | 0/0/10590 | 0/0/3418 | 0/0/9350 | 0/3/192 | 0/0/178 | 0/0/397 | 0/0/376 | 0/0/259 | 0/0/273 | 0/0/247 | 0/0/1053 | NA | | N/A | N/A | N/A | 0 | NA | stop-gained | stop// | N/A |
| ***Missense mutations*** | | | | | | | | | | | | | | | | | | | | | | | | | | | | | | | | | |
| exm682789 | rs138438915 | *CTSB* | Missense_V249L | chr8:11704609 | 6/8094 | 0/21819 | 0.00039 | A | C | 0/6/8094 | 0/0/21819 | 0/6/3578 | 0/0/10590 | 0/0/3418 | 0/0/9349 | 0/0/195 | 0/0/178 | 0/0/397 | 0/0/376 | 0/0/259 | 0/0/273 | 0/0/247 | 0/0/1053 | 1 | probably damaging | 0.02 | DAMAGING | 2.86 | 0 | 2 | missense | nonsynonymous// | 0.0001799 |
| exm513693 | rs9503910 | *C6orf201* | Missense_D25Y | chr6:4087948 | 5/8094 | 0/21819 | 0.00145 | A | C | 0/5/8094 | 0/0/21819 | 0/1/3582 | 0/0/10589 | 0/1/3417 | 0/0/9350 | 0/0/195 | 0/0/178 | 0/0/397 | 0/0/376 | 0/3/256 | 0/0/273 | 0/0/247 | 0/0/1053 | 1 | probably damaging | 0 | DAMAGING *Warning! Low confidence. | 3.47 | 0 | NA | missense | nonsynonymous// | 7.50E-05 |
| exm126316 | rs146602337 | *SEC16B* | Missense_R142W | chr1:177934291 | 5/8095 | 0/21819 | 0.00145 | A | G | 0/5/8095 | 0/0/21819 | 0/0/3584 | 0/0/10590 | 0/1/3417 | 0/0/9349 | 0/0/195 | 0/0/178 | 0/0/397 | 0/0/376 | 0/3/256 | 0/0/273 | 0/1/246 | 0/0/1053 | 0.878 | possibly damaging | 0.02 | DAMAGING | 2.25 | 0 | NA | missense | nonsynonymous// | 7.86E-05 |
| exm2237096 | rs35038757 | *STK36* | Missense_R240W | chr2:219543924 | 5/8095 | 0/21819 | 0.00145 | A | G | 0/5/8095 | 0/0/21819 | 0/0/3584 | 0/0/10590 | 0/5/3413 | 0/0/9349 | 0/0/195 | 0/0/178 | 0/0/397 | 0/0/376 | 0/0/259 | 0/0/273 | 0/0/247 | 0/0/1053 | 0.976 | probably damaging | 0.02 | DAMAGING | 2.78 | 0 | 2 | missense | nonsynonymous// | 1.50E-05 |
| exm1169305 | rs200881596 | *PIF1* | Missense_R327W | chr15:65113473 | 5/8093 | 0/21803 | 0.00146 | A | G | 0/5/8093 | 0/0/21803 | 0/3/3579 | 0/0/10574 | 0/0/3418 | 0/0/9349 | 0/1/194 | 0/0/178 | 0/0/397 | 0/0/376 | 0/1/258 | 0/0/273 | 0/0/247 | 0/0/1053 | 0.999 | probably damaging | 0 | DAMAGING | 2 | 0 | 2 | missense | nonsynonymous// | 0.000135 |
| exm609279 | rs146913158 | *FAM126A* | Missense_Y64C | chr7:23018030 | 5/8095 | 0/21800 | 0.00146 | G | A | 0/5/8095 | 0/0/21800 | 0/0/3584 | 0/0/10590 | 0/5/3413 | 0/0/9330 | 0/0/195 | 0/0/178 | 0/0/397 | 0/0/376 | 0/0/259 | 0/0/273 | 0/0/247 | 0/0/1053 | 1 | probably damaging | 0 | DAMAGING | 2.49 | 0 | 2 | missense | nonsynonymous// | 0.002269 (1) |
| exm1049488 | rs1472840 | *NCOR2* | Missense_T2199P | chr12:124817806 | 4/8092 | 0/21819 | 0.00536 | C | A | 0/4/8092 | 0/0/21819 | 0/0/3584 | 0/0/10590 | 0/1/3413 | 0/0/9349 | 0/1/194 | 0/0/178 | 0/0/397 | 0/0/376 | 0/2/257 | 0/0/273 | 0/0/247 | 0/0/1053 | 0.998 | probably damaging | 0.01 | DAMAGING | 2.94 | 0 | 2 | missense | nonsynonymous// | 0.0001856 |
| exm141256 | rs201947927 | *MDM4* | Missense_S367L | chr1:204518437 | 4/8094 | 0/21819 | 0.00537 | A | G | 0/4/8094 | 0/0/21819 | 0/1/3582 | 0/0/10590 | 0/0/3417 | 0/0/9349 | 0/2/193 | 0/0/178 | 0/1/396 | 0/0/376 | 0/0/259 | 0/0/273 | 0/0/247 | 0/0/1053 | 0.999 | probably damaging | 0.03 | DAMAGING | 2.43 | 0 | 2 | missense | nonsynonymous// | 7.49E-05 |
| exm97036 | rs115192275 | *CGN* | Missense_V333M | chr1:151492912 | 4/8095 | 0/21820 | 0.00537 | A | G | 0/4/8095 | 0/0/21820 | 0/4/3580 | 0/0/10590 | 0/0/3417 | 0/0/9350 | 0/0/195 | 0/0/178 | 0/0/397 | 0/0/376 | 0/0/259 | 0/0/273 | 0/0/247 | 0/0/1053 | 0.808 | possibly damaging | 0 | DAMAGING | 2.73 | 0 | NA | missense | nonsynonymous// | 0.0001199 |
| exm1292157 | rs140252285 | *KRBA2* | Missense_Q33P | chr17:8274755 | 4/8095 | 0/21818 | 0.00537 | C | A | 0/4/8095 | 0/0/21818 | 0/2/3582 | 0/0/10590 | 0/0/3417 | 0/0/9348 | 0/0/195 | 0/0/178 | 0/1/396 | 0/0/376 | 0/0/259 | 0/0/273 | 0/1/246 | 0/0/1053 | 0.804 | possibly damaging | 0 | DAMAGING *Warning! Low confidence. | 3.3 | 0 | NA | missense | nonsynonymous// | 0.0002547 |
| exm1435795 | rs143659792 | *EMR2* | Missense_G604S | chr19:14862462 | 4/8095 | 0/21817 | 0.00537 | A | G | 0/4/8095 | 0/0/21817 | 0/0/3584 | 0/0/10589 | 0/4/3414 | 0/0/9348 | 0/0/195 | 0/0/178 | 0/0/397 | 0/0/376 | 0/0/258 | 0/0/273 | 0/0/247 | 0/0/1053 | 0.845 | possibly damaging | 0.01 | DAMAGING | 2.31 | 0 | NA | missense | nonsynonymous// | 0 |
| exm1607651 | rs201526262 | *MICALL1* | Missense_R852C | chr22:38336799 | 4/8096 | 0/21820 | 0.00537 | A | G | 0/4/8096 | 0/0/21820 | 0/2/3582 | 0/0/10590 | 0/1/3417 | 0/0/9350 | 0/0/195 | 0/0/178 | 0/0/397 | 0/0/376 | 0/1/258 | 0/0/273 | 0/0/247 | 0/0/1053 | 0.998 | probably damaging | 0.01 | DAMAGING | 3.11 | 0 | 2 | missense | nonsynonymous// | 0 |
| exm39241 | rs77828190 | *SERINC2* | Missense_R454H | chr1:31907027 | 4/8096 | 0/21820 | 0.00537 | A | G | 0/4/8096 | 0/0/21820 | 0/0/3584 | 0/0/10590 | 0/2/3416 | 0/0/9350 | 0/2/193 | 0/0/178 | 0/0/397 | 0/0/376 | 0/0/259 | 0/0/273 | 0/0/247 | 0/0/1053 | 0.913 | possibly damaging | 0.01 | DAMAGING | 2.77 | 0 | NA | missense | nonsynonymous// | 1.51E-05 |
| exm882861 | rs61735506 | *OR51B5* | Missense_S295R | chr11:5461860 | 4/8096 | 0/21819 | 0.00537 | C | A | 0/4/8096 | 0/0/21819 | 0/1/3583 | 0/0/10590 | 0/1/3417 | 0/0/9349 | 0/1/194 | 0/0/178 | 0/1/396 | 0/0/376 | 0/0/259 | 0/0/273 | 0/0/247 | 0/0/1053 | 0.994 | probably damaging | 0.01 | DAMAGING | 2.85 | 0 | 2 | missense | nonsynonymous// | 0.0001949 |
| exm1276720 | rs146563186 | *MNT* | Missense_A348V | chr17:2290901 | 4/8096 | 0/21817 | 0.00537 | A | G | 01/03/8096 | 0/0/21817 | 0/0/3584 | 0/0/10590 | 01/03/3414 | 0/0/9347 | 0/0/195 | 0/0/178 | 0/0/397 | 0/0/376 | 0/0/259 | 0/0/273 | 0/0/247 | 0/0/1053 | 0.982 | probably damaging | 0.04 | DAMAGING | 3.13 | 0 | 2 | missense | nonsynonymous// | 1.51E-05 |
| exm159002 | rs34622148 | *TSNAX-DISC1* | Missense_L330F | chr1:231830492 | 3/8096 | 0/21819 | 0.01983 | A | G | 0/3/8096 | 0/0/21819 | 0/0/3584 | 0/0/10590 | 0/1/3416 | 0/0/9349 | 0/1/194 | 0/0/178 | 0/0/397 | 0/0/376 | 0/0/259 | 0/0/273 | 0/1/246 | 0/0/1053 | 0.999 | probably damaging | 0.01 | DAMAGING | 2.87 | 1 | 2 | missense | nonsynonymous// | 3.09E-05 |
| exm434525 | rs143573166 | *CEP44* | Missense_K119T | chr4:175224972 | 3/8094 | 0/21813 | 0.01983 | C | A | 0/3/8094 | 0/0/21813 | 0/1/3582 | 0/0/10590 | 0/1/3415 | 0/0/9343 | 0/0/195 | 0/0/178 | 0/1/396 | 0/0/376 | 0/0/259 | 0/0/273 | 0/0/247 | 0/0/1053 | 1 | probably damaging | 0.01 | DAMAGING | 2.82 | 0 | 2 | missense | nonsynonymous// | 0.0004243 |
| exm477849 | rs143064419 | *FSTL4* | Missense_G580V | chr5:132537712 | 3/8090 | 0/21803 | 0.01983 | A | C | 0/3/8090 | 0/0/21803 | 0/0/3583 | 0/0/10584 | 0/3/3409 | 0/0/9339 | 0/0/195 | 0/0/178 | 0/0/397 | 0/0/376 | 0/0/259 | 0/0/273 | 0/0/247 | 0/0/1053 | 0.964 | probably damaging | 0.03 | DAMAGING | 2.7 | 0 | 2 | missense | nonsynonymous// | 0 |
| exm1006054 | rs116055718 | *KRT73* | Missense_V243M | chr12:53008455 | 3/8097 | 0/21818 | 0.01984 | A | G | 0/3/8097 | 0/0/21818 | 0/0/3584 | 0/0/10590 | 0/3/3415 | 0/0/9348 | 0/0/195 | 0/0/178 | 0/0/397 | 0/0/376 | 0/0/259 | 0/0/273 | 0/0/247 | 0/0/1053 | 0.772 | possibly damaging | 0.01 | DAMAGING | 3.02 | 0 | NA | missense | nonsynonymous// | 1.54E-05 |
| exm1083572 | rs79276613 | *OR4K5* | Missense_N65K | chr14:20388960 | 3/8097 | 0/21820 | 0.01984 | A | C | 0/3/8097 | 0/0/21820 | 0/0/3584 | 0/0/10590 | 0/2/3416 | 0/0/9350 | 0/0/195 | 0/0/178 | 0/0/397 | 0/0/376 | 0/1/258 | 0/0/273 | 0/0/247 | 0/0/1053 | 1 | probably damaging | 0 | DAMAGING | 2.83 | 0 | 2 | missense | nonsynonymous// | 3.00E-05 |
| exm127544 | rs199933063 | *TOR1AIP1* | Missense_E121K | chr1:179851998 | 3/8097 | 0/21819 | 0.01984 | A | G | 0/3/8097 | 0/0/21819 | 0/3/3581 | 0/0/10590 | 0/0/3418 | 0/0/9349 | 0/0/195 | 0/0/178 | 0/0/397 | 0/0/376 | 0/0/259 | 0/0/273 | 0/0/247 | 0/0/1053 | 1 | probably damaging | 0 | DAMAGING *Warning! Low confidence. | 3.39 | 0 | NA | missense | nonsynonymous// | 0.0007384 |
| exm1362169 | rs201536028 | *ENGASE* | Missense_E237K | chr17:77076432 | 3/8097 | 0/21820 | 0.01984 | A | G | 02/01/8097 | 0/0/21820 | 0/1/3583 | 0/0/10590 | 0/0/3418 | 0/0/9350 | 0/0/195 | 0/0/178 | 2/0/395 | 0/0/376 | 0/0/259 | 0/0/273 | 0/0/247 | 0/0/1053 | 1 | probably damaging | 0 | DAMAGING | 2.57 | 0 | 2 | missense | nonsynonymous// | n/a |
| exm1542518 | rs201841566 | *L3MBTL1* | Missense_Y386F | chr20:42161555 | 3/8097 | 0/21817 | 0.01984 | T | A | 0/3/8097 | 0/0/21817 | 0/1/3583 | 0/0/10590 | 0/0/3418 | 0/0/9347 | 0/0/195 | 0/0/178 | 0/0/397 | 0/0/376 | 0/2/257 | 0/0/273 | 0/0/247 | 0/0/1053 | 0.967 | probably damaging | 0.04 | DAMAGING | 2.55 | 0 | 2 | missense | nonsynonymous// | n/a |
| exm1552938 | rs16981999 | *APCDD1L* | Missense_R261C | chr20:57036571 | 3/8097 | 0/21820 | 0.01984 | A | G | 0/3/8097 | 0/0/21820 | 0/0/3584 | 0/0/10590 | 0/1/3417 | 0/0/9350 | 0/0/195 | 0/0/178 | 0/1/396 | 0/0/376 | 0/0/259 | 0/0/273 | 0/1/246 | 0/0/1053 | 0.999 | probably damaging | 0 | DAMAGING | 2.88 | 0 | 2 | missense | nonsynonymous// | 0.0002678 |
| exm157841 | rs138064546 | *PGBD5* | Missense_P522L | chr1:230459181 | 3/8097 | 0/21818 | 0.01984 | A | G | 3/0/8097 | 0/0/21818 | 3/0/3581 | 0/0/10589 | 0/0/3418 | 0/0/9349 | 0/0/195 | 0/0/178 | 0/0/397 | 0/0/376 | 0/0/259 | 0/0/273 | 0/0/247 | 0/0/1053 | 0.997 | probably damaging | 0 | DAMAGING | 3.03 | 0 | 2 | missense | nonsynonymous// | 3.02E-05 |
| exm2219293 | rs186432117 | *SYT12* | Missense_L368F | chr11:66816064 | 3/8097 | 0/21819 | 0.01984 | A | G | 3/0/8097 | 0/0/21819 | 0/0/3584 | 0/0/10590 | 0/0/3418 | 0/0/9349 | 0/0/195 | 0/0/178 | 0/0/397 | 0/0/376 | 3/0/256 | 0/0/273 | 0/0/247 | 0/0/1053 | 1 | probably damaging | 0.01 | DAMAGING | 2.52 | 0 | 2 | missense | nonsynonymous// | |
| exm240087 | rs137983840 | *LRP2* | Missense_A3344T | chr2:170038097 | 3/8097 | 0/21820 | 0.01984 | A | G | 0/3/8097 | 0/0/21820 | 0/2/3582 | 0/0/10590 | 0/1/3417 | 0/0/9350 | 0/0/195 | 0/0/178 | 0/0/397 | 0/0/376 | 0/0/259 | 0/0/273 | 0/0/247 | 0/0/1053 | 0.996 | probably damaging | 0.04 | DAMAGING | 2.23 | 0 | 2 | missense | nonsynonymous// | 0.0001049 |
| exm303176 | rs200679198 | *ZNF619* | Missense_G464R | chr3:40529271 | 3/8097 | 0/21819 | 0.01984 | A | G | 0/3/8097 | 0/0/21819 | 0/3/3581 | 0/0/10590 | 0/0/3418 | 0/0/9349 | 0/0/195 | 0/0/178 | 0/0/397 | 0/0/376 | 0/0/259 | 0/0/273 | 0/0/247 | 0/0/1053 | 1 | probably damaging | 0 | DAMAGING | 2.59 | 0 | 2 | missense | nonsynonymous// | 6.00E-05 |
| exm391978 | rs149977507 | *KCNIP4* | Missense_R216H | chr4:20731736 | 3/8097 | 0/21820 | 0.01984 | A | G | 0/3/8097 | 0/0/21820 | 0/1/3583 | 0/0/10590 | 0/1/3417 | 0/0/9350 | 0/1/194 | 0/0/178 | 0/0/397 | 0/0/376 | 0/0/259 | 0/0/273 | 0/0/247 | 0/0/1053 | 0.634 | possibly damaging | 0.04 | DAMAGING | 3 | 0 | NA | missense | nonsynonymous// | |
| exm417917 | rs141061981 | *LEF1* | Missense_E71Q | chr4:109088713 | 3/8097 | 0/21820 | 0.01984 | G | C | 0/3/8097 | 0/0/21820 | 0/3/3581 | 0/0/10590 | 0/0/3418 | 0/0/9350 | 0/0/195 | 0/0/178 | 0/0/397 | 0/0/376 | 0/0/259 | 0/0/273 | 0/0/247 | 0/0/1053 | 0.876 | possibly damaging | 0.02 | DAMAGING *Warning! Low confidence. | 3.27 | 0 | NA | missense | nonsynonymous// | 1.50E-05 |
| exm43432 | rs145749755 | *ZMYM6* | Missense_Y106C | chr1:35485065 | 3/8097 | 0/21820 | 0.01984 | G | A | 0/3/8097 | 0/0/21820 | 0/3/3581 | 0/0/10590 | 0/0/3418 | 0/0/9350 | 0/0/195 | 0/0/178 | 0/0/397 | 0/0/376 | 0/0/259 | 0/0/273 | 0/0/247 | 0/0/1053 | 0.999 | probably damaging | 0 | DAMAGING | 2.74 | 0 | 2 | missense | nonsynonymous// | 0.0002847 |
| exm46136 | rs182482113 | *RSPO1* | Missense_R220W | chr1:38078561 | 3/8097 | 0/21817 | 0.01984 | A | G | 0/3/8097 | 0/0/21817 | 0/0/3584 | 0/0/10590 | 0/2/3416 | 0/0/9347 | 0/0/195 | 0/0/178 | 0/0/397 | 0/0/376 | 0/1/258 | 0/0/273 | 0/0/247 | 0/0/1053 | 0.993 | probably damaging | 0 | DAMAGING | 2.92 | 0 | 2 | missense | nonsynonymous// | 6.00E-05 |
| exm652229 | rs75961395 | *CFTR* | Missense_G85E | chr7:117149177 | 3/8097 | 0/21820 | 0.01984 | A | G | 0/3/8097 | 0/0/21820 | 0/1/3583 | 0/0/10590 | 0/0/3418 | 0/0/9350 | 0/1/194 | 0/0/178 | 0/0/397 | 0/0/376 | 0/1/258 | 0/0/273 | 0/0/247 | 0/0/1053 | 0.995 | probably damaging | 0.01 | DAMAGING | 2.47 | 3 | 2 | missense | nonsynonymous// | 0.00012 |
| exm652595 | rs199687888 | *CTTNBP2* | Missense_R948Q | chr7:117407166 | 3/8097 | 0/21820 | 0.01984 | A | G | 0/3/8097 | 0/0/21820 | 0/0/3584 | 0/0/10590 | 0/0/3418 | 0/0/9350 | 0/2/193 | 0/0/178 | 0/0/397 | 0/0/376 | 0/1/258 | 0/0/273 | 0/0/247 | 0/0/1053 | 1 | probably damaging | 0.01 | DAMAGING | 2.92 | 0 | 2 | missense | nonsynonymous// | 0 |
| exm726831 | rs113705108 | *MROH6* | Missense_R377P | chr8:144652149 | 3/8097 | 0/21820 | 0.01984 | G | C | 0/3/8097 | 0/0/21820 | 0/0/3584 | 0/0/10590 | 0/1/3417 | 0/0/9350 | 0/0/195 | 0/0/178 | 0/0/397 | 0/0/376 | 0/2/257 | 0/0/273 | 0/0/247 | 0/0/1053 | 1 | probably damaging | 0 | DAMAGING | 1.71 | 0 | 2 | missense | nonsynonymous// | |
| exm755882 | rs114234833 | *PCSK5* | Missense_S1458F | chr9:78943039 | 3/8097 | 0/21816 | 0.01984 | A | G | 0/3/8097 | 0/0/21816 | 0/0/3584 | 0/0/10590 | 0/0/3418 | 0/0/9346 | 0/2/193 | 0/0/178 | 0/0/397 | 0/0/376 | 0/1/258 | 0/0/273 | 0/0/247 | 0/0/1053 | 0.886 | possibly damaging | 0.05 | DAMAGING | 1.74 | 0 | NA | missense | nonsynonymous// | 0.0003338 |
| exm772157 | rs201244484 | *KIAA0368* | Missense_R708C | chr9:114180258 | 3/8097 | 0/21819 | 0.01984 | A | G | 0/3/8097 | 0/0/21819 | 0/2/3582 | 0/0/10590 | 0/1/3417 | 0/0/9349 | 0/0/195 | 0/0/178 | 0/0/397 | 0/0/376 | 0/0/259 | 0/0/273 | 0/0/247 | 0/0/1053 | 0.999 | probably damaging | 0.01 | DAMAGING | 2.12 | 0 | 2 | missense | nonsynonymous// | 2.54E-05 |
| exm864262 | rs144737013 | *C10orf90* | Missense_F483S | chr10:128153351 | 3/8097 | 0/21820 | 0.01984 | G | A | 0/3/8097 | 0/0/21820 | 0/1/3583 | 0/0/10590 | 0/0/3418 | 0/0/9350 | 0/0/195 | 0/0/178 | 0/0/397 | 0/0/376 | 0/1/258 | 0/0/273 | 0/1/246 | 0/0/1053 | 0.606 | possibly damaging | 0.04 | DAMAGING | 2.89 | 0 | NA | missense | nonsynonymous// | 0.0002847 |
| exm881231 | rs200807047 | *OR51E1* | Missense_R125C | chr11:4674129 | 3/8097 | 0/21820 | 0.01984 | A | G | 0/3/8097 | 0/0/21820 | 0/2/3582 | 0/0/10590 | 0/0/3418 | 0/0/9350 | 0/1/194 | 0/0/178 | 0/0/397 | 0/0/376 | 0/0/259 | 0/0/273 | 0/0/247 | 0/0/1053 | 1 | probably damaging | 0 | DAMAGING | 2.78 | 0 | 2 | missense | nonsynonymous// | 0.0001363 |
| exm896540 | rs200601314 | *NELL1* | Missense_R415C | chr11:20968969 | 3/8097 | 0/21819 | 0.01984 | A | G | 0/3/8097 | 0/0/21819 | 0/1/3583 | 0/0/10590 | 0/0/3418 | 0/0/9349 | 0/0/195 | 0/0/178 | 0/0/397 | 0/0/376 | 0/2/257 | 0/0/273 | 0/0/247 | 0/0/1053 | 0.64 | possibly damaging | 0.01 | DAMAGING | 2.2 | 0 | NA | missense | nonsynonymous// | |
| exm907999 | rs145838163 | *OR5D14* | Missense_H254R | chr11:55563792 | 3/8097 | 0/21819 | 0.01984 | G | A | 0/3/8097 | 0/0/21819 | 0/0/3584 | 0/0/10590 | 0/3/3415 | 0/0/9349 | 0/0/195 | 0/0/178 | 0/0/397 | 0/0/376 | 0/0/259 | 0/0/273 | 0/0/247 | 0/0/1053 | 0.969 | probably damaging | 0 | DAMAGING | 2.78 | 0 | 2 | missense | nonsynonymous// | 0 |
| exm937175 | rs115978536 | *SHANK2* | Missense_P2L | chr11:70858368 | 3/8097 | 0/21819 | 0.01984 | A | G | 0/3/8097 | 0/0/21819 | 0/0/3584 | 0/0/10590 | 0/0/3418 | 0/0/9349 | 0/2/193 | 0/0/178 | 0/0/397 | 0/0/376 | 0/1/258 | 0/0/273 | 0/0/247 | 0/0/1053 | 1 | probably damaging | 0.01 | DAMAGING | 3.02 | 0 | 2 | missense | nonsynonymous// | 0 |
| exm1042718 | rs201734893 | *GCN1L1* | Missense_R2107H | chr12:120575778 | 3/8095 | 0/21808 | 0.01985 | A | G | 0/3/8095 | 0/0/21808 | 0/1/3583 | 0/0/10590 | 0/0/3416 | 0/0/9338 | 0/2/193 | 0/0/178 | 0/0/397 | 0/0/376 | 0/0/259 | 0/0/273 | 0/0/247 | 0/0/1053 | 0.996 | probably damaging | 0.01 | DAMAGING | 2.14 | 0 | 2 | missense | nonsynonymous// | 0.0005024 |
| exm1207590 | rs147234069 | *CCNF* | Missense_R239Q | chr16:2489766 | 3/8097 | 0/21812 | 0.01985 | A | G | 0/3/8097 | 0/0/21812 | 0/0/3584 | 0/0/10590 | 0/3/3415 | 0/0/9342 | 0/0/195 | 0/0/178 | 0/0/397 | 0/0/376 | 0/0/259 | 0/0/273 | 0/0/247 | 0/0/1053 | 0.997 | probably damaging | 0.03 | DAMAGING | 2.86 | 0 | 2 | missense | nonsynonymous// | 3.04E-05 |
| exm1559083 | rs146772563 | *PTK6* | Missense_G321R | chr20:62162152 | 3/8097 | 0/21812 | 0.01985 | A | G | 02/01/8097 | 0/0/21812 | 0/0/3584 | 0/0/10590 | 0/0/3418 | 0/0/9342 | 0/1/194 | 0/0/178 | 0/0/397 | 0/0/376 | 2/0/257 | 0/0/273 | 0/0/247 | 0/0/1053 | 0.999 | probably damaging | 0 | DAMAGING | 3.25 | 0 | 2 | missense | nonsynonymous// | 1.51E-05 |
| exm858712 | rs62641727 | *C10orf82* | Missense_T74M | chr10:118425172 | 3/8097 | 0/21815 | 0.01985 | A | G | 0/3/8097 | 0/0/21815 | 0/1/3583 | 0/0/10590 | 0/0/3418 | 0/0/9345 | 0/0/195 | 0/0/178 | 0/0/397 | 0/0/376 | 0/2/257 | 0/0/273 | 0/0/247 | 0/0/1053 | 0.879 | possibly damaging | 0.03 | DAMAGING | 2.82 | 0 | NA | missense | nonsynonymous// | 0.000105 |
| exm865091 | rs148481888 | *MKI67* | Missense_R2189C | chr10:129902459 | 3/8097 | 0/21809 | 0.01986 | A | G | 0/3/8097 | 0/0/21809 | 0/0/3584 | 0/0/10590 | 0/0/3418 | 0/0/9339 | 0/0/195 | 0/0/178 | 0/0/397 | 0/0/376 | 0/2/257 | 0/0/273 | 0/1/246 | 0/0/1053 | 1 | probably damaging | 0.05 | DAMAGING | 1.4 | 0 | 2 | missense | nonsynonymous// | 1.50E-05 |
| **(2) Candidate recessive high-penetrance CRC alleles.** | | | | | | | | | | | | | | | | | | | | | | | | | | | | | | | | | |
| ***Stop mutations*** | | | | | | | | | | | | | | | | | | | | | | | | | | | | | | | | | |
| exm1204998 | rs150766139 | *NTHL1* | Nonsense_Q90X | chr16:2096239 | 3/8097 | 0/21820 | 0.01984 | A | G | 3/32/8065 | 0/57/21763 | 0/18/3566 | 0/34/10556 | 0/9/3409 | 0/13/9337 | 0/2/193 | 0/1/177 | 3/1/393 | 0/6/370 | 0/2/257 | 0/2/271 | 0/0/247 | 0/1/1052 | NA | | N/A | N/A | N/A | 0 | NA | stop-gained | stop// | 0.002304 |
| ***Missense mutations*** | | | | | | | | | | | | | | | | | | | | | | | | | | | | | | | | | |
| exm252852 | rs61756360 | *PMS1* | Missense_T75I | chr2:190660586 | 4/8096 | 0/21817 | 0.00537 | A | G | 4/47/8049 | 0/93/21724 | 1/16/3567 | 0/46/10544 | 3/26/3389 | 0/45/9302 | 0/1/194 | 0/0/178 | 0/0/397 | 0/0/376 | 0/4/255 | 0/1/272 | 0/0/247 | 0/1/1052 | 1 | probably damaging | 0 | DAMAGING | 3.19 | 0 | 2 | missense | nonsynonymous// | 0.0008096 |
| exm970236 | rs78900720 | *PRDM10* | Missense_T59S | chr11:129827700 | 4/8096 | 0/21814 | 0.00537 | A | T | 4/31/8065 | 0/42/21772 | 0/9/3575 | 0/11/10579 | 04/11/3403 | 0/23/9321 | 0/9/186 | 0/1/177 | 0/0/397 | 0/0/376 | 0/2/257 | 0/5/268 | 0/0/247 | 0/2/1051 | 0.997 | probably damaging | 0.01 | DAMAGING *Warning! Low confidence. | 3.97 | 0 | NA | missense | nonsynonymous// | 0.001472 |
| exm1549703 | rs116905185 | *FAM65C* | Missense_D318G | chr20:49221303 | 3/8097 | 0/21820 | 0.01984 | G | A | 3/182/7915 | 0/440/21380 | 1/79/3504 | 0/232/10358 | 1/79/3338 | 0/183/9167 | 0/4/191 | 0/3/175 | 1/12/384 | 0/7/369 | 0/4/255 | 0/4/269 | 0/4/243 | 0/11/1042 | 0.999 | probably damaging | 0.02 | DAMAGING | 2.34 | 0 | 2 | missense | nonsynonymous// | 0.006 (3 ) |
| exm157841 | rs138064546 | *PGBD5* | Missense_P522L | chr1:230459181 | 3/8097 | 0/21818 | 0.01984 | A | G | 3/0/8097 | 0/0/21818 | 3/0/3581 | 0/0/10589 | 0/0/3418 | 0/0/9349 | 0/0/195 | 0/0/178 | 0/0/397 | 0/0/376 | 0/0/259 | 0/0/273 | 0/0/247 | 0/0/1053 | 0.997 | probably damaging | 0 | DAMAGING | 3.03 | 0 | 2 | missense | nonsynonymous// | 3.02E-05 |
| exm2219293 | rs186432117 | *SYT12* | Missense_L368F | chr11:66816064 | 3/8097 | 0/21819 | 0.01984 | A | G | 3/0/8097 | 0/0/21819 | 0/0/3584 | 0/0/10590 | 0/0/3418 | 0/0/9349 | 0/0/195 | 0/0/178 | 0/0/397 | 0/0/376 | 3/0/256 | 0/0/273 | 0/0/247 | 0/0/1053 | 1 | probably damaging | 0.01 | DAMAGING | 2.52 | 0 | 2 | missense | nonsynonymous// | |
| exm270161 | rs140297559 | *SLC4A3* | Missense_R917C | chr2:220502516 | 3/8097 | 0/21819 | 0.01984 | A | G | 03/01/8096 | 0/3/21816 | 2/0/3582 | 0/1/10589 | 0/1/3417 | 0/2/9347 | 0/0/195 | 0/0/178 | 0/0/397 | 0/0/376 | 0/0/259 | 0/0/273 | 1/0/246 | 0/0/1053 | 0.998 | probably damaging | 0 | DAMAGING | 2.71 | 9 | 2 | missense | nonsynonymous// | 0.0002706 |
| exm700363 | rs200978094 | *PXDNL* | Missense_R1181I | chr8:52320642 | 3/8097 | 0/21820 | 0.01984 | A | C | 3/0/8097 | 0/1/21819 | 1/0/3583 | 0/0/10590 | 0/0/3418 | 0/1/9349 | 0/0/195 | 0/0/178 | 0/0/397 | 0/0/376 | 2/0/257 | 0/0/273 | 0/0/247 | 0/0/1053 | 0.927 | possibly damaging | 0.02 | DAMAGING | 2.61 | 0 | NA | missense | nonsynonymous// | 8.14E-05 |
| exm297309 | rs201860756 | *TRIM71* | Missense_G597S | chr3:32932485 | 3/8097 | 0/21813 | 0.01985 | A | G | 3/0/8097 | 0/2/21811 | 1/0/3583 | 0/1/10589 | 0/0/3418 | 0/1/9342 | 2/0/193 | 0/0/178 | 0/0/397 | 0/0/376 | 0/0/259 | 0/0/273 | 0/0/247 | 0/0/1053 | 0.969 | probably damaging | 0.05 | DAMAGING | 1.51 | 0 | 2 | missense | nonsynonymous// | 0.0001505 |

*Dominant alleles (1) were filtered from the entire variant set as follows: predicted not to be benign/tolerated by both SIFT and PP2 or nonsense variants ; excluded probable miscalled SNPs through visual inspection of genotyping clusters; absent in controls to ensure inclusion of potentially high penetrance risk alleles. Recessive alleles (2) were filtered from the entire variant set as follows: predicted not benign or tolerated by both SIFT and PP2; excluded probable miscalled SNPs through visual inspection of genotyping; homozygotes absent in controls to ensure inclusion of potentially high penetrance risk alleles; minor allele frequency =< 0.02 in controls.*

**  Exome Aggregation Consortium (ExAC), Cambridge, MA (URL: http://exac.broadinstitute.org) [July 2015 accessed].*

**Supplementary Table 19. Homozygous rare damaging allele variants in base-excision and mismatch repair pathways.**

| SNP | rs_number | Gene | CHR | Effect allele | Reference allele | AFF (homozygous effect allele genotype/heterozygous + reference allele homozygous genotypes ) | UNAFF (homozygous effect allele genotype/heterozygous + reference allele homozygous genotypes ) | ? (Fisher exact test) | GENO.cases | ENO.contro | NGLAND.cas | LAND.cont | cotland.cas | otland.contr | RTUGAL.ca | TUGAL.cont | OLLAND.cas | LLAND.cont | SPAIN.cases | PAIN.contro | RMANY.cas | MANY.cont | ExAC freq (EUR,non-Finnish)* | ExAC number of homozygous alleles /alllele number ((EUR,non-Finnish)* |
|---|---|---|---|---|---|---|---|---|---|---|---|---|---|---|---|---|---|---|---|---|---|---|---|---|
| **Base excision repair pathway (GO:0006284)** | | | | | | | | | | | | | | | | | | | | | | | | |
| exm1204998 | rs150766139 | *NTHL1* | 16 | A | G | == 3/8097 | == 0/21820 | 0.01984 | 3/32/8065 | 0/57/2176 | 0/18/3566 | 0/34/1055 | 0/9/3409 | 0/13/9337 | 0/2/193 | 0/1/177 | 3/1/393 | 0/6/370 | 0/2/257 | 0/2/271 | 0/0/247 | 0/1/1052 | 0.002304 | 0/65960 |
| exm54989 | rs36053993 | *MUTYH* | 1 | A | G | == 4/8096 | == 1/21819 | 0.02103 | 4/132/796 | 1/277/215 | 3/56/3525 | 0/107/104 | 1/57/3360 | 1/148/920 | 0/7/188 | 0/5/173 | 0/5/392 | 0/1/375 | 0/6/253 | 0/10/263 | 0/1/246 | 0/6/1047 | 0.003958 | 2/65440 |
| exm1204981 | rs1805378 | *NTHL1* | 16 | G | A | == 1/7840 | == 0/21547 | 0.2668 | 1/28/7812 | 0/50/2149 | 1/15/3568 | 0/28/1056 | 0/11/3407 | 0/16/9334 | 0/2/193 | 0/1/177 | 0/0/397 | 0/1/375 | NA | NA | 0/0/247 | 0/4/1049 | 0.003004 | 1/64586 |
| exm288235 | rs104893751 | *OGG1* | 3 | A | G | == 1/8099 | == 0/21819 | 0.2707 | 8022/77/1 | 21617/202/ | 3550/33/1 | 10495/95/( | 3390/28/0 | 9256/93/0 | 194/1/0 | 175/3/0 | 390/7/0 | 368/8/0 | 254/5/0 | 272/1/0 | 244/3/0 | 1051/2/0 | 0.003229 | 0/66270 |
| exm288284 | rs113561019 | *OGG1* | 3 | A | G | == 1/8099 | == 0/21818 | 0.2707 | 8016/83/1 | 21576/242/ | 3544/39/1 | 10477/113/ | 3390/28/0 | 9251/98/0 | 191/4/0 | 172/6/0 | 391/6/0 | 370/5/0 | 256/3/0 | 268/5/0 | 244/3/0 | 1038/15/0 | 0.006295 | 3/66718 |
| exm1204957 | rs146347092 | *NTHL1* | 16 | A | G | == 1/8099 | == 0/21814 | 0.2708 | ######## | 0/33/2178 | 10/2/3582 | 0/7/10583 | ######## | 0/26/9318 | 0/0/195 | 0/0/178 | 0/0/397 | 0/0/376 | 0/0/259 | 0/0/273 | 0/0/247 | 0/0/1053 | 0.0001856 | 0/64642 |
| exm824096 | rs142580756 | *ERCC6* | 10 | A | G | == 1/8098 | == 0/21814 | 0.2708 | ######## | 0/26/2178 | ######## | 0/12/1057 | 0/1/3416 | 0/12/9337 | 0/0/195 | 0/0/178 | 0/1/396 | 0/2/374 | 0/0/259 | 0/0/273 | 0/0/247 | 0/0/1053 | 0.001171 | 0/66612 |
| exm698694 | rs147215490 | *POLB* | 8 | G | A | == 1/8082 | == 0/21736 | 0.2711 | ######## | 0/7/21729 | 0/0/3581 | 0/0/10577 | ######## | 0/2/9288 | 0/0/195 | 0/0/178 | 0/0/397 | 0/1/371 | 0/0/259 | 0/0/273 | 0/2/244 | 0/4/1042 | 0.002223 | 2/66576 |
| exm694461 | rs78488552 | *WRN* | 8 | C | G | == 1/8099 | == 1/21809 | 0.4683 | 8002/97/1 | 21528/281/ | 3533/51/0 | 10435/154/ | 3383/35/0 | 9237/103/( | 192/3/0 | 176/2/0 | 394/2/1 | 368/8/0 | 256/3/0 | 270/3/0 | 244/3/0 | 1042/11/0 | 0.00468 | 3/66666 |
| exm891143 | rs34511735 | *USP47* | 11 | G | C | == 2/8098 | == 5/21806 | 1 | 2/309/778 | 5/815/209 | 0/119/346 | 3/378/102 | 2/156/326 | 2/371/896 | 0/6/189 | 0/7/171 | 0/12/385 | 0/15/361 | 0/8/251 | 0/10/263 | 0/8/239 | 0/34/1019 | 0.01807 | 12/66508 |
| **Mismatch repair pathway (GO:0006298)** | | | | | | | | | | | | | | | | | | | | | | | | |
| exm252852 | rs61756360 | *PMS1* | 2 | A | G | == 4/8096 | == 0/21817 | 0.005371 | 4/47/8049 | 0/93/2172 | 1/16/3567 | 0/46/1054 | 3/26/3389 | 0/45/9302 | 0/1/194 | 0/0/178 | 0/0/397 | 0/0/376 | 0/4/255 | 0/1/272 | 0/0/247 | 0/1/1052 | 0.0008096 | 0/66700 |
| exm54989 | rs36053993 | *MUTYH* | 1 | A | G | == 4/8096 | == 1/21819 | 0.02103 | 4/132/796 | 1/277/215 | 3/56/3525 | 0/107/104 | 1/57/3360 | 1/148/920 | 0/7/188 | 0/5/173 | 0/5/392 | 0/1/375 | 0/6/253 | 0/10/263 | 0/1/246 | 0/6/1047 | 0.003958 | 2/65440 |
| exm603891 | rs200513014 | *PMS2* | 7 | G | A | == 1/8099 | == 0/21818 | 0.2707 | ######## | 0/15/2180 | ######## | 0/4/10586 | 0/2/3416 | 0/9/9339 | 0/0/195 | 0/0/178 | 0/0/397 | 0/1/375 | 0/1/258 | 0/0/273 | 0/0/247 | 0/1/1052 | 0.0004118 | 0/65558 |
| exm69401 | rs5745459 | *MSH4* | 1 | G | A | == 2/8098 | == 4/21812 | 0.665 | 2/183/791 | 4/463/213 | 1/78/3505 | 2/237/103 | 1/85/3332 | 2/202/914 | 0/3/192 | 0/0/178 | 0/11/386 | 0/9/367 | 0/1/258 | 0/1/272 | 0/5/242 | 0/14/1039 | 0.0123 | 2/65446 |

* *Exome Aggregation Consortium (ExAC), Cambridge, MA (URL: http://exac.broadinstitute.org) [July 2015 accessed].*

**Supplementary Table 20. Candidate compound heterozygous high-penetrance CRC alleles**

| ***Gene*** | ***Number of compound heterozygous cases/gene*** | **SNP** | **rsID** | **Position** | **A1** | **A2** | **Count.cases** | **Count.controls** | **Mutation** | **EAF (cases/controls)** | **ExAC freq (EUR,non-Finnish)*** |
|---|---|---|---|---|---|---|---|---|---|---|---|
| *NOTCH2* | 2 | exm89497 | rs35586704 | chr1:120458122 | A | T | 0/78/8022 | 0/203/21609 | Missense_L2408H | 0.004815/0.004653 | 0.002579 |
| | | exm89650 | rs147223770 | chr1:120478125 | C | A | 0/49/8051 | 0/117/21703 | Missense_F1209V | 0.003025/0.002681 | 0.004586 |
| *DNAJC17* | 2 | exm1149787 | rs140603715 | chr15:41060221 | G | A | 0/53/8044 | 0/145/21671 | Missense_M278V | 0.003273/0.003323 | 0.002193 |
| | | exm1149789 | rs186113485 | chr15:41062758 | A | G | 0/4/8093 | 0/12/21803 | Missense_R22Q | 0.000247/0.000275 | 0.0005519 |

*probable miscalled SNPs through visual inspection of genotyping clusters, (3) number of rare damaging heterozygotes per gene in controls <<=1, (4) minor allele frequency <=< 0.02 in controls.*

*EAF=effect allele frequency*

** Exome Aggregation Consortium (ExAC), Cambridge, MA (URL: http://exac.broadinstitute.org) [July 2015 accessed].*
